# Mitochondrial dysfunction and the regulatory cell death crosstalk network in chronic obstructive pulmonary disease: from oxidative stress mechanisms to targeted therapeutic strategies

**DOI:** 10.3389/fimmu.2026.1856853

**Published:** 2026-07-06

**Authors:** Hongyan Fan, Juandi Xue, Jianghua Si

**Affiliations:** The First People's Hospital of Lanzhou (The Second Clinical Medical College of Gansu University of Chinese Medicine), Lanzhou, Gansu, China

**Keywords:** chronic obstructive pulmonary disease (COPD), mitochondrial dysfunction, mitochondrial quality control, oxidative stress, pyroptosis, regulated cell death (RCD)

## Abstract

The sustained progression of chronic obstructive pulmonary disease (COPD) may not be independently driven by a single process such as chronic inflammation, oxidative stress, or cell death, but rather originates from a cross-amplification network among “mitochondrial dysfunction-oxidative stress-regulated cell death.” In the context of mitochondrial damage, excessive generation of reactive oxygen species (ROS), damage and release of mitochondrial DNA (mtDNA), and dysregulation of mitochondrial quality control (MQC) collectively promote airway epithelial injury, sustained inflammation, alveolar destruction, and tissue remodeling. Furthermore, regulated cell death modalities such as apoptosis, necroptosis, pyroptosis, and ferroptosis are not isolated from each other but are coupled under a shared context of mitochondrial stress, exhibiting different dominant patterns across various cell types and disease stages. Adopting an integrated perspective encompassing mitochondrial dysfunction, amplified oxidative stress, and the regulated cell death (RCD) cross-network, this article synthesizes current research regarding COPD-related mechanisms, with a focus on mitochondrial damage markers, RCD activity indicators, mechanism-oriented patient stratification, and potential therapeutic strategies targeting mitochondrial homeostasis and cell death pathways. This framework facilitates the transition of COPD understanding from the traditional chronic inflammation model to a more stratified and translationally promising mitochondrial-cell death network model.

## 1 Introduction

Chronic obstructive pulmonary disease (COPD) is one of the major chronic respiratory diseases globally, leading to morbidity, disability, and mortality, with a high burden of illness and significant clinical heterogeneity (1, 2). Although cigarette smoking is still considered the most important risk factor for COPD, a growing number of studies have shown that a considerable proportion of COPD cases occur in non-smokers, suggesting that its development and progression are not solely driven by smoking but are associated with multiple factors such as air pollution, occupational exposure, and adverse early-life exposures (3–5). These factors can jointly contribute to the occurrence and progression of COPD by influencing lung development trajectories, redox homeostasis, and immune-inflammatory responses, indicating that the traditional framework of explaining the disease based on a single exposure factor is insufficient to fully capture its complex pathological basis (6).

In the classical pathogenesis of COPD, oxidative stress and chronic inflammation have long been considered core mechanisms (7). Cigarette smoke, ozone, fine particulate matter, biomass fuel smoke, and other harmful exposures can trigger the excessive production of reactive oxygen species (ROS), leading to airway epithelial injury, persistent activation of inflammation, destruction of alveolar structures, and progressive worsening of airflow limitation (8–10). However, the continuous progression of COPD cannot be fully explained by a single inflammatory pathway or a single oxidative stress event. A more reasonable understanding is that oxidative stress, chronic inflammation, cell aging, and regulated cell death interact under the common background of organelle stress, forming a continuously amplifying pathological network during disease progression (11, 12). Therefore, identifying common pathological hubs that connect these processes is of great significance for understanding the chronic progression and multi-system involvement of COPD.

Mitochondrial research entered the field of COPD initially mainly from observations of tobacco smoke-induced oxidative damage and energy metabolism abnormalities. Subsequent studies gradually found that in COPD-related airway epithelial cells, alveolar cells, immune cells, and skeletal muscle, changes such as mitochondrial morphological abnormalities, decreased respiratory chain function, loss of membrane potential, elevated mtROS, and mitochondrial DNA damage can occur (13, 14). These findings have expanded the mechanistic understanding of COPD from the traditional “inflammation-oxidative stress” model to an integrated framework of “mitochondrial stress-quality control imbalance-inflammation amplification-cell fate alteration.” Recent studies on mitochondrial dysfunction and integrated cell death patterns have also provided new theoretical support for understanding how mitochondrial damage connects inflammation, oxidative stress, and multiple forms of regulated cell death (15).

In recent years, the role of mitochondrial dysfunction in COPD has received widespread attention (16). Mitochondria are not only the core sites for cellular energy metabolism and redox reactions but also important hubs integrating stress perception, inflammatory signal transduction, and cell fate regulation (17, 18). During mitochondrial dysfunction, excessive ROS generation, mitochondrial DNA (mtDNA) damage and release, decreased membrane potential, and imbalance in mitochondrial quality control (MQC) can jointly drive airway epithelial injury, maintenance of inflammation, cell aging, and tissue remodeling (19, 20). Meanwhile, regulated cell death modes such as apoptosis, necroptosis, pyroptosis, and ferroptosis are increasingly recognized as involved in the pathological process of COPD, forming a mutually reinforcing cross-network with mitochondrial damage and oxidative stress (21). Therefore, mitochondrial dysfunction may be an important pathological hub connecting harmful exposures, oxidative stress, sustained inflammation, tissue destruction, and the cell death network (22).

Building on this understanding, this article reviews the progress of COPD-related mechanistic research from the perspective of the “mitochondrial dysfunction-oxidative stress-regulated cell death” cross-network. This review first outlines mitochondrial structural and functional abnormalities in COPD, including changes in mitochondrial morphology, respiratory chain dysfunction, mtDNA damage, and the characteristics of mitochondrial involvement in different tissues; then discusses the dysregulation of mitochondrial quality control mechanisms, focusing on mitochondrial biogenesis, mitophagy, and the inflammatory network mediated by mitochondrial damage-associated molecular patterns (DAMPs); the third part analyzes the oxidative stress amplification loop, elaborating on ROS sources, mtROS-mediated mitochondrial damage, and Nrf2-related antioxidant defense imbalance; the fourth part discusses the interplay among various regulated cell death modes, further emphasizing the RCD characteristics of different cell types and disease stages; finally, it summarizes potential therapeutic strategies targeting mitochondrial homeostasis and RCD pathways, and discusses key challenges in patient stratification, biomarkers, and clinical translation. Through this structure, this article aims to provide a more integrated mitochondrial-cell death network framework for understanding COPD mechanisms and potential precision interventions.

## 2 Initial mitochondrial injury: the starting point of the COPD pathological network

The “mitochondrial hub” does not imply that mitochondrial dysfunction is the sole or sufficient initial cause of COPD, but rather emphasizes its role in connecting and amplifying multiple pathological processes. Mitochondria can sense stimuli such as cigarette smoke, PM2.5, inflammatory cytokines, oxidative load, and metabolic stress, and further influence oxidative stress, inflammatory activation, and cell death programs through mtROS generation, decreased membrane potential, mtDNA damage and release, imbalance in mitochondrial quality control, and DAMPs signaling (23, 24). Therefore, mitochondrial injury may have a strong driving effect in certain cell types or disease stages, while in other contexts, it is more suitable as a contributing factor that mutually promotes inflammation, oxidative stress, and tissue damage. Based on this definition, this article regards mitochondrial structural and functional abnormalities as an important integrative platform in the COPD pathological network, providing a common organelle basis for subsequent oxidative stress amplification, inflammatory activation, and initiation of cell death programs.

### 2.1 Mitochondrial morphological changes

Morphological abnormalities such as mitochondrial fragmentation, swelling, and cristae structural disruption have been reported in airway epithelial cells, alveolar epithelial cells, and cells of the pulmonary vasculature of COPD patients. These changes are important morphological evidence of impaired mitochondrial function (25, 26). Smoking and the oxidative stress it induces are considered key factors driving these changes. Under conditions of sustained mitochondrial stress, the originally relatively stable mitochondrial network can undergo fragmentation and remodeling, manifesting as reduced mitochondrial volume, irregular morphology, damaged cristae structure, and decreased network continuity (27).

At the molecular level, mitochondrial morphological homeostasis depends on the dynamic balance between fusion and fission processes. Fusion-related proteins mainly include Mitofusin 1/2 (MFN1/2) and optic atrophy 1 (OPA1), while the fission process is primarily regulated by dynamin-related protein 1 (DRP1). Existing studies in COPD lung tissue, alveolar epithelial cells, or smoke exposure models have reported a decrease in fusion-related proteins such as MFN2 and OPA1, as well as a trend of enhanced DRP1-related fission signaling (28–30). These changes suggest that mitochondrial dynamics may shift towards enhanced fission and impaired fusion, and are associated with mitochondrial fragmentation, cristae disorganization, and decreased membrane potential.

In addition to pulmonary cells, abnormalities in mitochondrial density, morphology, and dynamics regulation have also been reported in COPD-related skeletal muscle, suggesting that mitochondrial structural changes are not limited to local lung tissue but may contribute to the development of COPD extrapulmonary phenotypes (31, 32). It should be noted that differences exist between studies in terms of sample source, COPD severity, smoking status, detection methods, and statistical reporting methods. Therefore, molecular changes from a single study should not be directly extrapolated to all COPD patients. Overall, mitochondrial fragmentation, swelling, and cristae disruption constitute important morphological features of mitochondrial abnormalities in COPD, providing a structural basis for subsequent respiratory chain dysfunction, enhanced oxidative stress, and dysregulation of cell fate (33, 34). These conclusions still need to be interpreted in conjunction with the sample size, effect size, and statistical results of specific studies.

### 2.2 Respiratory chain function and energy metabolism disorders

Respiratory chain dysfunction and energy metabolism disorders are important functional manifestations of mitochondrial abnormalities in COPD, characterized by decreased activity of electron transport chain complexes, reduced mitochondrial membrane potential (ΔΨm), insufficient ATP production, and abnormally elevated mitochondrial reactive oxygen species (mtROS) (35). As an important subunit of mitochondrial respiratory chain complex I, NDUFS2 has been reported in COPD lung macrophage-related studies to be associated with impaired mitochondrial respiratory function, oxidative phosphorylation capacity, and phagocytic function (36). These findings suggest that respiratory chain abnormalities not only represent energy metabolism disorders but may also affect the function of pulmonary immune cells.

Furthermore, persistent harmful exposures such as smoking can directly damage mitochondrial DNA and respiratory chain components, promoting increased electron leakage and providing a basis for subsequent mtROS accumulation and oxidative stress amplification (37, 38). A decrease in ΔΨm is an important marker of respiratory chain dysfunction. Once the proton gradient of the inner mitochondrial membrane is compromised, oxidative phosphorylation efficiency decreases, ATP production is reduced, and processes with high energy demands are preferentially affected (39). In COPD-related skeletal muscle, decreased respiratory chain complex activity and reduced oxidative phosphorylation capacity are associated with diminished exercise tolerance and muscle dysfunction, suggesting that mitochondrial energy metabolism abnormalities exist not only in the lungs but also contribute to the extrapulmonary phenotype of the disease (40).

Respiratory chain dysfunction also leads to mtROS accumulation. Excessive mtROS not only promotes lipid peroxidation, protein oxidative modification, and mtDNA damage but can also affect the NAD^+^/NADH balance and multiple metabolic regulatory pathways, driving cells to shift from relatively efficient oxidative phosphorylation to alternative energy acquisition methods, manifesting as glucose and lipid metabolism imbalance and metabolic reprogramming (41, 42). In addition, changes in mitochondrial membrane lipid composition can further affect membrane protein stability and complex assembly efficiency, exacerbating energy metabolism disorders (43). Since different studies employ heterogeneous sample types and detection platforms, indicators such as respiratory chain complex activity, OCR, ATP production, membrane potential, and mtROS should be interpreted in the context of specific experimental designs.

### 2.3 mtDNA damage and mitochondrial stress markers

mtDNA is highly sensitive to oxidative stress, and its oxidative damage, copy number changes, and extracellular release can reflect the state of mitochondrial stress in COPD. Smoking-induced mitochondrial injury can cause a decrease in mitochondrial membrane potential, dynamic imbalance, and activation of DNA damage response, thereby promoting the transfer of mtDNA from mitochondria to the cytoplasm or its release into the extracellular environment (44). Clinical studies have found elevated levels of cell-free mtDNA (cf-mtDNA) in the plasma of COPD patients and the serum of animals in smoke-induced emphysema models, suggesting that cf-mtDNA can serve as a candidate biomarker reflecting the burden of mitochondrial injury (45). *In vitro* experiments further indicate that sublethal concentrations of cigarette smoke extract (CSE) can promote the transfer of mtDNA to the cytoplasm and extracellular space, accompanied by upregulation of DNA damage-related molecules and DNA sensors (46). This section primarily emphasizes the significance of mtDNA as a marker of mitochondrial injury and stress burden. Its role as a DAMP mediating innate immune recognition and inflammatory amplification will be further discussed in Section 3.3.

In addition to cf-mtDNA, various mitochondria-related genes also exhibit dysregulated expression in COPD lung tissue. Existing studies have reported that alterations in the expression of genes such as NDUFS2, BAX, and DLST are associated with mitochondrial respiratory chain function, cell viability, and disease-related immune phenotypes (47). Furthermore, the dysregulated expression of non-coding RNAs, including long non-coding RNAs related to mitochondrial function regulation, suggests that post-transcriptional regulation may be involved in the disruption of mitochondrial homeostasis in COPD (48). Overall, mtDNA damage, elevated cf-mtDNA, and abnormalities in mitochondria-related genes together constitute important features of mitochondrial stress in COPD, providing a molecular basis for subsequent inflammatory recognition and cellular fate imbalance.

It should be pointed out that cf-mtDNA is currently more suitable as a candidate stratification marker rather than a mature diagnostic indicator. Existing studies suggest that plasma or serum cf-mtDNA may be associated with COPD severity, risk of acute exacerbation, smoking exposure, and tissue injury burden. However, differences exist between studies in sample processing, detection platforms, copy number normalization methods, and control population selection (49). Therefore, there is currently no unified cutoff directly applicable for clinical diagnosis or classification, nor are there fully validated sensitivity, specificity, and dynamic range. Since cf-mtDNA can also be elevated in acute lung injury, infection, cardiovascular disease, metabolic diseases, and other inflammatory conditions, its disease specificity is limited. In the future, if cf-mtDNA is to be used for COPD stratification, it would be more suitable to combine it with indicators such as 8-OHdG, MDA, GDF15, FGF21, NLRP3/Gasdermin D (GSDMD), or GPX4, rather than using it as a standalone diagnostic marker (50).

### 2.4 Mitochondrial dysfunction in different tissues and organs

Mitochondrial dysfunction in COPD is not limited to a single cell type but exhibits certain specificity across different structures within the lung and some extrapulmonary tissues. In airway epithelial cells, following continuous exposure to harmful stimuli such as cigarette smoke, mitochondria can show enhanced fission, impaired fusion, and decreased activity, accompanied by increased intracellular oxidative stress and aging-related changes (51). Among these, enhanced DRP1 activation is closely related to excessive mitochondrial fission, and GNPAT can further aggravate mitochondrial functional injury and cell apoptosis by stabilizing DRP1 (52). These changes suggest that airway epithelium is one of the earliest and most persistently affected sites of mitochondrial abnormalities in COPD.

In the alveolar region, particularly in alveolar type II epithelial cells (ATII), decreased expression of MFN2 and OPA1 suggests impaired mitochondrial fusion, thereby promoting mitochondrial injury, cellular senescence, and the formation of a local inflammatory microenvironment (28, 29). Meanwhile, activation of the NEAT1/PINK1-related mitophagy pathway indicates that alveolar cells initiate corresponding quality control responses in response to mitochondrial injury caused by smoke or particulate matter exposure (53). In pulmonary vascular-related cells, mitochondrial injury is more associated with elevated reactive oxygen species, endothelial dysfunction, and pulmonary vascular remodeling, and may participate in the occurrence of complications such as COPD complicated by pulmonary hypertension (54, 55).

Beyond pulmonary structures, skeletal muscle is one of the most representative extrapulmonary tissues affected in COPD. Studies have shown that impaired mitochondrial biogenesis, enhanced fission, and decreased energy metabolism efficiency in the skeletal muscle of COPD patients can together promote muscle atrophy, decreased endurance, and dysfunction (56). Additionally, molecules such as DKK3 are implicated in COPD-related sarcopenia by inducing mitochondrial functional imbalance (57). Therefore, from the intrapulmonary airways and alveoli to extrapulmonary skeletal muscle, mitochondrial abnormalities are not isolated events but may jointly participate in local injury and systemic phenotype formation in COPD. Mitochondrial abnormalities in different tissues do not simply occur in parallel but may correspond to different biomarkers, disease stages, and clinical phenotypes. Overall, mitochondrial abnormalities in COPD are not a single-level structural damage but simultaneously involve morphological disruption, insufficient energy production, mtDNA damage, and functional decline in different tissues. These changes together constitute the mitochondrial basis for subsequent oxidative stress, inflammatory activation, and cellular fate imbalance. Whether mitochondrial injury can be promptly repaired or cleared depends on the integrity of the mitochondrial quality control system (**Table 1**).

**Table 1:** Key quantitative and mechanistic evidence of mitochondrial abnormalities in COPD.

Sample or model	Cell/tissue type	Mitochondrial parameters	Change direction	Main explanation	Reference
COPD patient lung tissue or lung cell samples	Airway epithelial cells, alveolar epithelial cells, pulmonary vascular-related cells	Mitochondrial fragmentation, swelling, cristae structural disruption	Increased morphological abnormalities	Provides morphological evidence of mitochondrial structural damage and impaired function in COPD lung cells.	(25, 26)
COPD lung tissue, alveolar epithelial cells, or smoke exposure models	Lung tissue, alveolar epithelial cells, cell models	Levels of MFN2, OPA1, DRP1, and other mitochondrial dynamics regulators	Decreased MFN2/OPA1, enhanced DRP1-related fission signaling	Supports a shift in mitochondrial dynamics toward insufficient fusion and enhanced fission, associated with mitochondrial fragmentation and decreased membrane potential.	(28–30)
COPD patients or experimental models	Skeletal muscle	Mitochondrial density, morphology, dynamics regulatory indicators.	Decreased mitochondrial density or structural integrity; abnormal fission/fusion-related indicators	Suggests that mitochondrial structural abnormalities are not limited to lung tissue and may also contribute to COPD extrapulmonary phenotypes.	(31, 32)
COPD patients or experimental models	Lung tissue, pulmonary immune cells, or skeletal muscle	Respiratory chain complex activity, ΔΨm, ATP, mtROS.	Decreased respiratory chain function, reduced membrane potential, decreased ATP production, increased mtROS.	Demonstrates that respiratory chain dysfunction and metabolic disturbances are key functional manifestations of mitochondrial abnormalities in COPD.	(35)
COPD lung macrophage-related studies	Lung macrophages	NDUFS2, oxidative phosphorylation, mitochondrial respiratory function, phagocytic function Decreased.	Decreased NDUFS2, accompanied by impaired mitochondrial respiratory function, oxidative phosphorylation capacity, and phagocytic function.	Links complex I abnormalities, lung macrophage dysfunction, and COPD immune homeostasis imbalance.	(36)
COPD patients, smoke exposure models, or related cell models	Lung tissue, lung cells, skeletal muscle	Respiratory chain components, mtDNA damage, NAD+/NADH, membrane lipid composition, OCR, ATP, mtROS.	Decreased respiratory chain function, increased electron leakage, elevated mtROS, reduced ATP production, and metabolic reprogramming.	Supports that smoking and oxidative stress can damage the respiratory chain and mtDNA, driving energy metabolism disorder and amplification of oxidative stress.	(41, 42)
COPD patient plasma, smoke-induced emphysema models, or CSE-stimulated cells	Plasma/serum/cell models	cf-mtDNA, cytosolic mtDNA, DNA damage-related molecules, DNA sensors.	Elevated cf-mtDNA or cytosolic mtDNA; upregulation of DNA damage and sensor-related molecules.	While cf-mtDNA shows promise as a biomarker for mitochondrial damage burden and inflammatory risk, it is not yet established as a routine diagnostic marker.	(44, 45)
COPD lung tissue or transcriptome/noncoding RNA studies	Lung tissue, related lung cells	NDUFS2, BAX, DLST, noncoding RNA, long noncoding RNA	Abnormal expression of mitochondrial-related genes or noncoding RNAs.	Supports the presence of mitochondrial-related transcriptional and post-transcriptional regulatory abnormalities in COPD.	(47, 48)
COPD patient plasma/serum or related clinical studies	Peripheral blood samples	cf-mtDNA and combined candidate biomarkers, such as 8-OHdG, MDA, GDF15, FGF21, NLRP3/GSDMD, GPX4, etc.	cf-mtDNA may be elevated and possibly associated with disease severity, acute exacerbation risk, smoking exposure, or tissue damage burden.	cf-mtDNA has limited specificity; future use is more suitable for research-based stratification in combination with oxidative stress, inflammasome, ferroptosis, or mitochondrial stress indicators.	(49, 50)
COPD airway epithelium or smoke exposure models	Airway epithelial cells, alveolar epithelial cells, pulmonary vascular-related cells.	Mitochondrial fragmentation, swelling, and destruction of ridge structure.	Abnormal increase in morphology.	Supporting the existence of mitochondrial structural damage in lung cells of COPD is an important morphological evidence of impaired mitochondrial function.	(51, 52)
COPD lung tissue, alveolar epithelial cells, or smoke exposure model	lung tissue, alveolar epithelial cells, or smoke exposure models	MFN2, OPA1, DRP1 and mitochondrial dynamics indicators.	decreased MFN2/OPA1 levels and enhanced DRP1-related fission signaling.	Support a shift in mitochondrial dynamics toward impaired fusion and enhanced fission, associated with mitochondrial fragmentation and decreased membrane potential.	(53)
COPD patients or experimental models	• skeletal muscle	Regulatory indicators of mitochondrial density, morphology and dynamics.	Decline in mitochondrial density or structural integrity; Abnormal indicators related to splitting/fusion.	Supports a shift in mitochondrial dynamics toward insufficient fusion and enhanced fission, associated with mitochondrial fragmentation and decreased membrane potential.	(54, 55)
COPD patients or experimental models	Lung tissue, pulmonary immune cells, or skeletal muscle	Respiratory chain complex activity, ΔΨm, ATP, mtROS	Decreased respiratory chain function, reduced membrane potential, decreased ATP production, elevated mtROS.	Supports that respiratory chain dysfunction and energy metabolism disorders are important functional manifestations of mitochondrial abnormalities in COPD.	(56, 57)

### 2.5 Areas of controversy and unresolved questions

Although existing evidence supports the presence of mitochondrial structural and functional abnormalities in COPD, changes in mitochondrial dynamics are not consistently observed across all studies. Some studies report a decrease in fusion-related proteins such as MFN2 and OPA1, accompanied by enhanced DRP1-related fission signaling; however, other studies have observed enhanced fusion responses, no significant changes in dynamic indicators, or inconsistent directions of changes in fusion/fission-related proteins in specific cell types, exposure durations, or disease stages (58, 59). This suggests that mitochondrial dynamics abnormalities are not a fixed “enhanced fission—decreased fusion” pattern, but may be influenced by cell type, injury severity, disease stage, and compensatory status.

These differences may primarily arise from three factors. First, different cell types respond distinctly to smoke, inflammation, and oxidative stress: airway and alveolar epithelial cells are more susceptible to exogenous stimuli, mitochondrial changes in macrophages may primarily reflect metabolic reprogramming and immune dysfunction, while mitochondrial abnormalities in appendicular skeletal muscle more closely resemble long-term energy metabolism disorders (60, 61). Second, disease stage may affect the direction of mitochondrial dynamics: in early stress states, mitochondrial fusion or moderate mitophagy may serve a compensatory role, whereas in long-term injury or advanced disease, enhanced fission, cristae structural damage, and energy depletion may be more prominent (62). Third, tissue source, cell sorting methods, smoke exposure dose and duration, detection platforms, morphological quantification methods, and the distinction between total protein levels and activation status also affect study conclusions (63).

As a marker of mitochondrial damage, cf-mtDNA also has translational limitations. Existing studies suggest it may be associated with COPD staging, acute exacerbation risk, and tissue injury burden, but there is currently a lack of standardized detection protocols, unified units, defined clinical cutoffs, and established benchmarks for sensitivity, specificity, and dynamic range (64). Differences in sample types, DNA extraction methods, qPCR targets, and copy number normalization across studies limit comparability of results. Additionally, cf-mtDNA is not specific to COPD and can be elevated in infections, acute lung injury, atherosclerotic cardiovascular disease, metabolic diseases, and other inflammatory conditions (65, 66). Therefore, cf-mtDNA is currently more suitable as a candidate indicator for mechanistic research and risk stratification, rather than as a standalone diagnostic marker for COPD.

**Figure 1:**
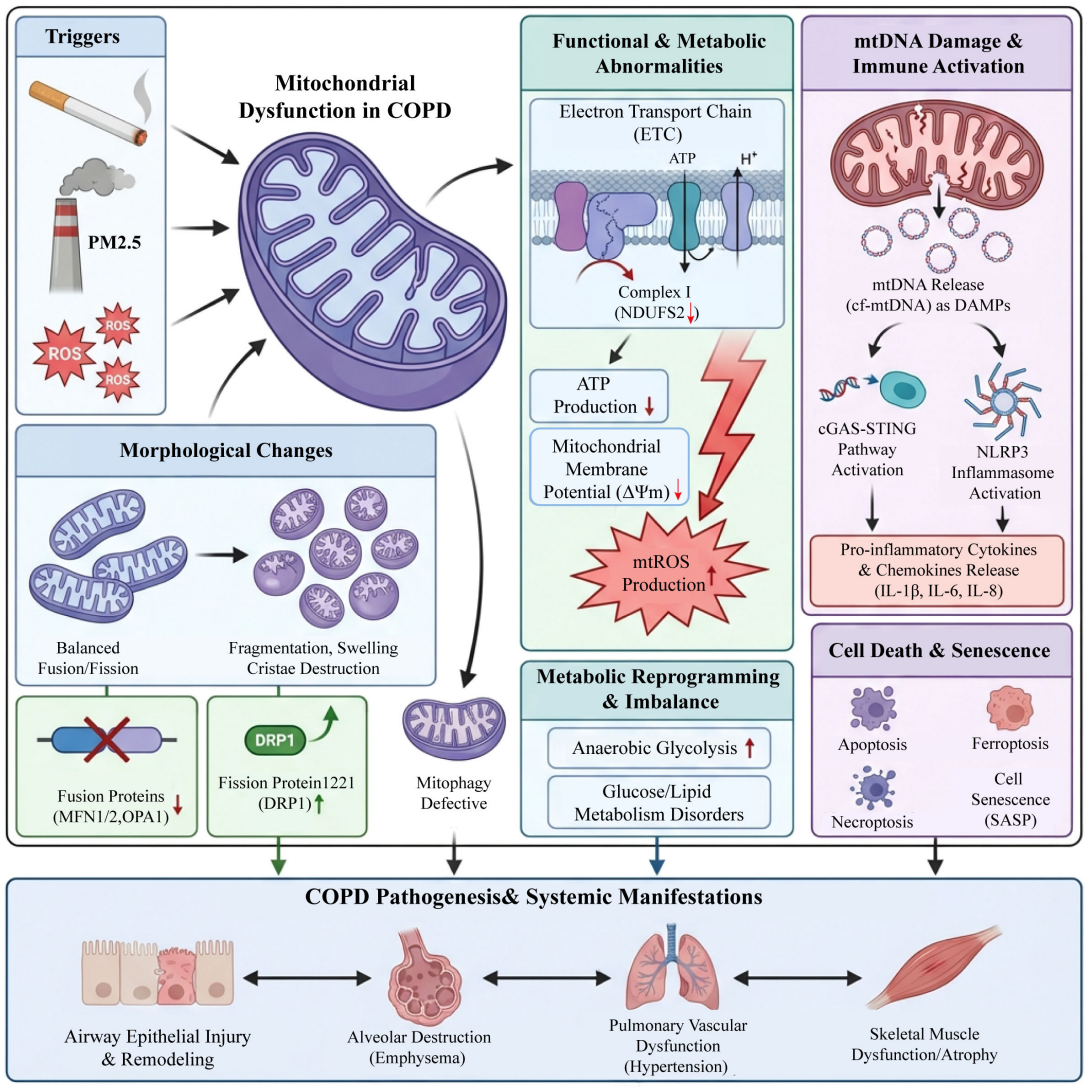
Key pathological chain of mitochondrial dysfunction in COPD. Trigger factors such as cigarette smoking, PM2.5, and oxidative stress can lead to abnormal mitochondrial morphology, respiratory chain impairment, decreased ATP production, reduced membrane potential, and increased mtROS, further affecting tissues including airway epithelia, alveoli, pulmonary vasculature, and skeletal muscle. This figure primarily summarizes the upstream initiation of mitochondrial injury and its main pathological consequences; mitochondrial DAMPs, the oxidative stress amplification loop, and the RCD cross-network are detailed in subsequent figures and corresponding sections. Created in BioRender. Di, X. (2026) https://BioRender.com/ri6u90k.

Future research should prioritize three issues: first, evaluate mitochondrial fusion, fission, mitophagy, respiratory function, and mtDNA release separately in different cell types and COPD stages, avoiding reliance on a single indicator to infer overall mitochondrial status; second, clarify the relationship between mitochondrial indicators and lung function decline, acute exacerbations, emphysema progression, appendicular skeletal muscle function, and prognosis in longitudinal cohorts; third, establish standardized cf-mtDNA detection and reporting procedures, and assess its incremental value when combined with 8-OHdG, MDA, GDF15, FGF21, inflammasome-related molecules, and ferroptosis-related indicators. Only by addressing these issues can mitochondrial abnormalities be further translated from mechanistic observations into reliable bases for COPD phenotyping and intervention selection (**Figure 1**).

## 3 Imbalance of mitochondrial quality control: a key link in the persistence of injury

Under normal conditions, mitochondrial biogenesis, dynamic regulation, and mitophagy collectively maintain the homeostasis of mitochondrial quantity, structure, and function. When damaged mitochondria can be promptly cleared and replenished by newly generated mitochondria, cells may still maintain a compensatory state. However, in the context of persistent exposure and chronic inflammation in COPD, reduced mitochondrial biogenesis, imbalance in fusion/fission, and insufficient or excessive activation of mitophagy transform mitochondrial injury from a transient stress into a chronic cumulative process (19). Among these, the role of mitophagy is highly context-dependent: moderate activation facilitates the clearance of damaged mitochondria, while insufficient clearance or sustained excessive activation may worsen mitochondrial homeostatic imbalance. Thus, MQC imbalance serves as an intermediate link connecting initial mitochondrial injury to subsequent amplification of oxidative stress (67).

### 3.1 Reduced mitochondrial biogenesis

Mitochondrial biogenesis is a fundamental process for maintaining mitochondrial quantity renewal and functional stability. Its core regulatory axis primarily includes peroxisome proliferator-activated receptor gamma coactivator 1-alpha (PGC-1α), mitochondrial transcription factor A (TFAM), and their upstream regulatory molecules (68). PGC-1α is considered a key transcriptional coactivator for mitochondrial biogenesis, cooperating with various nuclear transcription factors to promote mtDNA replication, mitochondrial protein expression, and maintenance of oxidative phosphorylation capacity (69). TFAM directly participates in mtDNA transcription and stabilization, processes that are crucial for maintaining mitochondrial genome integrity (70).

Current studies indicate that the expression of mitochondrial biogenesis-related molecules is generally downregulated in the lung tissue and airway epithelial cells of COPD patients. This includes reduced transcriptional levels of PPARGC1B, PPRC1, and PPARD, alterations that are more pronounced in severe cases (71). Furthermore, decreased PGC-1α expression is often accompanied by reduced mitochondrial number, weakened respiratory chain activity, and diminished antioxidant capacity, suggesting that mitochondrial pathology in COPD involves not only “increased injury” but also “insufficient renewal” (72). This decline in regenerative capacity makes it difficult for cells to replenish functionally intact mitochondria in a timely manner, thereby exacerbating bioenergetic metabolic dysfunction and oxidative stress burden (73).

The SIRT1/AMPK–PGC-1α–TFAM axis is an important regulatory pathway connecting energy sensing, mitochondrial biogenesis, and metabolic adaptation. Tobacco smoke and other harmful exposures can inhibit SIRT1 and AMPK activity, thereby weakening PGC-1α-mediated mitochondrial transcriptional programs, leading to reduced mtDNA replication, mitochondrial protein expression, and oxidative phosphorylation capacity (74–76). This change is not confined to lung tissue. In COPD skeletal muscle, PGC-1α downregulation is also associated with reduced mitochondrial density, decreased oxidative phosphorylation efficiency, and impaired exercise tolerance (77). Overall, reduced mitochondrial biogenesis in COPD essentially reflects a decline in mitochondrial renewal and compensatory capacity.

### 3.2 Autophagy and mitophagy

Within the mitochondrial quality control system, mitophagy is responsible for selectively clearing damaged mitochondria and is a crucial mechanism for maintaining mitochondrial homeostasis (78). In the context of chronic oxidative stress and the inflammatory microenvironment in COPD, the expression of autophagy/mitophagy-related proteins in airway epithelial cells, alveolar cells, and skeletal muscle tissue can be altered, indicating that this quality control program has been dysregulated (79–81).

The most well-studied pathway is the PINK1/Parkin pathway. When mitochondrial membrane potential decreases, PINK1 accumulates on the outer mitochondrial membrane and recruits the E3 ubiquitin ligase Parkin, ubiquitinating damaged mitochondria for subsequent degradation via autophagosomes and lysosomes (82). Moderate mitophagy helps reduce the accumulation of damaged mitochondria, lower mtROS release, and alleviate inflammation and cellular injury (83). Therefore, mitophagy is primarily a protective compensatory response. However, the key issue in COPD is not whether mitophagy is activated, but whether its intensity and duration are appropriate. Insufficient mitophagy leads to the retention of damaged mitochondria, further increasing ROS generation and amplifying membrane potential loss, protein oxidation, and mtDNA damage (84). For example, in COPD-related skeletal muscle dysfunction, CSE can downregulate Parkin expression, block the clearance of damaged mitochondria, promote ROS accumulation and MuRF-1-mediated protein degradation, ultimately driving muscle atrophy (85). Conversely, under persistent mitochondrial injury, sustained excessive activation of mitophagy may also lead to excessive loss of mitochondrial quantity, further reducing ATP production and exacerbating energy crisis (86).

The transition of mitophagy from a protective response to a pathological process may depend on injury intensity and duration, cellular energy status, redox state, and the compensatory capacity of other quality control pathways (87). Transient or moderate injury is typically recognized through PINK1/Parkin, BNIP3/NIX, FUNDC1, or other receptor/adaptor pathways, and can be cleared when autophagosome formation and lysosomal degradation function are relatively intact, thereby reducing mtROS burden, decreasing DAMPs release, and maintaining cellular homeostasis (88, 89). Conversely, if smoke exposure, inflammatory stimulation, or oxidative stress persists, the scope of mitochondrial injury expands, ATP/AMP ratio decreases, and energy-sensing signals such as AMPK, mTOR, and ULK1 become imbalanced, potentially exposing cells to both insufficient clearance of damaged mitochondria and excessive loss of functional mitochondria (90). When mitochondrial biogenesis is insufficient to compensate for mitochondrial clearance, or lysosomal degradation capacity is limited, mitophagy may shift from protective quality control to a pathological process promoting energy depletion, inflammation amplification, and cell death (91).

In airway smooth muscle cells, excessive mitophagy induced by ERK1/2 activation is believed to be associated with cellular structural damage (92). In addition to PINK1/Parkin, pathways such as MAPK15-ULK1, PI3K/Akt/mTOR, and SIRT1 also participate in mitophagy regulation, collectively influencing its intensity, duration, and cell-type-specific responses (93, 94). Therefore, abnormal mitophagy in COPD should be understood as a dynamic regulatory imbalance rather than simple enhancement or suppression. From a therapeutic perspective, the “mitophagy paradox” suggests that simply enhancing or inhibiting mitophagy may be overly simplistic. A more rational strategy should involve modulation based on disease stage and cellular state: in early or reversible injury stages, promoting selective clearance of damaged mitochondria while supporting PGC-1α-related mitochondrial biogenesis; in stages of persistent injury or significant energy depletion, avoiding excessive clearance of functional mitochondria while simultaneously improving lysosomal degradation capacity, redox homeostasis, and energy metabolism (95). In the future, stratification based on indicators such as mtROS, membrane potential, PINK1/Parkin activity, LC3-II/I, p62, PGC-1α, and ATP/AMP ratio may help determine whether mitophagy is in a protective or pathological state (**Table 2**).

**Table 2:** The mitophagy paradox in COPD: when protective quality control becomes pathological amplification.

Determinant	Protective mitophagy	Pathological mitophagy	Interpretation in COPD	Reference
Intensity and duration of mitochondrial damage	When mitochondrial damage is transient or moderate, damaged mitochondria can be selectively recognized and cleared	Long-term smoke exposure, persistent inflammation, or high oxidative stress cause mitochondrial damage to exceed compensatory capacity	The key issue of mitophagy in COPD is not whether it is activated, but whether its intensity and duration are appropriate.	(79–81)
PINK1/Parkin-dependent clearance	PINK1 accumulates on the outer mitochondrial membrane and recruits Parkin, promoting ubiquitination and autophagic clearance of damaged mitochondria.	When Parkin expression decreases or the pathway is blocked, clearance of damaged mitochondria is insufficient, leading to ROS accumulation and cellular damage.	In CSE-related appendicular skeletal muscle dysfunction, Parkin downregulation can block the clearance of damaged mitochondria and promote MuRF-1-mediated protein degradation and muscle atrophy.	(83–85)
Cellular energy state	The ATP/AMP ratio is relatively maintained; after mitochondrial clearance, it can be supplemented by PGC-1α-related mitochondrial biogenesis.	ATP decreases, energy-sensing signals such as AMPK, mTOR, and ULK1 become imbalanced; the rate of mitochondrial clearance exceeds the capacity for new biogenesis.	When energy reserves are insufficient or mitochondrial biogenesis is limited, sustained mitophagy may further exacerbate the energy crisis.	(90, 91)
Redox status and mtROS burden	Moderate mitophagy can reduce the accumulation of damaged mitochondria, decreasing mtROS release, oxidative damage, and DAMPs leakage.	When mitophagy is insufficient, mtROS continues to rise, membrane potential declines, protein oxidation, mtDNA damage, and inflammatory amplification increase.	The mtROS burden can serve as an important functional readout for determining whether mitophagy remains protective.	(88, 89)
Availability of mitophagy receptors/adaptors	Moderate activation of pathways such as PINK1/Parkin, BNIP3/NIX, and FUNDC1 facilitates selective clearance of damaged mitochondria	Insufficient receptors/adaptors lead to clearance failure; sustained or excessive activation may cause excessive mitochondrial loss	Different cell types in COPD may rely on different mitophagy pathways; therefore, mitophagy should not be simply defined as enhanced or inhibited.	(93, 94)
Cross-talk with other mitochondrial quality control pathways	Mitophagy is coordinated with mitochondrial biogenesis, fusion/fission regulation, and lysosomal degradation processes.	When biogenesis is insufficient, fusion/fission is imbalanced, or lysosomal degradation is limited, the overall mitochondrial quality control network becomes dysfunctional.	Mitophagy abnormalities in COPD should be understood as a dynamic quality control imbalance, rather than a single pathway abnormality.	(95)
Cell-type-specific response	In airway epithelial cells, alveolar cells, or appendicular skeletal muscle, moderate mitophagy helps maintain cellular homeostasis.	In contexts such as airway smooth muscle cells, ERK1/2 activation-induced excessive mitophagy may be associated with cellular structural damage.	The threshold, compensatory capacity, and pathological consequences of mitophagy may differ across cell types	(92)
Biological consequence	Clears damaged mitochondria, reduces mtROS, decreases DAMPs release, maintains cellular energy and homeostasis	Loss of functional mitochondria, decreased ATP production, inflammatory amplification, enhanced protein degradation, and increased cell death	The shift of mitophagy from a protective response to a pathological process may be a critical mechanism in COPD progression and the formation of extrapulmonary phenotypes.	(86)
Therapeutic implication	In the early or reversible injury stage, it can promote selective clearance of damaged mitochondria while supporting PGC-1α-related mitochondrial renewal	In the stage of sustained injury or energy depletion, non-selective or long-term excessive activation of mitophagy should be avoided.	Therapeutically, stratification and regulation should be guided by biomarkers including mtROS, membrane potential, PINK1/Parkin, LC3-II/I, p62, PGC-1α, and the ATP/AMP ratio.	(95)

### 3.3 Mitochondrial DAMP release: a link between MQC dysfunction and inflammatory initiation

Mitochondrial injury not only signifies energy metabolism failure but may also lead to the exposure of endogenous danger signals. Due to the bacterial ancestry of mitochondria, once their components are released from compromised organelles, they can be recognized by the host immune system as DAMPs, thereby providing upstream stimulation for subsequent inflammatory responses (96). In COPD, mitochondrial DAMPs primarily include mtDNA, cardiolipin, ATP, and certain mitochondrial protein components (97, 98). Rather than simply understanding DAMP release as passive leakage following mitochondrial injury, it is more appropriate to view it as a consequence of mitochondrial quality control failure.

MQC abnormalities can promote DAMP exposure through multiple pathways. First, insufficient mitophagy leads to the persistent retention of mitochondria with decreased membrane potential and severe oxidative damage, thereby heightening the risk of mtDNA oxidation, fragmentation, and release into the cytoplasm or extracellular space. Second, imbalances in mitochondrial fusion/fission can disrupt mitochondrial network integrity, making it more difficult to effectively isolate and eliminate damaged mitochondria, further increasing the risk of DAMP leakage. Third, decreased mitochondrial membrane integrity or opening of the permeability transition pore can facilitate the escape of mtDNA, ATP, and other mitochondrial contents; meanwhile, abnormal exposure of cardiolipin from the inner membrane to the outer membrane indicates remodeling of the mitochondrial membrane structure and may serve as an important signal for inflammatory recognition (99–102). Therefore, mitochondrial DAMP release is not a single pathway event but rather the result of the combined effects of mitochondrial membrane injury, mitophagy imbalance, dynamic abnormalities, and oxidative stress.

**Figure 2:**
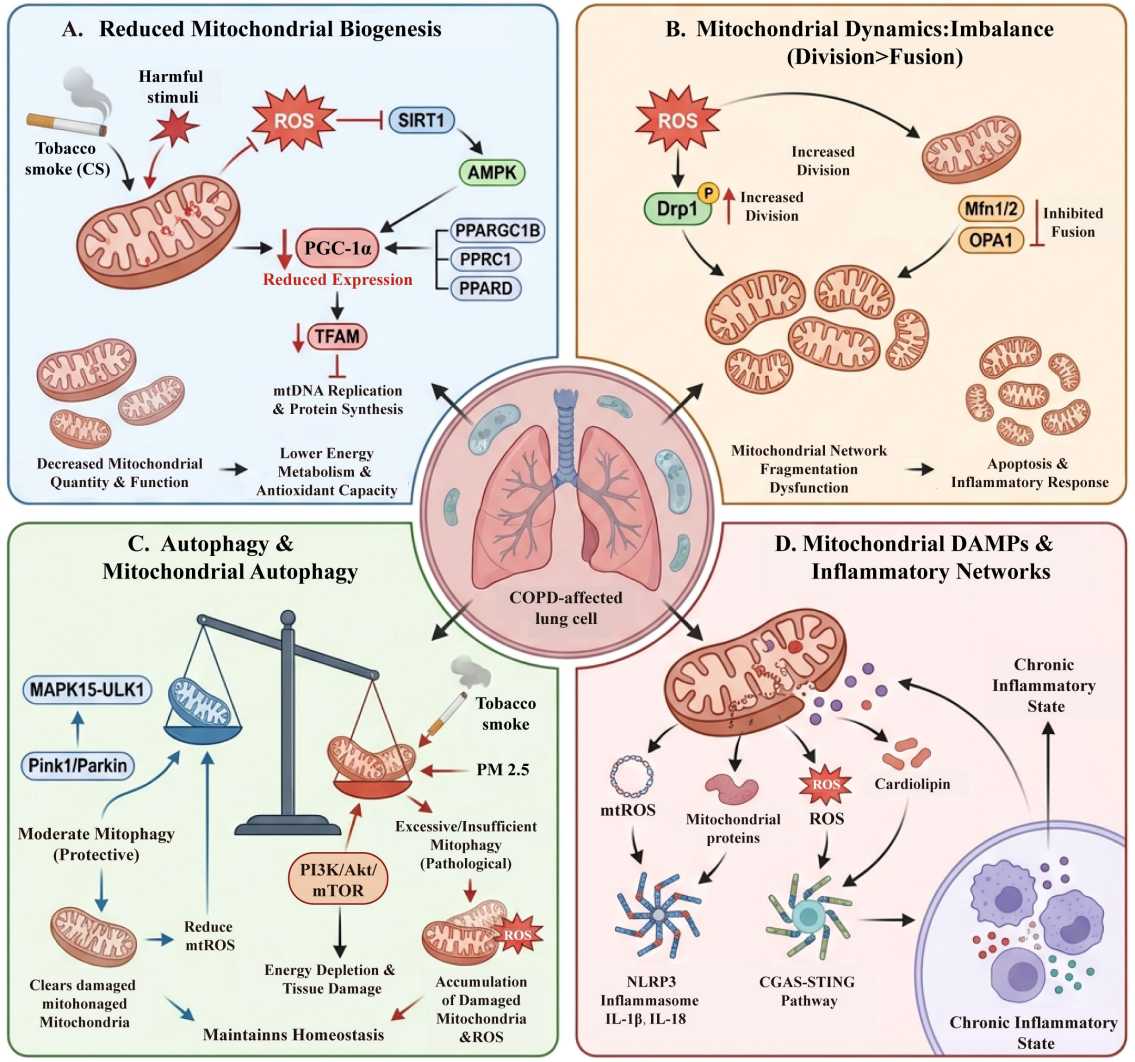
Mitochondrial dysfunction-driven oxidative stress and inflammation in COPD. **(A) **Tobacco smoke and harmful stimuli suppress the SIRT1/AMPK/PGC-1α/TFAM axis through ROS, reducing mitochondrial biogenesis, energy metabolism, and antioxidant capacity. **(B)** ROS promotes Drp1-mediated fission and inhibits Mfn1/2- and OPA1-dependent fusion, causing mitochondrial fragmentation, dysfunction, and inflammatory activation. **(C)** Moderate mitophagy clears damaged mitochondria and maintains homeostasis, whereas excessive or insufficient mitophagy aggravates mtROS accumulation and tissue injury. **(D)** Damaged mitochondria release mtROS, mtDNA, mitochondrial proteins, and cardiolipin as DAMPs, activating the NLRP3 inflammasome and cGAS-STING pathway and sustaining chronic inflammation in COPD. (Created in BioRender. Di, X. (2026) https://BioRender.com/toh1n17).

The impact of MQC imbalance on inflammation depends not only on whether DAMPs are generated but also on whether damaged mitochondria can be promptly cleared and whether new mitochondria can be replenished. Insufficient mitochondrial biogenesis limits the renewal of functional mitochondria; inadequate mitophagy leads to the accumulation of damaged mitochondria, whereas excessive mitophagy may cause loss of functional mitochondria and energy crisis (103, 104). All these changes can increase the likelihood of DAMP release and persistent inflammation. Overall, this section primarily emphasizes how MQC imbalance controls the production and release of mitochondrial DAMPs; how these DAMPs further activate inflammasomes and induce pyroptosis will be discussed in detail in Section 5.2 (**Figure 2**).

## 4 Oxidative stress amplification loop: from mitochondrial damage to persistent inflammation

Building upon the aforementioned mitochondrial damage and MQC imbalance, oxidative stress can be understood as a core amplification mechanism driving the continuous expansion of the COPD pathological network. Exogenous ROS, ROS from inflammatory cells, and mtROS converge, sustaining COPD tissues in a state of high oxidative load for a prolonged period. More importantly, mitochondria are both targets of ROS attack and significant sources of sustained ROS generation. Therefore, this chapter focuses on how ROS sources, mtROS-mediated mitochondrial damage, specific positive feedback loops, and antioxidant defense imbalance collectively maintain the oxidative stress state in COPD.

### 4.1 Sources of ROS in COPD

Oxidative stress is a core pathological link in the occurrence and progression of COPD, and the continuous accumulation of ROS is the primary driver of this process. ROS in COPD mainly originate from exogenous exposure and endogenous generation, which interact to jointly promote airway damage, persistent inflammation, and lung tissue remodeling (105, 106). Exogenous ROS are primarily related to environmental factors such as smoking and air pollution (107). Cigarette smoke is rich in free radicals and oxidizing chemicals, which can directly damage the airway epithelium and activate alveolar macrophages and other inflammatory cells, thereby amplifying local oxidative stress and inflammatory responses (108). In addition to cigarette smoke, fine particulate matter such as PM2.5 can also deposit in the respiratory tract, causing a local increase in ROS and promoting airway inflammation, epithelial barrier damage, and an increased risk of acute exacerbations (109).

Endogenous ROS mainly originate from oxidase systems and mitochondria. The expression and activity of the NADPH oxidase (NOX) family can be elevated in COPD lung tissue, with subtypes such as NOX1, NOX2, NOX4, and NOX5 participating in ROS generation and activating inflammation-related pathways like NF-κB and COX-2, thereby promoting the maintenance of airway inflammation (110). Concurrently, damage to the mitochondrial respiratory chain increases electron leakage, leading to enhanced generation of mtROS. Unlike exogenous ROS, mtROS are not only a result of oxidative damage but also a significant driving factor for the continuous deterioration of mitochondrial function (111). Furthermore, COPD patients often experience depletion of glutathione (GSH) and a decline in other antioxidant reserves, making it difficult to promptly clear excess ROS (112). Elevated activity of certain endogenous enzymes, such as monoamine oxidase B, may also participate in the amplification of ROS in smoking-related lung injury (113). Overall, ROS in COPD are driven by exogenous oxidative exposure, NOX system activation, and mitochondrial respiratory chain abnormalities, forming a mutually amplifying pathological network during the process of continuous damage.

### 4.2 Damage of mtROS to mitochondria and cellular components

The “vicious cycle of oxidative stress” primarily refers to the reciprocal amplification among mtROS accumulation, aggravated mitochondrial damage, decreased antioxidant defense, and activation of inflammation/RCD. Moderate levels of mtROS can participate in cellular signaling regulation, but under the context of sustained exposure and chronic inflammation in COPD, excessive mtROS can transform from signaling molecules into damaging factors, further disrupting mitochondrial structural integrity and cellular homeostasis (114). First, mtROS can attack mitochondrial membrane lipids, especially those rich in unsaturated fatty acids, leading to lipid peroxidation, altered membrane fluidity, and decreased membrane potential (115). Following structural damage to the membrane, maintaining the proton gradient across the inner mitochondrial membrane becomes compromised; consequently, oxidative phosphorylation efficiency declines, ultimately exacerbating ATP synthesis deficits and precipitating an energy crisis (116, 117).

Second, mtROS can cause oxidative modification of mtDNA, changes in copy number, and transcriptional abnormalities, impairing respiratory chain protein synthesis and further reducing electron transfer efficiency (118, 119). Third, mtROS can oxidize mitochondrial proteins, leading to conformational changes and functional inactivation of respiratory chain complex proteins, membrane transport proteins, and quality control-related proteins, thereby affecting complex assembly, membrane permeability, and mitochondrial turnover (120, 121). Therefore, mtROS amplification in COPD is not a vague “vicious cycle” but is maintained by several specific positive feedback loops. First, mtROS can damage mtDNA, leading to impaired expression and assembly of mitochondrially-encoded respiratory chain complex subunits, and decreased electron transfer efficiency further increases electron leakage, generating more mtROS (122). Second, mtROS can induce peroxidation of cardiolipin and other mitochondrial membrane lipids, reducing membrane potential and disrupting the stability of respiratory chain complexes, and complex assembly defects further exacerbate electron leakage and mtROS generation (123). Third, sustained mtROS load can inhibit PGC-1α-related mitochondrial biogenesis, making it difficult for damaged mitochondria to be promptly replaced by newly functional ones, leading to the accumulation of damaged mitochondria and continuous mtROS release (124). Fourth, mtROS can also affect the Keap1–Nrf2 antioxidant axis; acute or moderate oxidative stress can activate Nrf2 through Keap1 cysteine modification, while long-term high-intensity oxidative stress may lead to Nrf2 response exhaustion or inhibition, decreasing ROS clearance capacity and further strengthening the oxidative load (125). These loops collectively constitute the molecular basis for the persistence of mtROS and self-amplification of mitochondrial damage in COPD.

When mitochondrial protein folding stress persists, the mitochondrial unfolded protein response (UPRmt) may be triggered to enhance the expression of mitochondrial chaperones, proteases, and antioxidant-related genes, thereby helping to restore mitochondrial protein homeostasis (126). UPRmt differs from the classical endoplasmic reticulum stress response (ER stress); the latter primarily senses the accumulation of misfolded proteins in the ER lumen and regulates protein folding and secretion load through pathways like PERK, IRE1, and ATF6. In contrast, UPRmt emphasizes the retrograde stress signal sent from the mitochondria to the nucleus upon impaired mitochondrial protein homeostasis, participating in the regulation of mitochondrial chaperones, protein degradation, metabolic adaptation, and antioxidant responses (127, 128). In the context of COPD, UPRmt can be understood as a compensatory protective mechanism following mitochondrial protein oxidative damage; however, if the mtROS load remains excessively high or the mitochondrial quality control system is imbalanced, this compensation may be insufficient to restore homeostasis, instead indicating that the mitochondria have entered a state of sustained stress (129). Finally, mtROS are also important upstream signals for inflammation and cell death pathways. Continuously elevated mtROS can promote inflammasome activation and drive various cell death modalities such as apoptosis, necroptosis, and ferroptosis (130). This mtROS–mitochondrial damage positive feedback is a crucial foundation for the persistence of oxidative stress in COPD.

### 4.3 Imbalance of antioxidant defense pathways

Oxidative stress in COPD not only stems from increased ROS generation but is also closely related to a decline in antioxidant defense capacity. Among these, the nuclear factor erythroid 2-related factor 2 (Nrf2) signaling pathway is one of the core regulatory systems for maintaining cellular antioxidant homeostasis (27, 131). Nrf2 can induce the expression of various antioxidant and detoxification-related genes, including HO-1, SOD, GCLC, and GSH metabolism-related molecules, thereby helping cells resist oxidative damage (132, 133). In COPD, endogenous antioxidant defense impairment, represented by Nrf2 pathway inhibition and Keap1-Nrf2 regulatory imbalance, is an important basis for the persistence of oxidative stress (134). This pathway also represents a promising therapeutic target for restoring antioxidant defenses in future ferroptosis-related interventions. It should be noted that Nrf2’s response to oxidative stress is not unidirectional or linear. Acute or moderate ROS exposure typically promotes Nrf2 stabilization and nuclear translocation through Keap1 cysteine modification, thereby inducing antioxidant gene expression, constituting an adaptive protective response (135). However, in the setting of long-term, high-intensity oxidative stress in COPD, this protective response may be progressively depleted or remodeled, manifesting as insufficient Nrf2 nuclear translocation, decreased downstream antioxidant enzyme expression, enhanced proteasomal degradation, transcriptional repression, or epigenetic dysregulation (109, 136). In other words, the Nrf2 pathway may undergo a biphasic change of “early activation—chronic exhaustion/inhibition,” rather than simply being continuously enhanced or continuously decreased.

Tobacco smoke and other harmful exposures can continuously induce ROS generation, leading to oxidative damage to mitochondrial DNA, membrane lipids, and proteins; simultaneously, hindered Nrf2 nuclear translocation and decreased expression of its downstream antioxidant enzymes further weaken the cell’s ability to clear the oxidative load (137, 138). This bidirectional imbalance of “ROS increase—defense decrease” keeps COPD airway and alveolar tissues in a state of oxidative stress for a long time. Under normal conditions, Keap1 and Nrf2 jointly maintain the dynamic balance of the antioxidant response; while under the context of sustained COPD-related stress, the Keap1-Nrf2 regulatory relationship becomes imbalanced, which can manifest as insufficient Nrf2 activation, restricted nuclear translocation, and difficulty in effectively initiating the antioxidant transcriptional program (134). This biphasic characteristic also helps explain the limited clinical efficacy of some antioxidant treatments. If the disease is in an early stage or the endogenous Nrf2 response is still recoverable, Nrf2 activators may help enhance antioxidant defense; but in advanced stages or under long-term high oxidative load, if the Nrf2 pathway is already in a state of exhaustion, imbalance, or epigenetic suppression, simply activating Nrf2 may be difficult to reverse systemic oxidative damage, potentially yielding limited or unstable efficacy (139, 140). Therefore, interventions targeting Nrf2 require stratification based on disease stage, oxidative stress load, and endogenous antioxidant response status.

Insufficient antioxidant defense results not only in the accumulation of oxidative damage but also disrupts mitochondrial homeostasis (141). Inhibition of the Nrf2 pathway can exacerbate mitochondrial damage, a decline in membrane potential, and energy metabolism disorders, while worsening mitochondrial function leads to more mtROS generation, further amplifying the oxidative stress load (142). Existing studies also suggest that oxidative stress-related genes such as HSPA1A, GCLC, and IL-1β exhibit abnormal expression in COPD, indicating that antioxidant defense imbalance involves multi-level transcriptional regulatory changes (143, 144). Therefore, Keap1–Nrf2 imbalance in COPD not only weakens antioxidant defense but also further aggravates mitochondrial damage and chronic oxidative stress, providing a basis for the spread of oxidative stress to extrapulmonary tissues.

### 4.4 Systemic oxidative stress and multi-organ damage

Oxidative stress in COPD is not confined to the local lung environment. As the disease progresses, the persistent oxidative and inflammatory load can spread to extrapulmonary tissues via the circulatory system, contributing to systemic manifestations such as skeletal muscle dysfunction and cardiovascular comorbidities (145). Rather than generalizing this process as “multi-organ damage,” it is more specifically understood as: COPD-related systemic oxidative stress primarily affects tissues highly sensitive to energy metabolism and inflammatory signals.

Skeletal muscle represents one of the most prominently affected extrapulmonary tissues. COPD patients often experience decreased exercise tolerance, muscle weakness, and sarcopenia, which are closely related to impaired skeletal muscle mitochondrial function and elevated oxidative stress (146). Under sustained oxidative load, the efficiency of the skeletal muscle mitochondrial respiratory chain declines, leading to insufficient ATP production and increased mtROS accumulation, which further promotes protein degradation and impairs muscle fiber function (147). Therefore, skeletal muscle abnormalities are not merely secondary to reduced activity but may be a direct consequence of COPD systemic oxidative stress and metabolic imbalance.

The cardiovascular system is another important target organ for COPD comorbidities. Long-term oxidative stress and chronic low-grade inflammation may be involved in endothelial dysfunction, abnormal vascular reactivity, and the progression of atherosclerosis, and are considered potential bridging mechanisms for the increased risk of coronary heart disease, heart failure, and other cardiovascular events in COPD patients (148, 149). Although existing evidence supports an association between oxidative stress and cardiovascular comorbidities, the magnitude of this effect and the underlying causal pathways remain confounded by multiple factors, including smoking, age, metabolic status, and comorbidity burden (150, 151). Therefore, a more cautious statement would be: persistent systemic oxidative stress may participate in the process of pulmonary chronic inflammation extending to vascular lesions and cardiovascular risk, rather than solely determining these damage outcomes.

**Figure 3:**
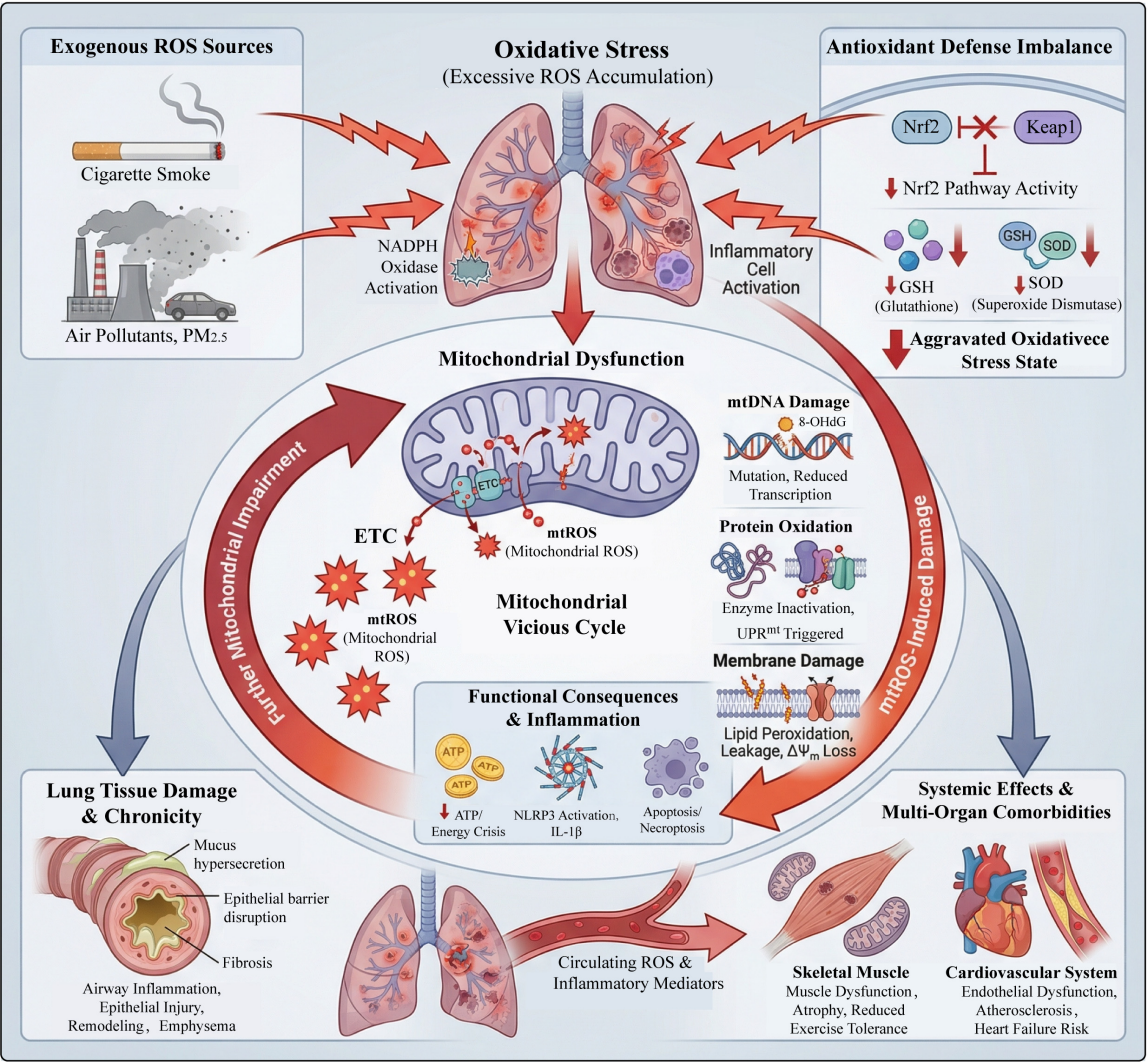
Vicious cycle of mutual amplification between oxidative stress and mitochondrial dysfunction in COPD. Exogenous ROS sources such as smoking and air pollution, combined with inflammatory cell activation and NADPH oxidase upregulation, collectively lead to excessive ROS accumulation in the lungs; concurrently, inhibition of Nrf2 antioxidant defense and diminished levels of GSH and SOD further aggravate oxidative stress. At the mitochondrial level, excessive mtROS can cause mtDNA damage, protein oxidation, and membrane lipid peroxidation, leading to impairment of the electron transport chain, loss of membrane potential, ATP depletion, and inflammasome activation, thereby establishing a self-perpetuating cycle characterized by “mtROS-induced damage leading to further deterioration of mitochondrial function.” Ultimately, this process not only drives pulmonary pathologies such as airway inflammation, mucus hypersecretion, epithelial barrier disruption, and emphysema but can also extend to skeletal muscle and the cardiovascular system via circulating ROS and inflammatory mediators, promoting systemic complications of COPD. Created in BioRender. Di, X. (2026) https://BioRender.com/t8so4tv.

From a clinical assessment perspective, peripheral oxidative stress markers provide clues for understanding the systemic oxidative state in COPD. Studies have shown that oxidative damage indicators such as 8-hydroxy-2’-deoxyguanosine (8-OHdG) and malondialdehyde (MDA) can be elevated in COPD patients, especially during acute exacerbations, and are associated with disease severity, lung function decline, and prognostic risk (152, 153). Additionally, mitokines such as GDF15 and FGF21 have been proposed to reflect mitochondrial stress and systemic metabolic abnormalities (154). GDF15 is a stress-responsive cytokine induced by mitochondrial stress, inflammatory stimuli, and tissue damage, involved in energy intake, metabolic adaptation, and inflammatory regulation; in COPD, its elevation may indicate systemic mitochondrial stress, increased inflammatory load, and worse clinical outcomes (155). FGF21 is a metabolic regulator related to energy metabolism, lipid oxidation, and insulin sensitivity, which can be induced under conditions of mitochondrial dysfunction and nutritional/oxidative stress (156); in COPD, elevated FGF21 may reflect both mitochondrial damage and represent a compensatory response to maintain metabolic homeostasis (157). Although these mitokines have not yet entered routine clinical classification systems, they are helpful for understanding COPD extrapulmonary phenotypes from the perspective of systemic metabolism and mitochondrial stress. Thus, oxidative stress in COPD not only reflects the burden of local and systemic damage but also establishes a common pathological foundation for the subsequent activation of various regulated cell death modalities via mtROS accumulation, inhibition of antioxidant defenses, and DAMP release (**Figure 3**).

## 5 Regulated cell death: the downstream execution phase of the mitochondrial stress network

In COPD, various regulated cell death modalities do not occur in isolation but are activated against a common background of mitochondrial stress and oxidative damage (84). Changes in mitochondrial membrane permeability, elevated mtROS, release of DAMPs, and disruption of iron homeostasis can respectively drive RCD programs such as apoptosis, necroptosis, pyroptosis, and ferroptosis (158). It should be emphasized that the RCD network in COPD is not a static parallel pathway but exhibits distinct cell- and stage-specificity: airway epithelial cells are more susceptible to exogenous exposure and barrier damage, macrophages are more prominent in amplifying inflammation, airway smooth muscle cells are primarily involved in airway remodeling, and skeletal muscle reflects extrapulmonary energy metabolism disorders (159, 160). Therefore, this chapter will discuss different RCD modalities by integrating “mechanism type—cell localization—stage characteristics” to reflect the dynamic and heterogeneous nature of the COPD cell death network.

### 5.1 Mitochondria-mediated apoptosis and necroptosis

In the cell death network associated with mitochondrial dysfunction, apoptosis and necroptosis are not independent of each other but share upstream stress signals at multiple nodes and can undergo switching or crosstalk under certain conditions (161). Persistent tobacco smoke exposure, oxidative stress, and mitochondrial damage provide a common activation background for these two cell death modalities. In mitochondria-mediated intrinsic apoptosis, mitochondrial outer membrane permeabilization is a key step. After Bcl-2 family protein imbalance, Bax and Bak aggregate on the mitochondrial outer membrane, promoting the release of cytochrome c into the cytoplasm, which in turn facilitates apoptosome formation and activates the caspase-9/caspase-3 cascade (162, 163). The loss of mitochondrial membrane potential serves as both a hallmark of mitochondrial damage and a mechanism that further amplifies apoptotic signals (164). From a cell type perspective, apoptosis is often associated with the loss of airway epithelial cells and alveolar epithelial cells, repair disorders, and alveolar structural degeneration; in skeletal muscle, persistent energy deficits and oxidative stress may also enhance susceptibility to apoptosis, contributing to muscle atrophy and decreased exercise tolerance (165).

Necroptosis is more characterized by membrane rupture, leakage of cellular contents, and secondary enhancement of inflammation. Elevated mtROS can promote the activation of RIPK3 and its substrate MLKL, which oligomerizes and inserts into the cell membrane to form pores, ultimately leading to loss of membrane integrity (166). Under conditions of significant mitochondrial damage, the RIPK3/MLKL signaling may also cross-talk with Bax/Bak, Drp1, and changes in membrane permeability, causing cell death that was originally biased towards apoptosis to shift towards more inflammatory necroptosis (167, 168). This switch is not solely determined by an ill-defined “stress threshold” but may be regulated by several molecular switches. For example, when caspase-8 activity is inhibited or absent, RIPK1/RIPK3 signaling is more likely to shift from apoptotic regulation to necroptosis execution; when RIPK1 is deubiquitinated by enzymes such as CYLD, it can also promote the formation of RIPK1-dependent death complexes (169); additionally, severe ATP depletion can weaken the energy-demanding apoptotic program, making cells more inclined to enter inflammatory necrotic-like death (170). This switch is typically more likely to occur in moderate-to-late stage COPD and acute exacerbation environments with higher injury burden, failure of inflammatory clearance, or obvious cellular energy crisis.

The convergence of these two pathways is evident in three key aspects: first, altered membrane permeability serves as a common foundation; second, mtROS amplifies both apoptotic signals and RIPK3/MLKL-mediated necroptosis; and third, calcium homeostasis imbalance and mPTP opening exacerbate membrane potential loss and energy failure, driving the transition from relatively “quiet” apoptosis to highly inflammatory necroptosis. Therefore, in the context of persistent damage in COPD, apoptosis and necroptosis are better understood as two death programs that share a background of mitochondrial damage and can be coupled with each other.

### 5.2 Mitochondrial DAMPs and pyroptosis

Among inflammatory cell deaths associated with mitochondrial dysfunction, pyroptosis is one of the important mechanisms for inflammatory amplification and tissue damage in COPD. MQC imbalance can promote the release of mitochondrial DAMPs such as mtDNA, cardiolipin, and ATP, and these danger signals subsequently trigger inflammasome activation and pyroptosis execution programs (67). Overall, pyroptosis in COPD proceeds via the cascade of “mitochondrial damage/DAMPs release—inflammasome sensor activation—caspase-1 activation—GSDMD cleavage,” driving persistent local inflammation and aggravated tissue damage.

During mitochondrial dysfunction, mtDNA leakage serves as a pivotal event linking organelle damage to inflammatory sensing. After an increase in mitochondrial membrane permeability or mPTP opening, mtDNA can enter the cytoplasm and be recognized by DNA sensing systems such as cGAS–STING, thereby inducing type I interferon and inflammation-related signals, forming a cross-amplification loop with inflammasome activation (171–173). Meanwhile, abnormal exposure of cardiolipin and elevated mtROS can provide stimulatory conditions for NLRP3 inflammasome assembly; ATP release can further promote inflammasome activation through ion efflux mediated by the P2X7 receptor (174). Therefore, mitochondrial DAMPs are important upstream signals that convert mitochondrial damage into inflammatory cell death.

After NLRP3 inflammasome activation, it promotes the activation of caspase-1, which in turn mediates the mature release of IL-1β and IL-18 and cleaves GSDMD. The N-terminal fragment of GSDMD inserts into the cell membrane to form pores, leading to cell swelling, disruption of membrane integrity, and pyroptosis (175, 176). In COPD, the significance of pyroptosis lies not only in the cell death itself but also in the release of pro-inflammatory cytokines that can promote the recruitment of inflammatory cells and aggravate airway and alveolar damage.

Regarding cell specificity, pyroptosis is predominantly observed in alveolar macrophages and airway epithelial cells, contributing to inflammatory cell infiltration. Pyroptosis of macrophages can promote the release of IL-1β and IL-18, driving neutrophil recruitment and maintenance of local inflammation; pyroptosis of airway epithelial cells may disrupt the epithelial barrier, making it easier for exogenous stimuli, pathogens, and inflammatory mediators to enter the airway microenvironment. Additionally, GSDMD cleavage fragments can affect mitochondrial membrane stability and interact with cardiolipin, leading to decreased membrane potential and persistently elevated ROS (177, 178). However, this mechanism is still mainly derived from specific experimental studies, and direct validation in COPD models still needs further strengthening. Overall, pyroptosis may be more prominent during the inflammatory amplification phase, acute exacerbation phase, or the chronic inflammatory stage of moderate-to-late disease, serving as an important node connecting mitochondrial DAMPs release, innate immune activation, and persistent inflammation in COPD (179).

### 5.3 Mitochondrial iron homeostasis and ferroptosis

Ferroptosis has been a subject of intense investigation in COPD research in recent years, but summarizing it merely as “increased lipid peroxidation leading to ferroptosis” is insufficient to reflect its mitochondrial characteristics (160). Mitochondria are not only victims of oxidative damage but also important sites for iron homeostasis regulation and amplification of lipid peroxidation (180). The onset of ferroptosis in COPD is closely linked to imbalances in mitochondrial iron metabolism.

Mitochondrial iron homeostasis depends on fine regulation of iron import, utilization, and storage. The Mitoferrin family of proteins is responsible for importing iron into mitochondria, providing substrates for the synthesis of iron-sulfur clusters and heme, which in turn participate in respiratory chain complex assembly and maintenance of mitochondrial metabolism (181). Once mitoferrin expression or function is abnormal, the mitochondrial iron load can change, affecting electron transport efficiency and promoting ROS generation (182). In the chronic oxidative stress environment of COPD, even a slight imbalance in mitochondrial iron homeostasis can be further amplified.

In addition to iron transport, NCOA4-mediated ferritinophagy is also a key mechanism regulating cellular iron availability (183). After ferritin is selectively degraded by autophagy, free iron is released into the reactive iron pool. If this process is unbalanced, it increases the likelihood of iron participating in Fenton reactions, promoting hydroxyl radical generation and accumulation of lipid peroxidation (184). In the context of COPD, smoke exposure, mitochondrial damage, and antioxidant system exhaustion collectively drive iron-dependent oxidative pressure to exceed the defense threshold. In other words, ferritinophagy and mitochondrial iron import together determine whether a cell enters a ferroptosis-susceptible state (7, 185).

The GSH/GPX4 system is a core defense against ferroptosis. GSH provides a reducing substrate for GPX4, enabling it to clear membrane lipid peroxides and prevent the propagation of lipid peroxidation chain reactions. Once GSH is depleted or GPX4 activity declines, membrane lipids rich in polyunsaturated fatty acids are more prone to irreversible peroxidation, thereby driving ferroptosis (186, 187). Furthermore, mitochondrial glutathione homeostasis and related transport mechanisms are also involved in this process, indicating that mitochondria not only affect ROS production but also participate in determining whether the antioxidant defense can be maintained (188). It is noteworthy that there may be bidirectional crosstalk between mitophagy and ferroptosis. Free iron released by ferritinophagy can enter mitochondria and expand the mitochondrial iron load, while damaged mitochondria themselves may also become an important source of the cellular reactive iron pool (189). If mitophagy is insufficient, damaged mitochondria persist, exacerbating mtROS generation and lipid peroxidation; if mitophagy is excessive or coexists with iron homeostasis imbalance, it may promote iron release and energy failure, further increasing susceptibility to ferroptosis (190).

Additionally, mitochondrial CoQ10 is also an important component of the anti-ferroptosis defense. CoQ10 can act as a lipid radical scavenger, participating in GPX4-independent ferroptosis resistance (191); the FSP1-related CoQ10 reduction system is mainly localized to the plasma membrane, but the mitochondrial CoQ10 pool is equally important for maintaining electron transport, membrane antioxidant capacity, and limiting lipid peroxidation (192). Therefore, the occurrence of ferroptosis in COPD may depend not only on the GSH/GPX4 defense mechanism but is also co-regulated by mitochondrial iron homeostasis, mitophagy status, and the CoQ10 antioxidant network.

**Figure 4:**
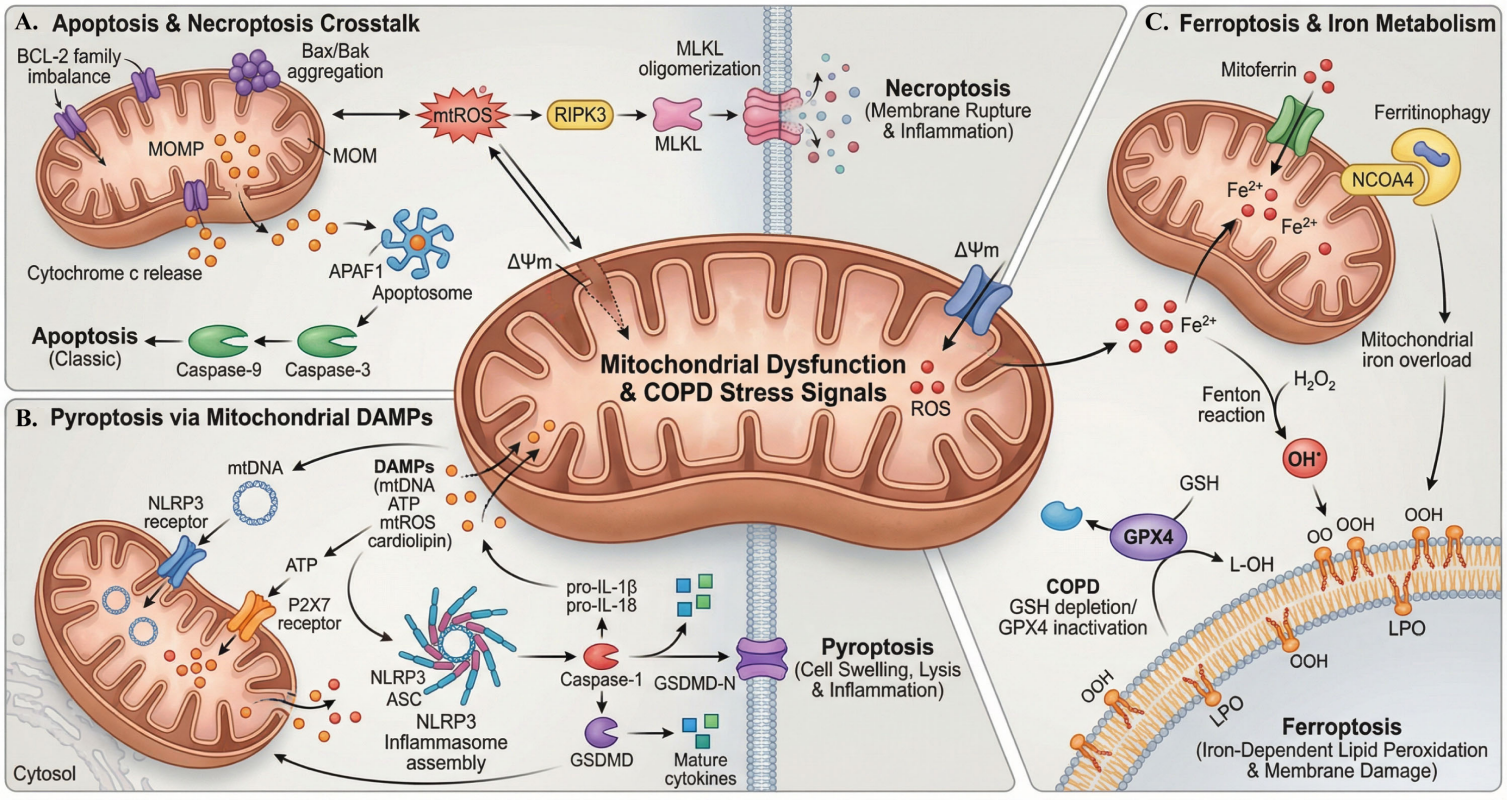
Cross-talk network between mitochondrial dysfunction and multiple cell death pathways in COPD. The central mitochondrial stress signal, characterized by decreased membrane potential and elevated ROS, can connect to three pathological processes: apoptosis/necroptosis, pyroptosis, and ferroptosis. **(A)** BCL-2 family imbalance, Bax/Bak oligomerization, and mitochondrial outer membrane permeabilization promote cytochrome c release and activate the classical apoptotic pathway, while mtROS can also amplify necroptosis via the RIPK3–MLKL axis. **(B)** Mitochondrial DAMPs such as mtDNA, ATP, mtROS, and cardiolipin can activate the NLRP3 inflammasome and the caspase-1/GSDMD axis, inducing pyroptosis and the mature release of inflammatory factors like IL-1β and IL-18. **(C)** Mitochondrial iron overload, GSH depletion, and GPX4 inactivation drive the accumulation of lipid peroxides and induce ferroptosis. Created in BioRender. Di, X. (2026) https://BioRender.com/ybdwf6j.

From a cellular and tissue localization perspective, ferroptosis may be more significant in cells such as airway epithelial cells, alveolar structural cells, and skeletal muscle that are under long-term oxidative load and metabolic stress. Airway epithelial cells are rich in membrane lipid components susceptible to oxidative damage and are directly exposed to smoke and particulate matter stimulation, making them more prone to lipid peroxidation accumulation (193); iron homeostasis imbalance and weakened GPX4 defense in the alveolar region may promote membrane structural damage and alveolar destruction; long-term energy metabolism disorders, elevated mtROS, and insufficient antioxidant defense in skeletal muscle may also enhance susceptibility to ferroptosis and contribute to extrapulmonary phenotypes (194). Ultimately, mitochondrial iron accumulation, elevated ROS, and heightened susceptibility to oxidation of membrane lipids can form a “high iron—high oxidation—high lipid peroxidation” microenvironment (195). Peroxidation of mitochondrial membranes and related lipids not only weakens mitochondrial metabolic capacity but also promotes the spread of lipid peroxidation on a larger scale, driving cells into the ferroptosis program (196). Therefore, ferroptosis in COPD arises from the convergence of mitochondrial iron homeostasis imbalance, impaired GSH/GPX4 defense, abnormal mitophagy, and a weakened CoQ10 antioxidant network. Compared with pyroptosis and necroptosis, ferroptosis more prominently features membrane damage caused by sustained lipid peroxidation and iron homeostasis imbalance, potentially playing a more persistent role in the progression of emphysema, chronic structural destruction, and extrapulmonary energy metabolism disorders. This also explains the potential value of targeting iron metabolism, lipid peroxidation, and the mitochondrial antioxidant system (**Figure 4**).

### 5.4 RCD network spatiotemporal integration: from parallel pathways to dynamic pathological patterns

Based on the above discussion, the RCD network in COPD is not a simple juxtaposition of multiple death pathways but a dynamic system that changes with cell type, injury intensity, and disease stage. Spatiotemporal specificity is not an additional concept independent of RCD mechanisms but a key principle for understanding when, where, and how different RCD modalities participate in COPD progression.

From the perspective of cell types, airway epithelial cells are the earliest and most continuously exposed to smoke and pollutants, making them more prone to decreased mitochondrial membrane potential, elevated mtROS, and barrier damage, thereby initiating RCD such as apoptosis, ferroptosis, and pyroptosis (14). Macrophages primarily mediate inflammatory amplification and immune regulation, and their mitochondrial damage is often associated with metabolic reprogramming, polarization imbalance, and inflammasome activation; thus, pyroptosis and necroptosis are more characteristic (197, 198). Evidence regarding airway smooth muscle cells is relatively limited, but their mitochondrial stress and oxidative damage may contribute to proliferation/apoptosis imbalance and airway remodeling (199). Appendicular skeletal muscle exhibits an extrapulmonary phenotype, where under conditions of chronic energy deficiency and oxidative stress, mitochondrial abnormalities are more likely to be associated with apoptosis, mitophagy imbalance, and increased susceptibility to ferroptosis.

From the perspective of disease stages, early COPD more commonly features a coexistence of stress adaptation and damage repair. At this stage, cells may ensure survival through adjustments in mitochondrial dynamics, moderate mitophagy, and metabolic reprogramming; if damage exceeds the compensatory threshold, increased apoptosis and cellular senescence accumulation gradually occur. As the disease progresses, persistent oxidative stress, DAMPs release, and chronic inflammation favor more inflammatory death modalities, such as increased pyroptosis and necroptosis; simultaneously, iron homeostasis imbalance and lipid peroxidation accumulation also predispose cells to ferroptosis (200, 201). That is, the early stage tends toward “stress-adaptation-partial apoptosis,” while the middle to late stages are more prone to “persistent damage-inflammatory amplification-structural destruction.” These transitions may not be solely determined by accumulated damage but may also involve active molecular switches. From the early to middle stages, decreased cellular ATP, limited caspase activity, or enhanced RIPK1/RIPK3 signaling may shift the death modality from caspase-dependent apoptosis to necroptosis (202). From the middle to late stages, when DAMPs release exceeds clearance capacity, the NLRP3 inflammasome and Caspase-1/GSDMD axis are more easily and persistently activated, driving pyroptosis and inflammatory amplification (203). In the late stage or systemic phenotype, depletion of the GSH/GPX4 defense system, iron homeostasis imbalance, and lipid peroxidation accumulation may establish ferroptosis as a predominant mode of sustained damage (204). The inflammatory microenvironment also influences death modality selection; for example, TNF-α, IL-1β, IL-18, and type I interferon-related signals can alter the relative activities of caspase, RIPK, and inflammasome pathways, thereby shaping different RCD patterns (202).

**Figure 5:**
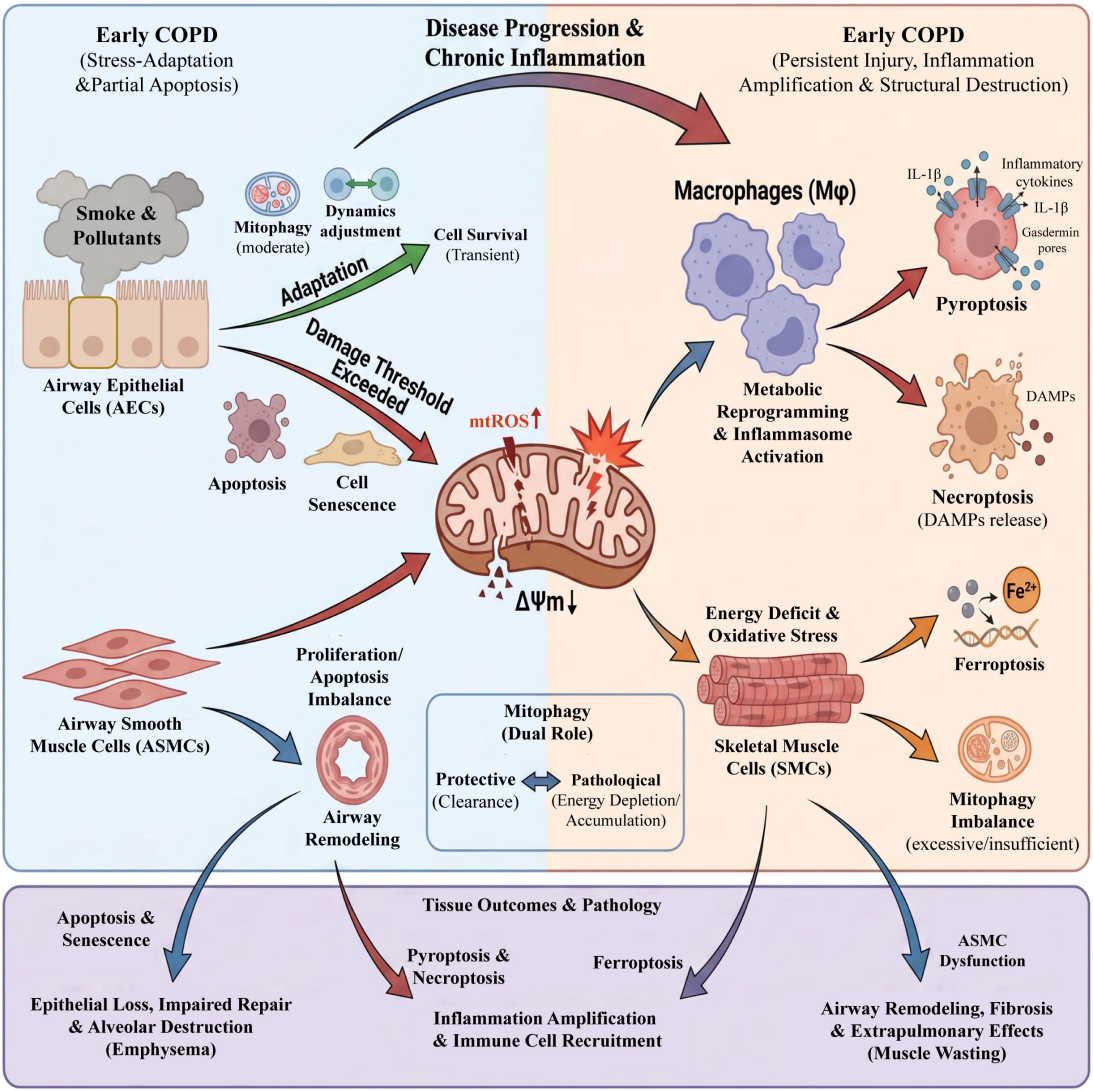
Spatiotemporal evolution characteristics of RCD in COPD, emphasizing mitochondrial dysfunction as a central hub. In early COPD, airway epithelial cells can achieve transient adaptation through moderate mitophagy and dynamic regulation under smoke and pollutant stimulation; when the damage threshold is exceeded, they shift toward apoptosis and cell aging, promoting impaired epithelial repair and alveolar destruction. As the disease progresses, elevated mtROS and decreased membrane potential drive macrophage inflammasome activation and energy deficits and oxidative stress in skeletal muscle cells, further inducing pyroptosis, necroptosis, and ferroptosis. Meanwhile, an imbalance between airway smooth muscle cell proliferation and apoptosis promotes airway remodeling. Overall, mitochondrial dysfunction drives COPD from early adaptation to late persistent inflammation, tissue destruction, and systemic effects by regulating RCD patterns across different cell types and stages. Created in BioRender. Di, X. (2026) https://BioRender.com/kjrgeso.

From the perspective of tissue outcomes, apoptosis and cell aging are more associated with epithelial cell loss, repair impairment, and alveolar structural degeneration (205); pyroptosis and necroptosis more readily trigger inflammatory factor release and local inflammatory amplification, driving immune cell recruitment and tissue destruction (206); ferroptosis prominently features lipid peroxidation, membrane damage, and oxidative stress amplification, and may participate in alveolar destruction and extrapulmonary energy metabolism disorders (207). Mitophagy has a biphasic effect: moderate mitophagy helps clear damaged mitochondria and limit inflammation, but insufficient or excessive mitophagy can lead to damage accumulation or energy depletion, respectively, thereby promoting cell death (208, 209). Additionally, the recently proposed concept of PANoptosis provides a supplementary perspective for understanding the RCD cross-network. PANoptosis is defined as the integrated activation of multiple RCD programs, such as apoptosis, necroptosis, and pyroptosis, through shared molecular platforms. Its core is not a single pathway but a complex regulatory network mediated by the interplay of molecules such as caspases, RIPK1/RIPK3, inflammasomes, and gasdermins (210). Although direct evidence for PANoptosis in COPD remains limited, in an environment with persistent DAMPs release, elevated mtROS, and inflammatory cytokine stimulation, multiple death programs may be activated synchronously or sequentially (211). Therefore, PANoptosis can serve as an important research direction for explaining RCD overlap and pathway switching in COPD in the future. Consequently, the RCD patterns in COPD exhibit distinct cell- and stage-specificity and collectively contribute to disease progression and pathological heterogeneity (**Figure 5**).

## 6 From mechanistic pathways to targeted interventions: mitochondria and RCD-directed therapy

Based on the aforementioned mechanisms, mitochondria and RCD-directed therapy can target different pathological nodes: mitochondria-targeted antioxidants primarily address mtROS accumulation, MQC regulation focuses on mitochondrial renewal and damage clearance, and RCD intervention targets downstream inflammatory or oxidative cell death (212). It is important to emphasize that the translational basis of each strategy varies significantly: pulmonary rehabilitation and exercise training have demonstrated clinical benefits; in contrast, MitoQ and other mitochondria-targeted antioxidants have yielded only limited human data in COPD, whereas strategies inhibiting ferroptosis, pyroptosis, and necroptosis remain primarily in the preclinical stage. Therefore, this section will further discuss the level of evidence, delivery barriers, and translational priorities in conjunction with mechanistic rationale.

### 6.1 Antioxidants and mitochondria-targeted antioxidants

Oxidative stress and mitochondrial dysfunction are critical pathological underpinnings of COPD, making interventions targeting ROS clearance and mitochondrial protection mechanistically rational. Compared with traditional non-targeted antioxidants, mitochondria-targeted antioxidants (MTAs) are designed to act specifically at the key sites of ROS generation, theoretically allowing for more direct attenuation of mtROS accumulation, stabilization of membrane potential, and limitation of inflammatory amplification (213, 214).

Currently, representative agents under extensive study include MitoQ and coenzyme Q10 (CoQ10). MitoQ can accumulate within mitochondria, thereby reducing oxidative damage and improving mitochondrial homeostasis; CoQ10 possesses dual attributes as an electron transport chain cofactor and an antioxidant molecule, contributing to the maintenance of energy metabolism and limitation of lipid peroxidation (215, 216). *In vitro* and animal experiments have shown that MTAs have the potential to reduce mtROS, improve membrane potential, and alleviate tissue damage (217, 218). However, direct clinical evidence in COPD patients remains limited, with existing studies focusing more on surrogate markers or extrapulmonary effects, and it has not yet been fully demonstrated that they can stably improve lung function, reduce exacerbations, or alter long-term outcomes. Therefore, the key question in this direction has shifted from “whether it is mechanistically rational” to “identifying suitable patient populations, determining optimal timing and delivery methods, and establishing long-term safety profiles.”

Delivery issues are a significant barrier to the translation of MTAs. Systemically administered MitoQ or CoQ10 must undergo multiple steps including absorption, plasma transport, tissue distribution, and mitochondrial uptake, and whether effective concentrations can be achieved in airway epithelial cells, alveolar cells, or alveolar macrophages remains unclear. CoQ10 is highly lipophilic, with individual variations in oral absorption and tissue distribution; although MitoQ has a mitochondrial targeting design, pulmonary tissue exposure, long-term accumulation, and cell-type selectivity still require further evaluation (219). Inhaled delivery may increase local pulmonary concentrations and reduce systemic exposure, but it still faces pharmacokinetic/pharmacodynamic barriers such as formulation stability, particle deposition site, macrophage uptake, airway irritation, and long-term safety (220). Therefore, future MTA studies should simultaneously assess pulmonary tissue drug exposure, mitochondrial functional endpoints, and clinical outcomes, rather than relying solely on peripheral oxidative stress markers.

### 6.2 Regulating mitochondrial quality control

MQC includes mitochondrial biogenesis, dynamics regulation, and mitophagy, and is a key mechanism for maintaining mitochondrial quantity, structure, and functional stability (221). Given the presence of insufficient mitochondrial renewal, dynamic imbalance, and abnormal clearance of damaged mitochondria in COPD, MQC represents an attractive direction for intervention.

From a translational perspective, the PGC-1α/SIRT1/AMPK axis and the PINK1/Parkin-related mitophagy pathway are two relatively practical entry points. Theoretically, activating the PGC-1α/SIRT1/AMPK axis can enhance mitochondrial biogenesis and metabolic adaptive capacity, particularly suitable for COPD subgroups with insufficient mitochondrial renewal, impaired energy metabolism, or significant skeletal muscle function decline (222); the PINK1/Parkin pathway focuses on the recognition and clearance of damaged mitochondria and may be more suitable for pathological states with high mtROS burden and significant accumulation of damaged mitochondria (223). In contrast, while directly targeting fusion/fission proteins is mechanistically clear, these processes are integral to normal cellular homeostasis; thus, systemic intervention may lead to off-target effects and safety concerns.

It is important to note that enhancing MQC is not invariably beneficial. In the early stages of mitochondrial damage, promoting moderate mitophagy and supporting biogenesis may help limit the spread of damage (224); whereas in chronic late stages or under sustained high-intensity stress, excessive activation of mitophagy may exacerbate ATP depletion and functional failure (225). Therefore, the therapeutic strategy may differ at different stages of COPD: early stages should prioritize maintaining adaptation and repair, while late stages require avoiding uncontrolled clearance and irreversible energy failure. Current MQC regulation research is still mainly derived from cellular and animal models, and future studies should further clarify which patients have reversible MQC abnormalities, which indicators can be used for stratification, and whether different regulatory strategies are needed at different disease stages (226).

### 6.3 Targeting necroptosis and pyroptosis

Both necroptosis and pyroptosis have significant pro-inflammatory characteristics, so inhibiting these two types of cell death programs is expected to reduce DAMP release, attenuate inflammatory amplification, and alleviate airway and alveolar damage (227, 228). In terms of necroptosis, the RIPK1/RIPK3/MLKL axis is the main target. Experimental studies have shown that smoke exposure can promote the activation of RIPK3 and MLKL, while genetic knockout or pharmacological intervention in related pathways can reduce airway inflammation, alveolar destruction, and emphysema-like changes (229). For pyroptosis, the NLRP3 inflammasome, caspase-1, and GSDMD form a direct therapeutic cascade; inhibiting NLRP3 or downstream pyroptosis pathways can reduce the release of IL-1β and IL-18, and alleviate epithelial damage and inflammatory infiltration (230–232). Since mtROS and mtDNA release can simultaneously participate in the amplification of necroptosis and pyroptosis, combined inhibition of upstream mitochondrial damage and downstream inflammatory cell death signals is theoretically synergistic (233). However, this direction is still primarily a preclinical pathway, with very limited drug validation at the patient level. At the same time, necroptosis and pyroptosis pathways are also involved in host defense, infection response, and inflammatory clearance, and excessive inhibition may weaken anti-infection capabilities (234). Therefore, while this strategy is mechanistically attractive, it still requires more safety and population stratification evidence before forming a clear clinical pathway.

### 6.4 Targeting ferroptosis

Ferroptosis is characterized by the interplay of iron homeostasis imbalance, lipid peroxidation, and mitochondrial damage, and represents one of the potential therapeutic avenues for COPD. Smoking, chronic oxidative stress, GSH depletion, and weakened GPX4 defenses can collectively increase the susceptibility of COPD tissues to ferroptosis and contribute to alveolar damage, exacerbation of inflammation, and the formation of some extrapulmonary phenotypes (235). Interventions targeting ferroptosis mainly include three types of strategies: (1) reducing reactive iron load, exemplified by the use of iron chelators to attenuate Fenton reaction activity (236); (2) inhibiting the lipid peroxidation chain reaction via agents such as Ferrostatin-1 (237); and (3) enhancing endogenous antioxidant defenses, particularly by restoring the GSH/GPX4 and Nrf2-mediated protective axes (238). These strategies have shown protective potential in experimental models, but therapeutic approaches targeting ferroptosis in COPD are still primarily grounded in mechanistic research and preclinical evidence. Therefore, ferroptosis is currently more suitable to be positioned as a “potential therapeutic target” rather than a mature strategy with a clear clinical pathway. Future efforts need to clarify the true contribution of ferroptosis in different COPD phenotypes and establish stratification indicators that reflect iron homeostasis, lipid peroxidation, and GPX4 functional status.

### 6.5 Exercise rehabilitation and mitochondrial function remodeling

In addition to pharmacological interventions, exercise training and pulmonary rehabilitation are among the few strategies in COPD with clear clinical benefits that can also improve mitochondrial function. COPD patients often have decreased skeletal muscle mitochondrial density, reduced oxidative phosphorylation efficiency, and impaired exercise tolerance, while regular exercise can promote mitochondrial biogenesis through the AMPK/SIRT1–PGC-1α axis, improve oxidative metabolic capacity, and enhance antioxidant adaptation (40). From a translational perspective, pulmonary rehabilitation not only improves exercise tolerance and quality of life but may also partially reverse the extrapulmonary mitochondrial phenotype in COPD by promoting skeletal muscle mitochondrial renewal, improving metabolic flexibility, and reducing systemic inflammatory burden (239). In contrast to drug targets that remain in the early translational stage, exercise rehabilitation offers distinct advantages, including high clinical accessibility, established efficacy, and a favorable safety profile. Future research could further incorporate PGC-1α, GDF15, FGF21, peripheral blood oxidative stress markers, and muscle function parameters into pulmonary rehabilitation studies to determine which patients are most likely to benefit at the mitochondrial functional level.

### 6.6 Combination and personalized strategies

If the preceding sections answer “which pathological nodes can be intervened upon,” then combination and personalized strategies must answer “who should receive treatment, how to combine, and what indicators to use to judge efficacy.” The premise of combination therapy is mechanistic phenotyping. Not all COPD patients are dominated by the same pathological axis: some patients have more prominent mitochondrial oxidative stress and epithelial damage, others are characterized by inflammasome activation and enhanced pyroptosis, and still others are closer to phenotypes dominated by iron homeostasis imbalance, emphysema progression, or skeletal muscle mitochondrial function decline (240, 241). Therefore, molecular biomarkers such as cf-mtDNA, 8-OHdG, MDA, GDF15, FGF21, as well as NLRP3, GSDMD, and GPX4, can serve as criteria for patient stratification and efficacy evaluation in exploratory studies (242).

Combination regimens should not be a mere additive combination of drugs but should be tailored to the dominant pathological axis. Several combinations with more mechanistic logic can be considered: First, for patients with “high mitochondrial oxidative stress load,” MitoQ or CoQ10 combined with long-acting bronchodilators/inhaled corticosteroids could be explored to control airway symptoms and inflammation with standard inhaled therapy while reducing mtROS and mitochondrial damage through MTAs (243, 244); Second, for patients with “active inflammasome/pyroptosis,” NLRP3 pathway inhibitors combined with mitochondria-targeted antioxidants could be explored to simultaneously reduce DAMP-driven inflammasome activation and upstream mtROS sources (245); Third, for patients with “ferroptosis susceptibility” or significant emphysema progression, iron homeostasis regulation or lipid peroxidation inhibition strategies combined with Nrf2/GSH/GPX4 supportive therapy could be considered to simultaneously reduce reactive iron load and enhance anti-lipid peroxidation capacity (20). Fourth, for patients with “skeletal muscle dysfunction/extrapulmonary metabolic abnormalities,” standard intrapulmonary treatment should be combined with pulmonary rehabilitation, nutritional support, and PGC-1α-related mitochondrial biogenesis promotion strategies to improve exercise tolerance and systemic mitochondrial phenotype (246, 247). Future research also needs to clarify endpoint selection in advance. Simply observing a decrease in short-term inflammatory markers is insufficient to determine whether these strategies have real clinical value. A more comprehensive endpoint system should simultaneously cover mechanistic and clinical levels: the former includes changes in oxidative stress, biomarkers, mitochondrial function, and RCD pathway activity; the latter includes lung function, exacerbation frequency, exercise tolerance, radiographic progression of emphysema, skeletal muscle function, and quality of life. Only by connecting mechanistic signals with clinical benefits can combination and personalized strategies move from concept to verifiable pathways (**Table 3**).

**Table 3:** Potential therapeutic strategies and translational positioning of COPD targeting mitochondria and regulated cell death pathways.

Strategy category	Primary target/mechanism of action	Representative pathway or candidate intervention	Current level of evidence	Potential applicable phenotype	Key translational issues	Reference
Antioxidants and Mitochondria-Targeted Antioxidants	Scavenge mtROS, stabilize mitochondrial membrane potential, limit oxidative stress and inflammatory amplification.	MitoQ, CoQ10, and other mitochondria-targeted antioxidants	Extensive *in vitro* and animal studies; limited human COPD data	High mitochondrial oxidative stress load phenotype; smoking exposure-related epithelial injury phenotype.	Need to clarify lung tissue exposure, optimal dosing timing, long-term safety, and its efficacy in improving lung function, acute exacerbations, and long-term outcomes.	(215–218)
Regulation of Mitochondrial Quality Control (MQC)	Improve mitochondrial biogenesis, mitochondrial dynamics, and clearance of damaged mitochondria	PGC-1α/SIRT1/AMPK axis; PINK1/Parkin pathway; mitophagy modulators	Primarily cellular and animal experiments	Insufficient mitochondrial turnover phenotype; skeletal muscle dysfunction phenotype; high mtROS load phenotype.	Need to distinguish disease stages of “promoting repair” vs. “avoiding excessive clearance,” establish stratified indicators such as PGC-1α, PINK1/Parkin, LC3-II/I, p62.	(223–226)
Targeting Necroptosis and Pyroptosis	Inhibit pro-inflammatory cell death, reduce DAMP release, inhibit inflammasome activation, and prevent alveolar damage	RIPK1/RIPK3/MLKL axis inhibition; NLRP3, caspase-1, GSDMD pathway inhibition	Primarily preclinical evidence; insufficient validation at the patient level	Active inflammasome/pyroptosis phenotype; DAMPs-driven inflammatory amplification phenotype.	Need to be cautious about inhibiting host defense, infection clearance, and inflammatory repair; should prioritize validation in populations enriched with inflammatory markers.	(229–233)
Targeting Ferroptosis	Reduce reactive iron load, inhibit lipid peroxidation, restore GSH/GPX4/Nrf2 defense axis	Iron chelating agents; Ferrostatin-1; Nrf2/GPX4-related drugs or natural products	Primarily mechanistic studies and preclinical evidence.	Ferroptosis-susceptible phenotype; significant emphysema progression phenotype; high lipid peroxidation load phenotype.	Need to establish stratified indicators for iron homeostasis, lipid peroxidation, and GPX4 functional status, and balance the risks of systemic iron metabolism intervention.	(235–238)
Exercise Rehabilitation and Mitochondrial Function Remodeling	Promote skeletal muscle mitochondrial biogenesis, improve oxidative metabolic capacity and exercise tolerance.	Pulmonary rehabilitation, exercise training, nutritional support; AMPK/SIRT1–PGC-1α axis activation.	Supported by existing evidence of clinical benefit; represents a relatively mature non-pharmacological intervention.	Skeletal muscle dysfunction phenotype; extrapulmonary metabolic abnormality phenotype; decreased exercise tolerance phenotype.	Can further evaluate mitochondrial benefits by combining PGC-1α, GDF15, FGF21, oxidative stress indicators, and muscle function parameters.	(239)
Combination and Personalized Strategies Combination therapy based on the dominant pathological axis, rather than empirical drug stacking	Combination therapy based on the dominant pathological axis, rather than empirical drug stacking.	MTAs + standard inhalation therapy; NLRP3 inhibition + mitochondrial antioxidants; Ferroptosis inhibition + Nrf2/GSH/GPX4 support; Intrapulmonary treatment + pulmonary rehabilitation.	Concept integration and exploratory stage	COPD subgroups with clear mechanistic classification	Need to establish a candidate biomarker system including cf-mtDNA, 8-OHdG, MDA, GDF15, FGF21, NLRP3, GSDMD, GPX4, and connect mechanistic endpoints with clinical endpoints.	(242–245)

## 7 Translational challenges and future perspectives

Although the “mitochondrial dysfunction-oxidative stress-regulated cell death” framework provides an integrated explanation for the pathogenesis of COPD, its clinical translation still faces multiple challenges. Existing evidence mainly comes from animal models, cell experiments, and cross-sectional clinical observations, which are insufficient to determine the causal weight of each mechanistic node in different COPD subtypes and disease stages. Furthermore, a paucity of robust biomarker panels persists that can simultaneously reflect mitochondrial damage, oxidative stress burden, and RCD activity, and the intervention window remains unclear. Therefore, future research should gradually shift from single-mechanism validation to a continuous translational pathway of “mechanism-based classification—biomarker validation—proof-of-concept trials—clinical endpoint assessment.”

### 7.1 Limitations of current evidence

Although studies on mitochondrial dysfunction and its relationship with RCD in COPD have increased significantly, the overall evidence base still has limitations. First, most existing studies are cross-sectional observations, *in vitro* experiments, and animal models, which can yield mechanistic insights but are insufficient to adequately recapitulate the long-term progression, recurrent exacerbations, and multisystem involvement of human COPD (248, 249). Particularly in cigarette smoke exposure models, many results are more suitable for explaining specific stages or local processes rather than being equivalent to the complex and persistent disease state of clinical patients (14). Second, COPD exhibits significant heterogeneity, with differences among patients in exposure background, inflammatory phenotype, emphysema severity, exacerbation tendency, and extrapulmonary complications. Therefore, “mitochondrial dysfunction” should not be regarded as a uniform mechanistic label for all patients (250). Some patients may primarily present with epithelial damage and alveolar destruction, others may show more prominent inflammatory maintenance and immune imbalance, and still others may manifest skeletal muscle dysfunction and systemic metabolic abnormalities (251). If this heterogeneity is ignored, mechanistic research will struggle to translate into actionable treatment strategies. Third, many key mechanisms related to mtROS, NLRP3, pyroptosis, ferroptosis, UPRmt, or mitochondrial membrane permeability were initially elucidated in the context of cardiovascular, renal, neurological, or oncology research (252, 253). While these findings are heuristic, they cannot automatically equate to pathological reality in COPD. Currently, many mechanistic pathways are biologically plausible but lack direct validation in COPD-specific models and patient samples (254). Finally, clinical translational evidence remains weak. Although antioxidants, mitochondrial protectants, metabolic regulators, and experimental interventions have been proposed to potentially improve COPD-related mitochondrial damage, most remain in preclinical or early exploratory stages, with limited high-quality trial data supporting real clinical benefits (114).

### 7.2 Biomarkers and disease classification

If research on mitochondrial dysfunction and the RCD network is to truly move toward clinical application, the key is not to discover more single molecules but to establish a mechanism-based biomarker system that can **facilitate** patient stratification (255). COPD has long been assessed primarily based on symptoms, lung function, and exacerbation risk, but these indicators are insufficient to reflect the dominant pathological processes underlying the disease in individual patients (256). Therefore, a more meaningful question is: Can we identify patients with “high mitochondrial damage burden,” “active inflammatory RCD,” “ferroptosis susceptibility,” or “the extrapulmonary mitochondrial failure/sarcopenia-related phenotype” (257).

Based on current evidence, four candidate mechanistic subtypes can be preliminarily proposed. The first is the oxidative stress-mitochondrial damage type, which may be characterized by elevated cf-mtDNA, 8-OHdG, and MDA, along with abnormal mitokines such as GDF15 and FGF21, indicating high mitochondrial stress and systemic metabolic burden (258). The second is the inflammasome-pyroptosis active type, likely characterized by enhanced NLRP3-related signaling, elevated IL-1β/IL-18, and prominent GSDMD cleavage features (259). The third is the iron homeostasis imbalance-ferroptosis susceptible type, possibly accompanied by abnormalities in GPX4, SLC7A11, ACSL4, and lipid peroxidation indicators (260, 261). The fourth is the extrapulmonary mitochondrial failure/sarcopenia-related type, often presenting with decreased exercise tolerance, skeletal muscle dysfunction, and systemic metabolic changes, requiring simultaneous assessment of intrapulmonary and extrapulmonary mechanisms (262, 263).

It should be emphasized that the above four subtypes should currently be regarded as hypothesis-generating research frameworks rather than mature clinical classification systems. At this stage, it is not possible to accurately estimate the prevalence of each subtype in the COPD population, nor is it clear whether these subtypes are stable in individual patients or may shift with exacerbations, disease progression, smoking cessation, treatment, or rehabilitation interventions. From a clinical perspective, emphysema-dominant patients may be more likely to exhibit mitochondrial damage and ferroptosis susceptibility features, chronic bronchitis or frequent exacerbation patients may lean toward an inflammasome/pyroptosis active state, and those with significant exercise intolerance, sarcopenia, or metabolic abnormalities may be closer to the extrapulmonary mitochondrial failure phenotype (136). However, these inferences still require validation through prospective cohorts and mechanistic biomarkers and cannot be directly used as clinical diagnostic criteria.

Future validation of this classification framework can follow this pathway: First, integrate clinical phenotypes, CT imaging, lung function, exacerbation history, and multi-omics data in a discovery cohort to identify candidate mechanistic subgroups; second, validate biomarker combinations using peripheral blood, sputum, bronchial brushings, or tissue samples in independent cohorts; third, assess the relationship between each subtype and lung function decline, exacerbations, emphysema progression, skeletal muscle function, and mortality risk; finally, test whether different subtypes exhibit differential treatment responses in mechanism-enriched clinical trials. Only after completing this validation chain can biomarkers transition from “mechanism-proving tools” to a basis for disease classification and treatment selection (264–266).

### 7.3 Multi-omics and systems biology integration

The value of multi-omics and systems biology lies not only in increasing data dimensions but also in helping address three key issues in COPD mitochondrial and RCD research: identifying subtypes, finding bridging molecules, and predicting treatment responses (267, 268). Single biomarkers are insufficient to capture the complex molecular heterogeneity of COPD patients, whereas combined analyses of transcriptomics, proteomics, metabolomics, lipidomics, and mitochondrial-specific omics are more likely to identify patient subgroups characterized by different pathological axes (269). For example, some patients may exhibit more prominent mitochondrial respiratory chain abnormalities and metabolic reprogramming; others may show inflammasome activation and immune cell remodeling; and still others may be characterized by iron metabolism disorders and lipid peroxidation (270).

Future multi-omics integration should further incorporate information at the cell type and regulatory level. Single-cell RNA sequencing and single-cell assay for transposase-accessible chromatin sequencing can identify specific mitochondrial stress and RCD signals in airway epithelial cells, alveolar epithelial cells, macrophages, endothelial cells, smooth muscle cells, and skeletal muscle cells. Epigenomics, including DNA methylation, histone modification, and chromatin accessibility, may help explain why some patients exhibit persistent mitochondrial dysfunction and inflammatory memory even after smoking cessation. Lipidomics is particularly important for ferroptosis research, as it can capture changes in membrane lipid peroxidation, polyunsaturated fatty acid remodeling, and CoQ10-related antioxidant defenses. Mitochondrial-specific omics, such as mtDNA sequencing, mtDNA copy number/heteroplasmy analysis, and mitochondrial proteomics, can directly assess mitochondrial genome damage, respiratory chain complex abnormalities, and alterations in mitochondrial protein homeostasis. Large COPD cohorts and consortia, such as SPIROMICS, COPDGene, and related multi-omics projects, have already integrated clinical phenotypes, imaging, transcriptomics, proteomics, metabolomics, or genetic information to identify molecular features associated with emphysema, airway disease, exacerbations, inflammatory burden, and systemic phenotypes (271). Although not all of these studies are centered on mitochondria or RCD, their data frameworks suggest that molecular subtypes in COPD may be closely related to energy metabolism, immune inflammation, oxidative stress, and tissue remodeling pathways, providing important resources for subsequent validation of mitochondrial-RCD classification. Future studies can further overlay mtDNA, lipidomics, mitochondrial proteomics, and single-cell data on these cohorts to clarify whether mitochondrial abnormalities are drivers, accompanying markers, or predictors of treatment response for certain clinical phenotypes.

Additionally, multi-omics can help identify “bridging molecules” connecting different pathological layers. Many studies have separately observed mitochondrial damage, inflammatory responses, cell death, and extrapulmonary phenotypes, but the key nodes truly linking these processes remain unclear (272, 273). Systems biology analysis can screen for regulatory nodes from complex networks that simultaneously associate with mitochondrial homeostasis, RCD signaling, and clinical phenotypes, such as pathways involving SIRT1, Nrf2, PINK1/Parkin, NLRP3, and GPX4 (274). These bridging molecules are more likely than simple differentially expressed genes to become targets or classification bases. Future multi-omics integration should also serve treatment response prediction, such as identifying who is more likely to benefit from antioxidant therapy, inflammatory RCD inhibition, or pulmonary rehabilitation/mitochondrial biogenesis promotion strategies (275, 276). Therefore, valuable multi-omics research should align molecular networks with clinical problems to form an interpretable, verifiable, and classifiable mechanistic framework.

### 7.4 Clinical trials and guideline transition

The greatest challenge in moving from mechanistic research to clinical application is not to prove again that “mitochondria are important,” but to translate mechanistic information into implementable trial designs and evaluable clinical pathways (277). At present, mitochondrial and RCD-targeted therapies in COPD are still in the developmental stage, have not entered routine guideline frameworks, and have not yet formed stable, reproducible clinical strategies.

First, future clinical trials need to address patient selection. If trial subjects lack mechanistic stratification and are enrolled solely based on a broad COPD diagnosis, genuine therapeutic signals may be diluted (278). A more feasible design is enrichment based on candidate biomarkers and mechanistic phenotypes, such as prioritizing patients with high oxidative stress burden, elevated cf-mtDNA, rapid emphysema progression, or significant skeletal muscle dysfunction. Second, endpoint design needs to be more layered. For mechanism-guided interventions, relying solely on short-term lung function changes as a measure of efficacy is often insufficiently sensitive (279). A more reasonable strategy is to set parallel mechanistic and clinical endpoints: the former includes changes in cf-mtDNA, 8-OHdG, MDA, GDF15, inflammasome-related indicators, or ferroptosis-related molecules; the latter includes exacerbations, exercise tolerance, imaging-based emphysema progression, skeletal muscle function, and quality of life improvement (280, 281). Only when mechanistic improvements correspond to clinical outcome improvements can relevant therapies move toward guideline inclusion (282). Third, combination therapy models may be more suitable for the clinical reality of COPD than monotherapy approaches. The current COPD management system is still based on standard pathways including bronchodilators, inhaled corticosteroids, smoking cessation, pulmonary rehabilitation, and exacerbation management (283). Future mitochondrial or RCD-targeted interventions are more likely to serve as supplements to standard therapy rather than replacements. That is, the more realistic question is not “can it cure COPD alone,” but “can it provide additional benefits to patients with specific mechanistic subtypes on top of standard therapy.” Therefore, in the foreseeable future, mitochondrial and RCD-related interventions are better positioned as mechanism-guided exploratory treatment pathways; their entry into guidelines requires high-quality stratified clinical trials, reproducible biomarker validation, and accumulation of evidence demonstrating tangible clinical benefits.

### 7.5 Future research directions

Based on current evidence foundations and translational bottlenecks, research on mitochondrial dysfunction and the RCD network in COPD urgently needs to shift from “mechanism expansion” to a continuous pathway of “mechanism validation—classification establishment—clinical translation.” First, future research should place greater emphasis on cell-type specificity. COPD pathogenesis is not driven by a single cell population; rather, distinct cell types exhibit heterogeneous responses to mitochondrial damage and RCD activation. Airway epithelial cells and alveolar epithelial cells are more directly exposed to smoke and environmental stimuli, macrophages are more involved in inflammatory amplification and immune regulation, airway smooth muscle cells are related to airway remodeling, and skeletal muscle cells reflect extrapulmonary energy metabolism and systemic damage (284). Key questions include: which type of mitochondrial abnormality dominates in different cell types, which RCD is more decisive, and how these mechanisms transition from early stress adaptation to late persistent damage, senescence accumulation, and structural remodeling with disease progression (285). Second, mechanism-based biomarkers and disease classification systems will be key to precision therapy. Future research should not merely focus on screening differentially expressed genes but should develop biomarker panels that can be derived from clinical samples, validated in independent cohorts, and linked to outcomes (286). Establishing candidate biomarker panels centered on core pathological axes such as oxidative stress, mitochondrial damage, inflammatory RCD, and iron homeostasis imbalance will help integrate disparate mechanistic studies into a more clinically meaningful classification framework (287). In other words, the most critical step from mechanism discovery to precision intervention is establishing a classification system that supports patient stratification and efficacy prediction.

**Figure 6:**
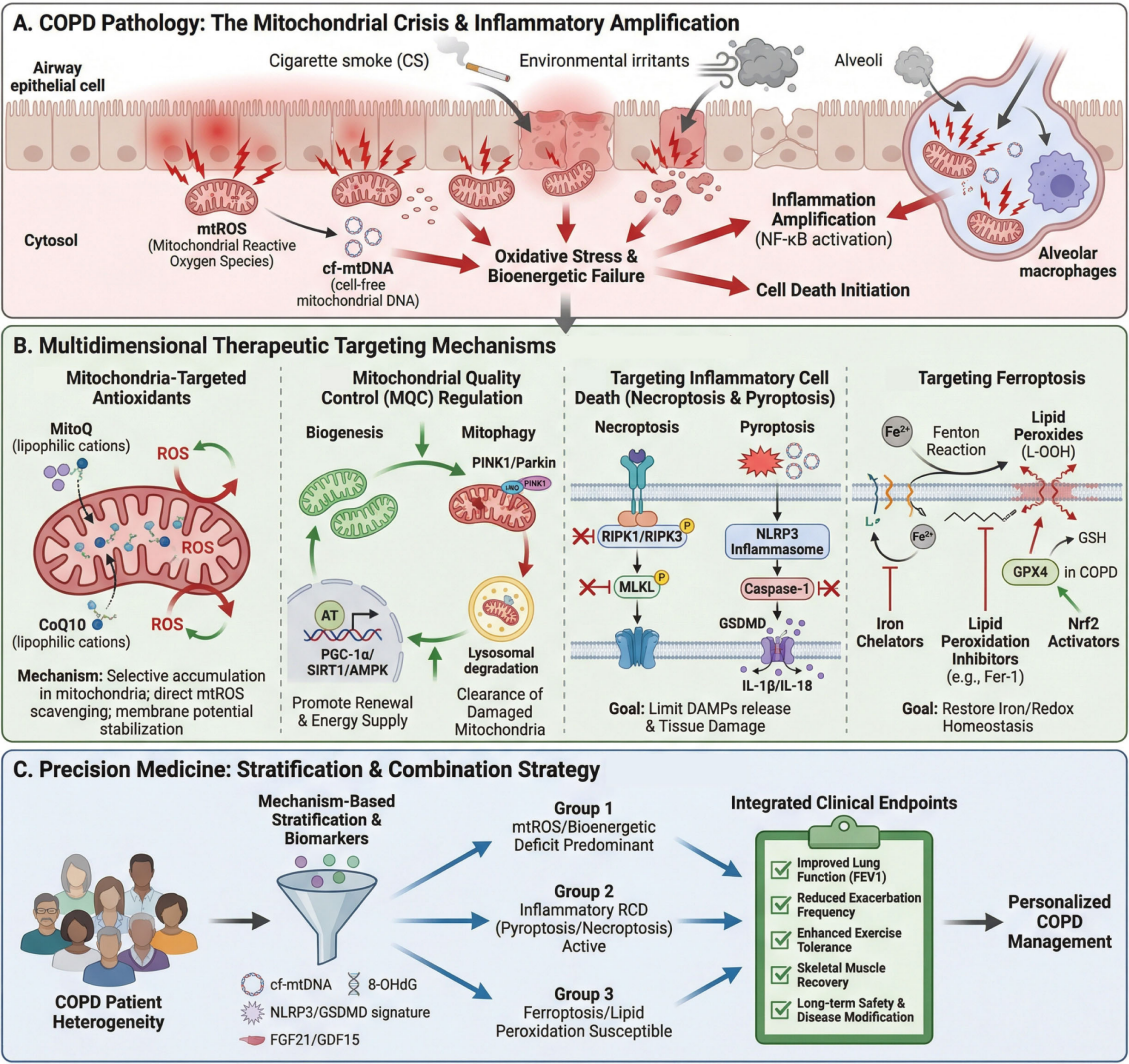
This figure outlines a potential precision intervention framework for pathways related to mitochondrial dysfunction and regulated cell death in COPD. **(A)** Cigarette smoke and environmental stimuli can induce mitochondrial damage in airway epithelial and alveolar macrophages, leading to the release of mtROS and cf-mtDNA, thereby triggering oxidative stress, bioenergetic failure, inflammatory amplification, and cell death initiation. **(B)** Multidimensional therapeutic targets, including mitochondrial-targeted antioxidants, mitochondrial quality control regulation, inhibition of inflammatory cell death pathways, and ferroptosis intervention, aim to restore mitochondrial homeostasis, limit DAMPs release, and reduce tissue damage. **(C)** Mechanism-based precision medicine strategy, which involves classifying patients using biomarkers such as cf-mtDNA, 8-OHdG, NLRP3/GSDMD features, and FGF21/GDF15, and accordingly optimizing combination therapy to improve lung function, reduce exacerbations, enhance exercise tolerance, and support individualized COPD management. Created in BioRender. Di, X. (2026) https://BioRender.com/12340sp.

On this basis, mechanism-guided small-scale proof-of-concept trials should become an important focus for the next stage. Rather than directly conducting large-scale trials without stratification, it is better to first carry out enriched exploratory studies on patient subgroups with relatively clear mechanistic signals. Such trials can prioritize patients with high mitochondrial damage burden or active specific RCD pathways and simultaneously set molecular and clinical endpoints to assess whether the intervention truly provides continuous evidence from mechanistic improvement to clinical benefit. Only by systematically validating the linkage between “mechanism-biomarker-efficacy” can relevant treatment strategies move from theoretical potential to verifiable clinical pathways. Therefore, future research should focus on mechanistic classification, biomarker validation, and stratified clinical trials to drive mitochondrial and RCD-related mechanisms from theoretical frameworks to verifiable translational pathways (**Figure 6**).

## 8 Conclusion

Mitochondrial dysfunction is a key link in the pathological progression of COPD, with its significance not only lying in impaired energy metabolism but also in its position as a convergence point among oxidative stress, chronic inflammation, cell aging, and regulated cell death. Persistent mitochondrial damage can drive airway epithelial injury, the perpetuation of inflammation, and tissue remodeling through mtROS accumulation, abnormal mtDNA release, quality control imbalance, and metabolic reprogramming. Therefore, integrating COPD-related pathological processes from the common hub of mitochondria helps to more systematically understand the molecular basis of disease progression and extrapulmonary phenotypes. Current evidence indicates that various forms of regulated cell death, including apoptosis, necroptosis, pyroptosis, and ferroptosis, are coupled with mitochondrial damage and together constitute a pro-inflammatory and pro-injury interconnected network in COPD. Although different cell death programs have their own characteristics, they often share upstream basic events such as altered mitochondrial membrane permeability, enhanced oxidative stress, DAMPs release, and iron homeostasis imbalance. However, most current evidence primarily comes from cell experiments and animal models, with relatively limited direct evidence from COPD patient samples and clinical studies. Therefore, the disease specificity and clinical applicability of the related mechanistic pathways still require further validation. In the future, the focus of this field should shift from merely accumulating mechanistic clues to establishing a more mechanistically robust and translatable research framework. Specifically, it is necessary to further advance cell type- and disease stage-specific mechanistic validation, establish a mechanistic biomarker and disease classification system centered on the mitochondrial damage and RCD network, and conduct stratified, proof-of-concept clinical studies on this basis. Only by truly linking mechanistic research, biomarker screening, and intervention pathway evaluation can research on mitochondria and RCD move from pathological explanation to verifiable precision intervention strategies.

## References

[1] Koo HK Bhatt SP . Imaging in chronic obstructive pulmonary disease: Ready for prime time? Tuberc Respir Dis (Seoul). (2026) 89:143–53. doi: 10.4046/trd.2025.0202 41736372 PMC13065400

[2] Zhang Y Ren T Xue J Yu Y Zhou X Hu X . Application of extracellular vesicles in the diagnosis and treatment of chronic obstructive pulmonary disease (review). Mol Med Rep. (2026) 33:88. doi: 10.3892/mmr.2026.13798 41543163 PMC12848478

[3] Ranatunga S Pascoe CD . Maternal diabetes and lung health: An unexplored risk factor for COPD? Am J Physiol Lung Cell Mol Physiol. (2025) 329:L126–l133. doi: 10.1152/ajplung.00046.2025 40464374

[4] Polverino F Sin DD . The developmental origins of asthma and COPD. Annu Rev Physiol. (2026) 88:513–35. doi: 10.1146/annurev-physiol-042924-084007 40967241 PMC13285957

[5] Duan P Wang Y Lin R Zeng Y Chen C Yang L . Impact of early life exposures on COPD in adulthood: A systematic review and meta-analysis. Respirology. (2021) 26:1131–51. doi: 10.1111/resp.14144 34541740

[6] Xie M Chang Q Li C Liu Q Weng J Feng Y . TRPA1 mediates ozone-induced murine model of COPD through the Wnt5a/GSK-3β/β-catenin pathway. Environ pollut. (2026) 393:127717. doi: 10.1016/j.envpol.2026.127717 41587734

[7] Xie J Li P Du J Li S Li Z Zhang J . Dynamic changes of immune cells and therapeutic responses in experimental models of COPD. Front Immunol. (2026) 17:1698508. doi: 10.3389/fimmu.2026.1698508 41822510 PMC12975610

[8] Yang IA Jenkins CR Salvi SS . Chronic obstructive pulmonary disease in never-smokers: Risk factors, pathogenesis, and implications for prevention and treatment. Lancet Respir Med. (2022) 10:497–511. doi: 10.1016/s2213-2600(21)00506-3 35427530

[9] Soleimanifar N Assadiasl S Kalateh E Hassanvand MS Sadr M Mojtahedi H . Circulating exosomes and ambient air pollution exposure in COPD. Chronic Obstr Pulm Dis. (2023) 10:412–21. doi: 10.15326/jcopdf.2023.0400 37676651 PMC10699492

[10] Deolmi M Decarolis NM Motta M Makrinioti H Fainardi V Pisi G . Early origins of chronic obstructive pulmonary disease: Prenatal and early life risk factors. Int J Environ Res Public Health. (2023) 20:2294. doi: 10.3390/ijerph20032294 36767660 PMC9915555

[11] Wang Y Bao Y Deng X Duan H Wang Y Li H . Lipophagy in chronic obstructive pulmonary disease: Mechanistic insights and emerging therapeutic targets. BioMed Pharmacother. (2026) 196:119153. doi: 10.1016/j.biopha.2026.119153 41734567

[12] Di Paola M Reitz CJ Kuzmanov U Jia K Gramolini AO . Subcellular proteomic analyses reveal REEP5 knockdown in the mouse heart disrupts mitochondrial networks. Mol Cell Proteomics. (2026) 25:101527. doi: 10.1016/j.mcpro.2026.101527 41672147 PMC13014923

[13] Li CL Liu SF . Cellular and molecular biology of mitochondria in chronic obstructive pulmonary disease. Int J Mol Sci. (2024) 25:7780. doi: 10.3390/ijms25147780 39063022 PMC11276859

[14] He Q Li P Han L Yang C Jiang M Wang Y . Revisiting airway epithelial dysfunction and mechanisms in chronic obstructive pulmonary disease: The role of mitochondrial damage. Am J Physiol Lung Cell Mol Physiol. (2024) 326:L754–l769. doi: 10.1152/ajplung.00362.2023 38625125

[15] Sharma A Raut SS Shukla A Singh A Mishra A . Therapeutic targeting of the mitochondrial dysfunction-PANoptosis axis: Mechanistic insights and emerging strategies. Transl Res. (2026) 291:1–39. doi: 10.1016/j.trsl.2026.03.004 41839231

[16] Yang X Huang Y Zheng A Mu H Cui J . TNFRSF17 knockdown alleviates mitochondrial dysfunction and inflammation in COPD through suppression of the JAK2/STAT3 pathway. Drug Dev Res. (2026) 87:e70234. doi: 10.1002/ddr.70234 41586567

[17] Wenzhe L Boyang X Yuchao G Bimcle R Yue Y . Mitochondrial and ER stress crosstalk in TBI: Mechanistic insights and therapeutic opportunities. Front Cell Neurosci. (2025) 19:1697060. doi: 10.3389/fncel.2025.1697060 41480492 PMC12753455

[18] Bellomo F De Rasmo D . Cystinosis and cellular energy failure: Mitochondria at the crossroads. Int J Mol Sci. (2026) 27:630. doi: 10.3390/ijms27020630 41596280 PMC12840795

[19] Liu YB Hong JR Jiang N Jin L Zhong WJ Zhang CY . The role of mitochondrial quality control in chronic obstructive pulmonary disease. Lab Invest. (2024) 104:100307. doi: 10.1016/j.labinv.2023.100307 38104865

[20] Tavkar V Goyal A Kansal H Chopra V Garg K Sharma S . The interplay of ferroptosis and oxidative stress mechanisms: A critical contributor to chronic obstructive pulmonary disease pathophysiology. Metallomics. (2025) 17:mfaf030. doi: 10.1093/mtomcs/mfaf030 40795370

[21] Lee KY Yang CC Shueng PW Wu SM Chen CH Chao YC . Downregulation of TAZ elicits a mitochondrial redox imbalance and ferroptosis in lung epithelial cells exposed to diesel exhaust particles. Ecotoxicol Environ Saf. (2023) 266:115555. doi: 10.1016/j.ecoenv.2023.115555 37832483

[22] Qiao H Yang B Lv X Liu Y . Tetrachlorobisphenol A induces programmed cell death and senescence in vascular endothelial cells. Cell Biol Int. (2026) 50:e70121. doi: 10.1002/cbin.70121 41553760

[23] Xu X Pang Y Fan X . Mitochondria in oxidative stress, inflammation and aging: From mechanisms to therapeutic advances. Signal Transduct Target Ther. (2025) 10:190. doi: 10.1038/s41392-025-02253-4 40500258 PMC12159213

[24] Lebiedzinska-Arciszewska M Suski J Bonora M Pakula B Pinton P Duszynski J . The relation between mitochondrial membrane potential and reactive oxygen species formation. Methods Mol Biol. (2025) 2878:133–62. doi: 10.1007/978-1-0716-4264-1_8 39546261

[25] Xu M Feng P Yan J Li L . Mitochondrial quality control: A pathophysiological mechanism and potential therapeutic target for chronic obstructive pulmonary disease. Front Pharmacol. (2024) 15:1474310. doi: 10.3389/fphar.2024.1474310 39830343 PMC11739169

[26] Antunes MA Lopes-Pacheco M Rocco PRM . Oxidative stress-derived mitochondrial dysfunction in chronic obstructive pulmonary disease: A concise review. Oxid Med Cell Longev. (2021) 2021:6644002. doi: 10.1155/2021/6644002 37448755 PMC10337713

[27] Ni FX Chen HH Jiang ZB Huang DH . Ironing out COPD: Ferroptosis-driven immune dysregulation, metabolic rewiring, and precision therapeutic opportunities. Front Immunol. (2026) 17:1630969. doi: 10.3389/fimmu.2026.1630969 41836443 PMC12982077

[28] Li C Liu Q Chang Q Xie M Weng J Wang X . Role of mitochondrial fusion proteins MFN2 and OPA1 on lung cellular senescence in chronic obstructive pulmonary disease. Respir Res. (2023) 24:319. doi: 10.1186/s12931-023-02634-9 38110986 PMC10726594

[29] Antunes MA Braga CL Oliveira TB Kitoko JZ Castro LL Xisto DG . Mesenchymal stromal cells from emphysematous donors and their extracellular vesicles are unable to reverse cardiorespiratory dysfunction in experimental severe emphysema. Front Cell Dev Biol. (2021) 9:661385. doi: 10.3389/fcell.2021.661385 34136481 PMC8202416

[30] Cheng XG Liu YC Chen F Li JW Yao XZ Chen QY . GNPAT/USP30 stabilizes DRP1 protein to promote mitochondrial fission and functional damage in COPD progression. Kaohsiung J Med Sci. (2025) 41:e70080. doi: 10.1002/kjm2.70080 40709564 PMC12694566

[31] Wang Y Xia S . Relationship between ACSL4-mediated ferroptosis and chronic obstructive pulmonary disease. Int J Chron Obstruct Pulmon Dis. (2023) 18:99–111. doi: 10.2147/copd.S391129 36817367 PMC9930680

[32] Liu JY Zhang MY Qu YQ . The underlying role of mitophagy in different regulatory mechanisms of chronic obstructive pulmonary disease. Int J Chron Obstruct Pulmon Dis. (2020) 15:2167–77. doi: 10.2147/copd.S265728 32982209 PMC7501977

[33] Fang L Zhang M Li J Zhou L Tamm M Roth M . Airway smooth muscle cell mitochondria damage and mitophagy in COPD via ERK1/2 MAPK. Int J Mol Sci. (2022) 23:13987. doi: 10.3390/ijms232213987 36430467 PMC9694999

[34] Gao M Liang C Hong W Yu X Zhou Y Sun R . Biomass-related PM2.5 induces mitochondrial fragmentation and dysfunction in human airway epithelial cells. Environ pollut. (2022) 292:118464. doi: 10.1016/j.envpol.2021.118464 34763019

[35] Giordano L Gregory AD Pérez Verdaguer M Ware SA Harvey H DeVallance E . Extracellular release of mitochondrial DNA: Triggered by cigarette smoke and detected in COPD. Cells. (2022) 11:369. doi: 10.3390/cells11030369 35159179 PMC8834490

[36] Zou X Huang Q Kang T Shen S Cao C Wu J . An integrated investigation of mitochondrial genes in COPD reveals the causal effect of NDUFS2 by regulating pulmonary macrophages. Biol Direct. (2025) 20:4. doi: 10.1186/s13062-025-00593-3 39789601 PMC11715544

[37] Gong Z Chen Z Sang S Yang L Qin H Li Q . Mitochondrial DNA 6 mA methylation by METTL4 drives neuroinflammation via cGAS-STING activation in vascular cognitive impairment. Free Radic Biol Med. (2026) 246:1–16. doi: 10.1016/j.freeradbiomed.2026.01.019 41534568

[38] Wu J Pan C Zhu R Huang X Lou X Yang L . A heteroplasmic MT-CO2 m.8024G > A variant is associated with mitochondrial bioenergetic deficiency and optic atrophy. Mol Neurobiol. (2026) 63:485. doi: 10.1007/s12035-026-05774-3 41779224

[39] Li J Huang Y Chang R Ni A Fang L Zhou X . Developmental and metabolic toxicity of diphenyl phosphate: Insights from an integrative mechanistic framework in zebrafish embryos. Environ pollut. (2026) 392:127672. doi: 10.1016/j.envpol.2026.127672 41534654

[40] Wang Y Li P Cao Y Liu C Wang J Wu W . Skeletal muscle mitochondrial dysfunction in chronic obstructive pulmonary disease: Underlying mechanisms and physical therapy perspectives. Aging Dis. (2023) 14:33–45. doi: 10.14336/ad.2022.0603 36818563 PMC9937710

[41] Shamriz O Bar-On Z Yosef O Cohen-Daniel L Sheer A Reuven O . NDUFS4, a mitochondrial complex I subunit, is essential for T-cell metabolic fitness and immune function. Front Immunol. (2025) 16:1734203. doi: 10.3389/fimmu.2025.1734203 41573538 PMC12819820

[42] Ma Y Yan L Zhang Y Geng Y Yin X Gao R . Taurine restores oocyte quality by enhancing mitochondrial function in mice exposed to dibutyl phthalate during adolescence. Free Radic Biol Med. (2026) 247:224–39. doi: 10.1016/j.freeradbiomed.2026.01.046 41651302

[43] Wang J Tang X Lu Y Zheng Y Zeng F Shi W . Lycopene regulates dietary dityrosine-induced mitochondrial-lipid homeostasis by increasing mitochondrial complex activity. Mol Nutr Food Res. (2022) 66:e2100724. doi: 10.1002/mnfr.202100724 34780105

[44] Kumar U Jahnavi G Biswas B Alam B Varshney S . Molecular effect of tobacco on genetic, epigenetic, and metabolic pathways during cancer progression. Cureus. (2026) 18:e102757. doi: 10.7759/cureus.102757 41782786 PMC12954342

[45] Behan M Yen K Cohen P Kliment CR . Mitochondrial-derived microproteins in lung disease: Insights and implications. Am J Physiol Lung Cell Mol Physiol. (2026) 330:L222–l231. doi: 10.1152/ajplung.00369.2025 41569667 PMC12961588

[46] Mizumura K Ozoe R Nemoto Y Furusho N Kurosawa Y Kozu Y . Cigarette smoke extract-induced necroptosis causes mitochondrial DNA release and inflammation of bronchial epithelial cells. Int J Chron Obstruct Pulmon Dis. (2025) 20:2685–95. doi: 10.2147/copd.S523610 40765678 PMC12323784

[47] Qu J Zhang M Hu Y Yang G Zhang X Zhang W . Identifying the key mitochondria-related genes in COPD by integrating machine learning and bioinformatics analyses. Int J Genomics. (2025) 2025:7060748. doi: 10.1155/ijog/7060748 41127826 PMC12538651

[48] Lin QI Zhang CF Chen JY Guo ZK Wu SY Li HY . Targeting mitochondrial dysfunction with lncRNAs in a Wistar rat model of chronic obstructive pulmonary disease. In Vivo. (2023) 37:2543–54. doi: 10.21873/invivo.13362 37905633 PMC10621436

[49] Ware SA Kliment CR Giordano L Redding KM Rumsey WL Bates S . Cell-free DNA levels associate with COPD exacerbations and mortality. Respir Res. (2024) 25:42. doi: 10.1186/s12931-023-02658-1 38238743 PMC10797855

[50] Jindal D Chopra V Garg K Sharma S . Mitochondrial DNA-mediated cGAS-STING activation and its crosstalk with NLRP3 inflammasome in chronic obstructive pulmonary disease. Cytokine Growth Factor Rev. (2026) 90:12–25. doi: 10.1016/j.cytogfr.2026.05.001 42143499

[51] Wong M Martinez T Hua M Hendricks NG Talbot P . Unraveling the toxicological effects of hydroxyacetone─a reaction product in electronic cigarette aerosols. Chem Res Toxicol. (2026) 39:305–18. doi: 10.1021/acs.chemrestox.5c00358 41650339

[52] Li X Imanishi K Umeshita S Senoo Y Guerrero PA Silva DV . Preferential use of alkyl-acyl phosphatidylinositol for GPI biosynthesis and diagnostic potential of lipidomics for inherited GPI deficiencies. J Biol Chem. (2026) 302:111256. doi: 10.1016/j.jbc.2026.111256 41654138 PMC12969624

[53] Lin Q Zhang CF Guo JL Su JL Guo ZK Li HY . Involvement of NEAT1/PINK1-mediated mitophagy in chronic obstructive pulmonary disease induced by cigarette smoke or PM(2.5). Ann Transl Med. (2022) 10:277. doi: 10.21037/atm-22-542 35433942 PMC9011272

[54] Tuineau MN Herbert LM Medina HE Naik JS Resta TC Jernigan NL . Mitochondrial acid-sensing ion channel 1a deficiency induces mitochondrial dysfunction in pulmonary arterial smooth muscle cells. Am J Physiol Lung Cell Mol Physiol. (2026) 330:L390–l403. doi: 10.1152/ajplung.00324.2025 41740252 PMC13074392

[55] Onofre M Santos RT Rocha NN Caldeira DAF Silva JD Silva CMD . Mitochondrial transplantation from bone marrow mesenchymal stromal cells combined with sildenafil attenuated vascular remodeling and improved right ventricular dysfunction in experimental pulmonary arterial hypertension. Int J Mol Sci. (2026) 27:1761. doi: 10.3390/ijms27041761 41751897 PMC12940746

[56] Tan Z Zhao M Li J Li S Zhu S Yao X . Myostatin is involved in skeletal muscle dysfunction in chronic obstructive pulmonary disease via Drp-1 mediated abnormal mitochondrial division. Ann Transl Med. (2022) 10:162. doi: 10.21037/atm-22-377 35280400 PMC8908114

[57] Wang Z Deng M Xu W Li C Zheng Z Li J . DKK3 as a diagnostic marker and potential therapeutic target for sarcopenia in chronic obstructive pulmonary disease. Redox Biol. (2024) 78:103434. doi: 10.1016/j.redox.2024.103434 39571512 PMC11617289

[58] Huang H Duan M Wei J Liu Y Xu S Huang M . Fibroblast growth factor 8 (FGF8) induces mitochondrial remodeling in chondrocytes via ERK/AMPK signaling pathway. FASEB J. (2025) 39:e70501. doi: 10.1096/fj.202500186R 40162651

[59] Xu X Zhao Y Zhu Z Wen W Li X . Mitofusin-mediated mitochondrial fusion inhibits pseudorabies virus infection in porcine cells. Vet Sci. (2025) 12:368. doi: 10.3390/vetsci12040368 40284870 PMC12030837

[60] Distefano A Orlando L Partsinevelos K Longhitano L Emma R Caruso M . Comparative evaluation of cigarette smoke and a heated tobacco product on microglial toxicity, oxidative stress and inflammatory response. J Transl Med. (2024) 22:876. doi: 10.1186/s12967-024-05688-5 39350202 PMC11440907

[61] Stevens NE Loreti M Ramirez-Sanchez I Dos Reis FCG Sacco A Breen EC . Cigarette smoke exposure impairs early-stage recovery from lengthening contraction-induced muscle injury in male mice. Physiol Rep. (2024) 12:e70064. doi: 10.14814/phy2.70064 39328164 PMC11427903

[62] Wang Y Dai X Li H Jiang H Zhou J Zhang S . The role of mitochondrial dynamics in disease. MedComm. (2023) 4:e462. doi: 10.1002/mco2.462 38156294 PMC10753647

[63] Cherny SS Chowers M Obolski U . Bayesian network modeling of patterns of antibiotic cross-resistance by bacterial sample source. Commun Med (Lond). (2023) 3:61. doi: 10.1038/s43856-023-00289-7 37130943 PMC10154291

[64] Liang Y Fan S Jiang Y Ji T Chen R Xu Q . Elevated serum mitochondrial DNA levels were associated with the progression and mortality in idiopathic pulmonary fibrosis. Int Immunopharmacol. (2023) 123:110754. doi: 10.1016/j.intimp.2023.110754 37573686

[65] Borah S Mishra R Dey S Suchanti S Bhowmick NA Giri B . Prognostic value of circulating mitochondrial DNA in prostate cancer and underlying mechanism. Mitochondrion. (2023) 71:40–9. doi: 10.1016/j.mito.2023.05.005 37211294

[66] Zeiner S Wohlrab P Rosicky I Schukro RP Klein KU Wojta J . Circulating cell-free mitochondrial DNA as a novel biomarker for intra-amniotic infection in obstetrics: a pilot trial. J Clin Med. (2024) 13:4616. doi: 10.3390/jcm13164616 39200758 PMC11354521

[67] Fu W Wang J Lu N Guo Z Ong SB Gao Y . Unveiling the crucial nexus: mitochondrial quality control as a central driver in metabolic dysfunction-associated steatotic liver disease pathogenesis. Cell Prolif. (2026) 59:e70141. doi: 10.1111/cpr.70141 41207882 PMC12877942

[68] Xue K Yang J Hu J Kong L Cui X Kong Y . FGF9 drives mitochondrial biogenesis in glioblastoma by activating the CREB-PGC-1α axis. Peptides. (2026) 195:171463. doi: 10.1016/j.peptides.2025.171463 41485715

[69] Nguyen Huu T Duong Thanh H Kim MK Kumar Sah D Trinh VH Yoon HJ . 1,1-Diethoxyethane enhances aerobic respiration in human mitochondria via activation of AMP-activated protein kinase. Commun Biol. (2026) 9:361. doi: 10.1038/s42003-026-09797-3 41775972 PMC12982773

[70] Sideris D Lee H Olson L Nallaparaju K Okuyama K Ciavarri J . Suppression of interferon signaling via small-molecule modulation of TFAM. Elife. (2026) 14:RP108742. doi: 10.7554/eLife.108742 41649246 PMC12880803

[71] Tulen CBM van de Wetering C Schiffers CHJ Weltjens E Benedikter BJ Leermakers PA . Alterations in the molecular control of mitochondrial turnover in COPD lung and airway epithelial cells. Sci Rep. (2024) 14:4821. doi: 10.1038/s41598-024-55335-8 38413800 PMC10899608

[72] Benisty S Ben-Jacob E Ariel G Be'er A . Antibiotic-induced anomalous statistics of collective bacterial swarming. Phys Rev Lett. (2015) 114:18105. doi: 10.1103/PhysRevLett.114.018105 25615508

[73] Fan Z Luo P Gao Y Ma H Fan J Sanan S . Unraveling the role of mitochondrial dysfunction in diabetic kidney disease: insights and interventions. Front Pharmacol. (2025) 16:1618418. doi: 10.3389/fphar.2025.1618418 41567637 PMC12815871

[74] Li CL Liu JF Liu SF . Mitochondrial dysfunction in chronic obstructive pulmonary disease: unraveling the molecular nexus. Biomedicines. (2024) 12:814. doi: 10.3390/biomedicines12040814 38672169 PMC11048013

[75] Ren YJ Sun TQ Lu Y Liu DL Gao R Li T . rhCC16 suppresses cellular senescence and ameliorates COPD-like symptoms by activating the AMPK/Sirt1-PGC-1-α-TFAM pathway to promote mitochondrial function. J Cell Mol Med. (2025) 29:e70566. doi: 10.1111/jcmm.70566 40259209 PMC12011551

[76] Tew TB Yang CH . Astaxanthin protects retinal Müller cells against high glucose-induced oxidative stress through the sirtuin 1/AMPK/FOXO1 pathway. Exp Eye Res. (2026) 265:110887. doi: 10.1016/j.exer.2026.110887 41581827

[77] Zhu C Peng W Yang L Zhang W . High-intensity interval training alleviates COPD-induced gastrocnemius muscle dysfunction via the BRD4/PGC-1α axis through restoring mitochondrial function and oxidative fiber composition. J Muscle Res Cell Motil. (2026) 47:9. doi: 10.1007/s10974-026-09723-4 41865095 PMC13005851

[78] Ram J Glickman MH . The many faces of p97/Cdc48 in mitochondrial homeostasis. Essays Biochem. (2025) 69:EBC20253045. doi: 10.1042/ebc20253045 41498289 PMC12862955

[79] Shao N Yu H Li X Han M Chen C Zhu J . Ferritinophagy and organ injury. Autophagy. (2026) 22:1171–85. doi: 10.1080/15548627.2026.2633246 41692973 PMC13185467

[80] Han X Li P Jiang M Cao Y Wang Y Jiang L . Autophagy in skeletal muscle dysfunction of chronic obstructive pulmonary disease: implications, mechanisms, and perspectives. J Zhejiang Univ-Sci B. (2025) 26:227–39. doi: 10.1631/jzus.B2300680 40082202 PMC11906388

[81] Ito A Hashimoto M Tanihata J Matsubayashi S Sasaki R Fujimoto S . Involvement of Parkin-mediated mitophagy in the pathogenesis of chronic obstructive pulmonary disease-related sarcopenia. J Cachexia Sarcopenia Muscle. (2022) 13:1864–82. doi: 10.1002/jcsm.12988 35373498 PMC9178376

[82] Marchesan E Nardin A Mauri S Bernardo G Chander V Di Paola S . Activation of Ca(2+) phosphatase calcineurin regulates Parkin translocation to mitochondria and mitophagy in flies. Cell Death Differ. (2024) 31:217–38. doi: 10.1038/s41418-023-01251-9 38238520 PMC10850161

[83] Tian S Zhang Y Liu C Zhang H Lu Q Zhao Y . Double-edged mitophagy: balancing inflammation and resolution in lung disease. Clin Sci (Lond). (2025) 139:1047–72. doi: 10.1042/cs20256705 41026942 PMC12599259

[84] Ruan W Huang M Li X Peng Z Wei Y Mai Z . Regulated cell death in COPD: modulators, crosstalk mechanisms, and therapeutic opportunities. Eur J Pharmacol. (2026) 1015:178557. doi: 10.1016/j.ejphar.2026.178557 41544689

[85] Liao L Li J Xu W Yin Y Wang Z Li C . Calprotectin is a circulating biomarker and potential therapeutic target for sarcopenia in chronic obstructive pulmonary disease. J Cachexia Sarcopenia Muscle. (2026) 17:e70196. doi: 10.1002/jcsm.70196 41582634 PMC12833497

[86] Wei Y Zhou Q Li D He H Su Y Chang G . Geniposide attenuates post-myocardial infarction cardiac remodeling via Parkin-dependent suppression of hyperactivated mitophagy. Curr Med Chem. (2026). doi: 10.2174/0109298673402330251125051751 41572764

[87] Peng T Xie Y Sheng H Wang C Lian Y Xie N . Mitochondrial-derived vesicles: gatekeepers of mitochondrial response to oxidative stress. Free Radic Biol Med. (2022) 188:185–93. doi: 10.1016/j.freeradbiomed.2022.06.233 35750270

[88] Cheng Z . FoxO transcription factors in mitochondrial homeostasis. Biochem J. (2022) 479:525–36. doi: 10.1042/bcj20210777 35195252 PMC8883485

[89] Li JJ Wang YJ Wang CM Li YJ Yang Q Cai WY . Shenlian extract decreases mitochondrial autophagy to regulate mitochondrial function in microvascular to alleviate coronary artery no-reflow. Phytother Res. (2023) 37:1864–82. doi: 10.1002/ptr.7703 36740450

[90] Zhang M Fang L Zhou L Molino A Valentino MR Yang S . MAPK15-ULK1 signaling regulates mitophagy of airway epithelial cell in chronic obstructive pulmonary disease. Free Radic Biol Med. (2021) 172:541–9. doi: 10.1016/j.freeradbiomed.2021.07.004 34224814

[91] Cao W Cao G Meng Q Li D Zhang J Wu T . Integrative insights into mitochondrial dysfunction and organelle crosstalk in diabetic kidney disease. FASEB J. (2026) 40:e71692. doi: 10.1096/fj.202504735R 41863504

[92] Li MY Qin YQ Tian YG Li KC Oliver BG Liu XF . Effective-component compatibility of Bufei Yishen formula III ameliorated COPD by improving airway epithelial cell senescence by promoting mitophagy via the NRF2/PINK1 pathway. BMC Pulm Med. (2022) 22:434. doi: 10.1186/s12890-022-02191-9 36414945 PMC9682796

[93] Zheng X Chen H . Roles of autophagy in sepsis-induced myocardial dysfunction: a comprehensive review. Am J Transl Res. (2026) 18:64–76. doi: 10.62347/szyg2334 41676277 PMC12886153

[94] Xi W Liu F Zhong G Chen F Xie Y Lai M . Therapeutic effects of Melittin acupoint injection on rheumatoid arthritis through autophagy activation and PI3K/AKT/mTOR pathway inhibition. Quant Imaging Med Surg. (2026) 16:18. doi: 10.21037/qims-2025-540 41521996 PMC12780645

[95] Qiu YH Zhang TS Wang XW Wang MY Zhao WX Zhou HM . Mitochondria autophagy: a potential target for cancer therapy. J Drug Target. (2021) 29:576–91. doi: 10.1080/1061186x.2020.1867992 33554661

[96] Shen Y Chen L Chen J Qin J Wang T Wen F . Mitochondrial damage-associated molecular patterns in chronic obstructive pulmonary disease: pathogenetic mechanism and therapeutic target. J Transl Int Med. (2023) 11:330–40. doi: 10.2478/jtim-2022-0019 38130648 PMC10732348

[97] Hong E Mao J Ke Z Wu Y . Integrating multi-omics data to uncover causal links between mitochondria-related genes and chronic obstructive pulmonary disease: a Mendelian randomization study. Int J Chron Obstruct Pulmon Dis. (2026) 21:553092. doi: 10.2147/copd.S553092 41868721 PMC13003660

[98] Chen W Huang S He L Zhou X Li R Wang G . Immune-metabolic positive feedback model in COPD: cross-mechanisms and potential intervention strategies. Front Cell Dev Biol. (2026) 14:1756033. doi: 10.3389/fcell.2026.1756033 41869013 PMC13002790

[99] Wen B Ma B Tao H Chen S Zhang J Shi M . Polystyrene and polyvinyl chloride microplastics exposure induces ocular surface inflammation by causing mitochondrial damage and lipid metabolic disruption. J Hazard Mater. (2026) 502:140995. doi: 10.1016/j.jhazmat.2025.140995 41499868

[100] Zheng H Huo X Zhang W Lou S Li M Huang C . Selenium regulates pyroptosis through the ROS-mtDNA-cGAS-STING axis to alleviate trimethyltin chloride-induced inflammation in chicken kidneys. Comp Biochem Physiol C Toxicol Pharmacol. (2026) 302:110452. doi: 10.1016/j.cbpc.2026.110452 41525897

[101] Holley CL Schroder K . The rOX-stars of inflammation: links between the inflammasome and mitochondrial meltdown. Clin Transl Immunol. (2020) 9:e01109. doi: 10.1002/cti2.1109 32055400 PMC7008497

[102] Jassim AH Inman DM Mitchell CH . Crosstalk between dysfunctional mitochondria and inflammation in glaucomatous neurodegeneration. Front Pharmacol. (2021) 12:699623. doi: 10.3389/fphar.2021.699623 34366851 PMC8334009

[103] Ma Y Song R Duan C . Mitochondrial quality control and transfer communication in neurological disorders and neuroinflammation. Front Immunol. (2025) 16:1542369. doi: 10.3389/fimmu.2025.1542369 40356918 PMC12066325

[104] Hoolachan JM Balakrishnan R McCown EM Merz KE Zhou C Bloom-Saldana E . STX4 is indispensable for mitochondrial homeostasis in skeletal muscle. J Cachexia Sarcopenia Muscle. (2025) 16:e70113. doi: 10.1002/jcsm.70113 41214862 PMC12602274

[105] Li Y Lu J Chen Y Li Z Zhang Z Zheng H . The effect of anion inhalation in a mouse model of cigarette smoke-induced chronic obstructive pulmonary disease. Copd. (2026) 23:2603725. doi: 10.1080/15412555.2025.2603725 41649473

[106] Lee BW Ha JH Yi DH Kim JH Jeong SH Jeong JH . Boehmeria nivea (L.) Gaud. ameliorate oxidative stress-mediated inflammatory responses and apoptosis in LPS/CSC-induced chronic obstructive pulmonary disease mouse model. Front Pharmacol. (2025) 16:1710694. doi: 10.3389/fphar.2025.1710694 41684519 PMC12891129

[107] Suhagiya GH Shah SV Gondaliya KR Singh A Mangukiya G Saiyad H . Oxidative stress markers and atherogenic lipid alterations among chronic smokers. Bioinformation. (2025) 21:4917–21. doi: 10.6026/973206300214917 41907932 PMC13018467

[108] Fairley LH Das S Dharwal V Amorim N Hegarty KJ Wadhwa R . Mitochondria-targeted antioxidants as a therapeutic strategy for chronic obstructive pulmonary disease. Antioxidants (Basel). (2023) 12:973. doi: 10.3390/antiox12040973 37107348 PMC10135688

[109] Fan X Dong T Yan K Ci X Peng L . PM2.5 increases susceptibility to acute exacerbation of COPD via NOX4/Nrf2 redox imbalance-mediated mitophagy. Redox Biol. (2023) 59:102587. doi: 10.1016/j.redox.2022.102587 36608590 PMC9813701

[110] Wang X Murugesan P Zhang P Xu S Peng L Wang C . NADPH oxidase isoforms in COPD patients and acute cigarette smoke-exposed mice: induction of oxidative stress and lung inflammation. Antioxidants (Basel). (2022) 11:1539. doi: 10.3390/antiox11081539 36009258 PMC9405243

[111] Taniguchi A Tsuge M Miyahara N Tsukahara H . Reactive oxygen species and antioxidative defense in chronic obstructive pulmonary disease. Antioxidants (Basel). (2021) 10:1537. doi: 10.3390/antiox10101537 34679673 PMC8533053

[112] Kiyokawa H Hoshino Y Sakaguchi K Muro S Yodoi J . Redox regulation in aging lungs and therapeutic implications of antioxidants in COPD. Antioxidants (Basel). (2021) 10:1429. doi: 10.3390/antiox10091429 34573061 PMC8470212

[113] Cui Y Liu KWK Ip MSM Liang Y Mak JCW . Protective effect of selegiline on cigarette smoke-induced oxidative stress and inflammation in rat lungs *in vivo*. Ann Transl Med. (2020) 8:1418. doi: 10.21037/atm-20-2426 33313163 PMC7723576

[114] Ge S Li R Li Y Liu J Yao S Zhang R . ROMO1 is involved in airway mucus hypersecretion in COPD through the mitochondrial ROS-STAT6 pathway. Respir Res. (2026) 27:103. doi: 10.1186/s12931-026-03578-6 41673839 PMC12949505

[115] Rathor P Tiwari AK Patel RP Verma AK Singh SP Ch R . Brain lipidomics identifies mitochondrial redox dysfunction and metabolic trade-offs associated with Parkinson's disease-like pathology induced by nanoplastics exposure. Free Radic Biol Med. (2026) 249:526–47. doi: 10.1016/j.freeradbiomed.2026.03.023 41812834

[116] Stein CS Zhang X Witmer NH Pennington ER Hahn S Straub AC . Mitoregulin supports mitochondrial membrane integrity and protects against cardiac ischaemia-reperfusion injury. Cardiovasc Res. (2026) 122:379–96. doi: 10.1093/cvr/cvag011 41555203 PMC13019689

[117] Li J Wang W He L Zhou Q . C3orf33/MISO regulates mitochondrial homeostasis via mitophagy. Autophagy. (2026) 22:874–6. doi: 10.1080/15548627.2026.2621110 41568773 PMC13020858

[118] Wu C Fu Q Liu J Fu J Zhang B Zhou J . Mitochondrial metabolism restoration via tramiprosate suppresses mitochondrial ROS-driven foamy macrophage senescence post spinal cord injury. J Orthop Translat. (2026) 57:101049. doi: 10.1016/j.jot.2026.101049 41808881 PMC12969124

[119] Sun ZJ Shan GY Wan H Zhang YX Gao ZC Shi WN . Atrazine increases hepatic inflammation and injury via endoplasmic reticulum (ER) stress mediated excessive formation of mitochondria-associated membranes (MAMs) and activation of the cGAS-STING pathway. Environ pollut. (2026) 397:127959. doi: 10.1016/j.envpol.2026.127959 41833653

[120] Ren X Lu X Song Y Li M Shao T Guo M . HPCAL1-BNIP3 axis promotes mitophagy-ferroptosis feedback loop that exacerbates intestinal ischemia-reperfusion injury. Free Radic Biol Med. (2026) 245:223–36. doi: 10.1016/j.freeradbiomed.2025.12.054 41482082

[121] Wu H Xu S Chen Y Yuan Z Yao Y Hao J . Phenazine-1-carboxamide from Streptomyces suppresses Phytophthora nicotianae via CDC48-targeted mitochondrial disruption. Plant Cell Environ. (2026) 49:2295–310. doi: 10.1111/pce.70383 41531331

[122] Kim D Jin J Lee YR Kim DH Park SY Byun JK . SLC25A33-mediated mitochondrial DNA synthesis plays a critical role in the inflammatory response of M1 macrophages by contributing to mitochondrial ROS and VDAC oligomerization. Int J Biol Sci. (2025) 21:2935–53. doi: 10.7150/ijbs.96563 40384854 PMC12080393

[123] Rius-Pérez S Pérez S Toledano MB Sastre J . Mitochondrial reactive oxygen species and lytic programmed cell death in acute inflammation. Antioxid Redox Signal. (2023) 39:708–27. doi: 10.1089/ars.2022.0209 37450339 PMC10619893

[124] Shao M Chen J Zhang F Su Q Lin X Wang W . 4-octyl itaconate attenuates renal tubular injury in db/db mice by activating Nrf2 and promoting PGC-1α-mediated mitochondrial biogenesis. Ren Fail. (2024) 46:2403653. doi: 10.1080/0886022x.2024.2403653 39291665 PMC11411562

[125] Tabei Y Abe H Suzuki S Takeda N Arai JI Nakajima Y . Sedanolide activates KEAP1-NRF2 pathway and ameliorates hydrogen peroxide-induced apoptotic cell death. Int J Mol Sci. (2023) 24:16532. doi: 10.3390/ijms242216532 38003720 PMC10671709

[126] Chen L Wu K Shou C Zhang L Tu M Wang B . Bushen Huoxue decoction alleviates bisphenol a-induced infertility through the PMK-1 mitogen-activated protein kinases signaling pathway and downstream mitochondrial unfolded protein response in Caenorhabditis elegans. Front Pharmacol. (2025) 16:1713681. doi: 10.3389/fphar.2025.1713681 41625340 PMC12852476

[127] Cornell R Handley A Pocock R . Gas-sensing neurons prime mitochondrial fitness to offset metabolic stress. Proc Natl Acad Sci USA. (2026) 123:e2525619123. doi: 10.1073/pnas.2525619123 41779783 PMC12974494

[128] Zhang B Chang JY Lee MH Ju SH Yi HS Shong M . Mitochondrial stress and mitokines: therapeutic perspectives for the treatment of metabolic diseases. Diabetes Metab J. (2024) 48:1–18. doi: 10.4093/dmj.2023.0115 38173375 PMC10850273

[129] Kaštelan S Antunica AG Konjevoda S Tomić Z Sarić A Kulaš M . Mitochondrial ROS in retinal neurodegeneration: thresholds, quality control failure, and precision therapeutic windows. Biomolecules. (2026) 16:445. doi: 10.3390/biom16030445 41897380 PMC13024072

[130] Xie W Wu K Zhang L Feng X Yang S Jia S . The H(2)S donor sulforaphane inhibits NLRP(3) inflammasome activation by inducing mitochondrial autophagy and mitigating CBS-H(2)S axis damage in in-vitro and in-vivo models of Parkinson's disease. Bioorg Chem. (2026) 174:109708. doi: 10.1016/j.bioorg.2026.109708 41797134

[131] Chen Z Shi Q Wang C Zhang Z Yang J Luo W . Pharmacological basis of Codonopsis Radix in COPD: Lobetyolin modulates Nrf2/NF-κB-mediated inflammation and oxidative stress. J Ethnopharmacol. (2026) 363:121383. doi: 10.1016/j.jep.2026.121383 41698557

[132] Jiang M Li P Wang Y Cao Y Han X Jiang L . Role of Nrf2 and exercise in alleviating COPD-induced skeletal muscle dysfunction. Ther Adv Respir Dis. (2023) 17:17534666231208633. doi: 10.1177/17534666231208633 37966017 PMC10652666

[133] Barnes PJ . Oxidative stress in chronic obstructive pulmonary disease. Antioxidants (Basel). (2022) 11:965. doi: 10.3390/antiox11050965 35624831 PMC9138026

[134] Roger I Estornut C Montero P Aparici M Puig C Davis AM . Targeting the Keap1-Nrf2 axis in COPD: comparative analysis of electrophilic and peptide-based Nrf2 activators in airway and immune cells. Eur J Pharmacol. (2026) 1019:178716. doi: 10.1016/j.ejphar.2026.178716 41786067

[135] De Vita S Masullo M Grambone S Bescós PB Piacente S Bifulco G . Demethylcalabaxanthone from Garcinia mangostana exerts antioxidant effects through the activation of the Nrf2 pathway as assessed via molecular docking and biological evaluation. Antioxidants (Basel). (2023) 12:1980. doi: 10.3390/antiox12111980 38001833 PMC10669650

[136] Zi Y Wang X Zi Y Yu H Lan Y Fan Y . Cigarette smoke induces the ROS accumulation and iNOS activation through deactivation of Nrf-2/SIRT3 axis to mediate the human bronchial epithelium ferroptosis. Free Radic Biol Med. (2023) 200:73–86. doi: 10.1016/j.freeradbiomed.2023.03.002 36871899

[137] Roberson JK Bauer AN Lopez-Ramirez A Jenness DB Cruz Zayas S Cooke Bailey JN . Environmental exposures and oxidative stress in retinal and optic nerve diseases: mechanisms, consequences, and therapeutic opportunities. Antioxidants (Basel). (2026) 15:281. doi: 10.3390/antiox15030281 41897428 PMC13023984

[138] Elgazzar YA Mehanna MG Ibrahim SEE AbdelGhani AM Negm A Abou Dahab M . Evaluating the level of lead, chromium and malondialdehyde in a sample of hypertensive elderly tobacco smokers in urban and rural populations and impact of physical activity: a cross-sectional comparative study. Clin Ter. (2026) 177:333–40. doi: 10.7417/ct.2026.2013 41773374

[139] Hasan SK Jayakumar S Espina Barroso E Jha A Catalano G Sandur SK . Molecular targets of oxidative stress: focus on nuclear factor erythroid 2-related factor 2 function in leukemia and other cancers. Cells. (2025) 14:713. doi: 10.3390/cells14100713 40422216 PMC12110329

[140] Wang R Liang L Matsumoto M Iwata K Umemura A He F . Reactive oxygen species and NRF2 signaling, friends or foes in cancer? Biomolecules. (2023) 13:353. doi: 10.3390/biom13020353 36830722 PMC9953152

[141] Krekora J Pawlowska E Derwich M Drożdż J Blasiak J . Oxidative stress, mitochondrial homeostasis, and sirtuins in atrial fibrillation. Int J Mol Sci. (2025) 27:175. doi: 10.3390/ijms27010175 41516053 PMC12785412

[142] Zhou WC Qu J Xie SY Sun Y Yao HW . Mitochondrial dysfunction in chronic respiratory diseases: implications for the pathogenesis and potential therapeutics. Oxid Med Cell Longev. (2021) 2021:5188306. doi: 10.1155/2021/5188306 34354793 PMC8331273

[143] Jiang X Wang M Li H Liu Y Dong X . Identification of oxidative stress-associated biomarkers in chronic obstructive pulmonary disease: an integrated bioinformatics analysis. Int J Chron Obstruct Pulmon Dis. (2025) 20:841–55. doi: 10.2147/copd.S485505 40161396 PMC11955178

[144] Wang S Zhong M Deng X Liu C Tan Y Qian B . Based exploration of the diagnostic value of oxidative stress-related key genes in chronic obstructive pulmonary disease. Cell Biol Toxicol. (2025) 41:69. doi: 10.1007/s10565-025-10019-5 40214820 PMC11991958

[145] Torfs K Vermeersch G Gouwy M Devos T Proost P Struyf S . Neutrophils as critical orchestrators of chronic inflammation. Cell Mol Immunol. (2026) 23:123–49. doi: 10.1038/s41423-025-01380-w 41530536 PMC12858905

[146] Patel N Baydur A . Molecular and structural changes, and skeletal muscle strength and endurance in chronic obstructive pulmonary disease and interstitial lung disease: practical applications of assessment and management. Bioengineering (Basel). (2026) 13:329. doi: 10.3390/bioengineering13030329 41899860 PMC13024137

[147] Duggan E Fuqua JD Hagy B Georgescu C Miller BF Van Remmen H . Phospholipid glutathione peroxidase overexpression mitigates cancer cachexia by protecting muscle mass and lowering inflammation. J Cachexia Sarcopenia Muscle. (2026) 17:e70255. doi: 10.1002/jcsm.70255 41854231 PMC13140832

[148] Manolis AS Manolis AA Manolis TA Vouliotis A . Chronic obstructive pulmonary disease and cardiovascular disorders. Curr Vasc Pharmacol. (2026). doi: 10.2174/0115701611418539260124113737 41863468

[149] Ragnoli B Bertelegni C Brugiatelli L Giovanni T Chiazza F Malerba M . Chronic obstructive pulmonary disease as an independent predictor of left main coronary artery disease. Med Sci (Basel). (2026) 14:131. doi: 10.3390/medsci14010131 41892846 PMC13027644

[150] Nur Azan NI Abdul Karim N Sulaiman N Ng MH Najib AM Hassan H . Oxidative stress and mitochondrial dysfunction in cardiovascular aging: current insights and therapeutic advances. Biomedicines. (2026) 14:100. doi: 10.3390/biomedicines14010100 41595638 PMC12838834

[151] Carvajal K Arias-Chávez DJ Mailloux-Salinas P Bravo G Gómez-Viquez NL . Oxidative modifications in cardiac mitochondrial and Ca(2+) handling proteins in obesity and metabolic syndrome: Antioxidant alternatives. Handb Exp Pharmacol. (2026) 293:127–43. doi: 10.1007/164_2026_794 41718778

[152] Cao P Zhang C Hua DX Li MD Lv BB Fu L . Serum 8-hydroxy-2'-deoxyguanosine predicts severity and prognosis of patients with acute exacerbation of chronic obstructive pulmonary disease. Lung. (2022) 200:31–9. doi: 10.1007/s00408-021-00507-w 34982215

[153] Zinellu E Zinellu A Fois AG Pau MC Scano V Piras B . Oxidative stress biomarkers in chronic obstructive pulmonary disease exacerbations: A systematic review. Antioxidants (Basel). (2021) 10:710. doi: 10.3390/antiox10050710 33946941 PMC8146706

[154] Ji Y Wang Y Yu X Jin Y Zhao K Hu Y . Hepatic mitochondrial signaling as a systemic hub: Inter-organ communication networks in aging and aging-related diseases. Front Cell Dev Biol. (2026) 14:1745201. doi: 10.3389/fcell.2026.1745201 41768994 PMC12935980

[155] Sawant H Borthakur A . Disease-specific novel role of growth differentiation factor 15 in organ fibrosis. Int J Mol Sci. (2025) 26:5713. doi: 10.3390/ijms26125713 40565178 PMC12193308

[156] Rama K Jahagirdar V Sanyal AJ . Fibroblast growth factor 21: Mechanisms, therapeutic potential, and clinical translation in metabolic dysfunction. Drug Des Devel Ther. (2026) 20:560034. doi: 10.2147/dddt.S560034 42077586 PMC13135343

[157] Amado CA Martín-Audera P Agüero J Ferrer-Pargada D Josa Laorden B Boucle D . Alterations in circulating mitochondrial signals at hospital admission for COPD exacerbation. Chron Respir Dis. (2023) 20:14799731231220058. doi: 10.1177/14799731231220058 38112134 PMC10734331

[158] Jing R Si S Zhu S Tang X Jiang Z . Current advancements in the investigation of mitochondria-targeting organic sensitizers in cancer immunotherapy. Biomater Sci. (2025) 13:5582–604. doi: 10.1039/d5bm01193k 40910367

[159] Fu H Huang Q Xie J . Targeting regulated cell death pathways in COPD: Mechanisms and therapeutic strategies. Cells. (2025) 14:1874. doi: 10.3390/cells14231874 41369363 PMC12691304

[160] Saaoud F Xu K Lu Y Shao Y Han B Wang X . Chronic obstructive pulmonary disease reprograms the lung into an immune organ through trained immunity, cell death networks, and immune checkpoint dysregulation. Front Med (Lausanne). (2026) 13:1721780. doi: 10.3389/fmed.2026.1721780 41684927 PMC12891065

[161] Zhang Y Wang C Zhang P Zhang Q Cao Y Jiang Z . Mitochondria-associated programmed cell death in pancreatic β cell of T2DM. Apoptosis. (2026) 31:84. doi: 10.1007/s10495-026-02315-0 41784740

[162] Dadsena S King LE García-Sáez AJ . Apoptosis regulation at the mitochondria membrane level. Biochim Biophys Acta Biomembr. (2021) 1863:183716. doi: 10.1016/j.bbamem.2021.183716 34343535

[163] Sang M Li X Chen M Ren X Kang S Chang Z . Role of mitochondria-associated ER in apoptosis. Cell Biochem Funct. (2025) 43:e70105. doi: 10.1002/cbf.70105 40693348

[164] Kim H Lee DG . Naringin-generated ROS promotes mitochondria-mediated apoptosis in Candida albicans. IUBMB Life. (2021) 73:953–67. doi: 10.1002/iub.2476 33934490

[165] Zhuang Y Yao L Qi W . Molecular mechanisms of programmed cell death pathways in chronic obstructive pulmonary disease. J Cell Physiol. (2026) 241:e70177. doi: 10.1002/jcp.70177 42077197

[166] Deragon MA McCaig WD Truong PV Metz KR Carron KA Hughes KJ . Mitochondrial trafficking of MLKL, Bak/Bax, and Drp1 is mediated by RIP1 and ROS which leads to decreased mitochondrial membrane integrity during the hyperglycemic shift to necroptosis. Int J Mol Sci. (2023) 24:8609. doi: 10.3390/ijms24108609 37239951 PMC10218403

[167] Luo D Liu J Ya L Ruan C Ou S Wei Y . Cigarette smoke exposure triggers dendritic cell-derived exosome-mediated Th17 and Treg polarization through an autophagy- and necroptosis-associated SIRT1-dependent mechanism *in vitro*. Front Immunol. (2025) 16:1715736. doi: 10.3389/fimmu.2025.1715736 41567193 PMC12815758

[168] Wang Z Zhang R Dai D Zhang M Gao D Zhang J . Remimazolam mitigates cerebral ischemia/reperfusion injury by concurrently alleviating damage to the blood-brain barrier and RIP3/MLKL-driven necroptosis. Int Immunopharmacol. (2026) 172:116182. doi: 10.1016/j.intimp.2026.116182 41520561

[169] Solon M Ge N Hambro S Haller S Jiang J Baca M . ZBP1 and TRIF trigger lethal necroptosis in mice lacking caspase-8 and TNFR1. Cell Death Differ. (2024) 31:672–82. doi: 10.1038/s41418-024-01286-6 38548850 PMC11093969

[170] Wang P Zheng SY Jiang RL Wu HD Li YA Lu JL . Necroptosis signaling and mitochondrial dysfunction cross-talking facilitate cell death mediated by chelerythrine in glioma. Free Radic Biol Med. (2023) 202:76–96. doi: 10.1016/j.freeradbiomed.2023.03.021 36997101

[171] Zhang J Chen H Wang Y Zhou X Zippi M Fiorino S . Cell death and its interaction with mitochondrial dysfunction in pathogenesis of acute pancreatitis: A comprehensive review. Apoptosis. (2026) 31:66. doi: 10.1007/s10495-026-02288-0 41663764

[172] Pan L Fang S Kong F Ye S Xiong Y . Mitochondrial dysfunction-induced PANoptosis: Mechanisms, triggers, and disease implications. Mitochondrion. (2026) 88:102115. doi: 10.1016/j.mito.2026.102115 41592633

[173] Zhang W Li G Luo R Lei J Song Y Wang B . Cytosolic escape of mitochondrial DNA triggers cGAS-STING-NLRP3 axis-dependent nucleus pulposus cell pyroptosis. Exp Mol Med. (2022) 54:129–42. doi: 10.1038/s12276-022-00729-9 35145201 PMC8894389

[174] Jeong S Park S Lee D Heo G Lee Y Rhee SH . ATP mediates pyroptosis in the intestinal mucosal system during colitis. J Cell Physiol. (2025) 240:e70071. doi: 10.1002/jcp.70071 40686264 PMC12278297

[175] Qi T Xiao Z Peng Y Zhou Y Wang W Li C . Pyroptosis in ischemic stroke: Roles, mechanisms, and therapeutic strategies. Restor Neurol Neurosci. (2026), 9226028251410011. doi: 10.1177/09226028251410011 41533807

[176] Pei Z Zhang M Rong T Lv L Ye G . Fucoxanthin regulates macrophage pyroptosis through the PI3K/AKT/mTOR signalling pathway. Mol Biol Rep. (2026) 53:250. doi: 10.1007/s11033-025-11408-z 41483374

[177] Li YP Niu XJ Zhang G Chan OK Sun N Hu B . Anti-alcoholism drug disulfiram inhibits PANoptosis by blocking mitochondrial permeabilization in macrophages. Front Immunol. (2025) 16:1726408. doi: 10.3389/fimmu.2025.1726408 41488643 PMC12757373

[178] Miao R Jiang C Chang WY Zhang H An J Ho F . Gasdermin D permeabilization of mitochondrial inner and outer membranes accelerates and enhances pyroptosis. Immunity. (2023) 56:2523–2541.e8. doi: 10.1016/j.immuni.2023.10.004 37924812 PMC10872579

[179] Chen TT Wang MY Kang JY Ou GC Wang R Zhang DW . K48 and K63 linkage-competed ubiquitination of BECN1 promotes circPDE4D-mediated autophagy in chronic obstructive pulmonary disease. Cell Death Dis. (2026) 17:321. doi: 10.1038/s41419-026-08582-8 41851080 PMC13039329

[180] Yu W Cui Y Li S Luo L . TRAF6 promotes the ferroptosis defense through AKT/mitochondria damage in KRAS-driven lung cancer. Biochem Pharmacol. (2026) 246:117746. doi: 10.1016/j.bcp.2026.117746 41580038

[181] Ali MY Oliva CR Flor S Griguer CE . Mitoferrin, cellular and mitochondrial iron homeostasis. Cells. (2022) 11:3464. doi: 10.3390/cells11213464 36359860 PMC9658796

[182] Liu ZH Zhai Y Zhang J Huang W Li W Qin W . Mitochondrial iron deficiency mediated inhibition of ecdysone synthesis underlies lead (Pb) induced developmental toxicity in Drosophila melanogaster. Toxicol Appl Pharmacol. (2025) 497:117283. doi: 10.1016/j.taap.2025.117283 40020975

[183] Ning J Wen L Qiao L . Ferritinophagy: Molecular mechanisms and its crosstalk with ferroptosis in chronic respiratory diseases. Cell Biol Toxicol. (2026) 42:31. doi: 10.1007/s10565-026-10150-x 41629496 PMC12894163

[184] Anandhan A Dodson M Shakya A Chen J Liu P Wei Y . NRF2 controls iron homeostasis and ferroptosis through HERC2 and VAMP8. Sci Adv. (2023) 9:eade9585. doi: 10.1126/sciadv.ade9585 36724221 PMC9891695

[185] Blumenfeld H Besaratinia A Tommasi S . Long noncoding RNAs at the crossroads of smoking, oxidative stress, inflammation, and lung disease. Arch Toxicol. (2026) 100:1789–823. doi: 10.1007/s00204-026-04348-5 41838062

[186] Javadov S . Mitochondria and ferroptosis. Curr Opin Physiol. (2022) 25:100483. doi: 10.1016/j.cophys.2022.100483 35342847 PMC8944045

[187] Chen F Liu J Tang D Kang R . Monitoring mitochondria function in ferroptosis. Methods Mol Biol. (2023) 2712:103–15. doi: 10.1007/978-1-0716-3433-2_10 37578700

[188] Liu Y Liu S Tomar A Yen FS Unlu G Ropek N . Autoregulatory control of mitochondrial glutathione homeostasis. Science. (2023) 382:820–8. doi: 10.1126/science.adf4154 37917749 PMC11170550

[189] Huang R Xu R Shi J Yang Z Zheng J Wei D . Artesunate induces ferroptosis in osteosarcoma through NCOA4-mediated ferritinophagy. FASEB J. (2025) 39:e70488. doi: 10.1096/fj.202403160R 40168090 PMC11960798

[190] Chu C Wang X Yang C Chen F Shi L Xu W . Neutrophil extracellular traps drive intestinal microvascular endothelial ferroptosis by impairing Fundc1-dependent mitophagy. Redox Biol. (2023) 67:102906. doi: 10.1016/j.redox.2023.102906 37812880 PMC10579540

[191] Dai Q Wei X Zhao J Zhang D Luo Y Yang Y . Inhibition of FSP1: A new strategy for the treatment of tumors (Review). Oncol Rep. (2024) 52:105. doi: 10.3892/or.2024.8764 38940330 PMC11228423

[192] Zhang R Kang R Tang D . Gut microbiome mediates ferroptosis resistance for colorectal cancer development. Cancer Res. (2024) 84:796–7. doi: 10.1158/0008-5472.Can-24-0275 38276975

[193] Zeng Z Li T Liu X Ma Y Luo L Wang Z . DNA dioxygenases TET2 deficiency promotes cigarette smoke induced chronic obstructive pulmonary disease by inducing ferroptosis of lung epithelial cell. Redox Biol. (2023) 67:102916. doi: 10.1016/j.redox.2023.102916 37812881 PMC10579541

[194] Bucarey JL Trujillo-González I Paules EM Espinosa A . Myokines and their potential protective role against oxidative stress in metabolic dysfunction-associated steatotic liver disease (MASLD). Antioxidants (Basel). (2024) 13:1363. doi: 10.3390/antiox13111363 39594505 PMC11591161

[195] Ayantoye JO Yang B Zhang H Dong J Zhang X Song H . Ferroptosis-driven cryoinjury in porcine testicular tissue: Mechanisms, antioxidant-based cryoprotection, and translational strategies for fertility preservation. Anim Reprod Sci. (2026) 287:108113. doi: 10.1016/j.anireprosci.2026.108113 41576876

[196] Zhang Y Jiang Y Li Y Wu F Chen G Lu Z . SLC7A11 alleviates diquat-induced neurotoxicity by inhibiting ferroptosis and preserving mitochondrial function. Ecotoxicol Environ Saf. (2026) 309:119694. doi: 10.1016/j.ecoenv.2026.119694 41518980

[197] Sui J Johnson AR Kapellos TS Shiva S Kliment CR . ANT1 deficiency impairs macrophage metabolism and migration, protecting against emphysema in chronic obstructive pulmonary disease. Am J Respir Cell Mol Biol. (2025) 73:725–40. doi: 10.1165/rcmb.2024-0469OC 40439531 PMC12618869

[198] Hassibi S Donnelly LE . Macrophage dysfunction in respiratory disease. Results Probl Cell Differ. (2024) 74:239–56. doi: 10.1007/978-3-031-65944-7_9 39406908

[199] Sevilla-Montero J Munar-Rubert O Pino-Fadón J Aguilar-Latorre C Villegas-Esguevillas M Climent B . Cigarette smoke induces pulmonary arterial dysfunction through an imbalance in the redox status of the soluble guanylyl cyclase. Free Radic Biol Med. (2022) 193:9–22. doi: 10.1016/j.freeradbiomed.2022.09.026 36174878

[200] Yang S Guan Y Yu Q Zheng C Xia X Ma X . Targeting miR-499-5p for neuroprotection in spinal cord injury: Implications for inflammation and ROS-induced neuronal damage. Exp Cell Res. (2026) 457:114926. doi: 10.1016/j.yexcr.2026.114926 41638387

[201] Wagatsuma T Yamamoto A Nishimura Y Kawami M Ito J Hirayama T . Zinc-MTF1-metallothionein axis plays critical roles in the defense against ferroptosis in human cells. Free Radic Biol Med. (2026) 245:390–404. doi: 10.1016/j.freeradbiomed.2026.01.008 41500342

[202] Papolos DF Teicher MH Post RM . Treatment of early-onset specified and unspecified bipolar disorders: A systematic review and strategies for identifying and managing a thermally dysregulated subtype in children. Acta Psychiatr Scand. (2025) 152:156–79. doi: 10.1111/acps.13817 40844121 PMC12318650

[203] Xia J Jiang S Dong S Liao Y Zhou Y . The role of post-translational modifications in regulation of NLRP3 inflammasome activation. Int J Mol Sci. (2023) 24:6126. doi: 10.3390/ijms24076126 37047097 PMC10093848

[204] Sahoo K Sharma A . Understanding the mechanistic roles of environmental heavy metal stressors in regulating ferroptosis: adding new paradigms to the links with diseases. Apoptosis. (2023) 28:277–92. doi: 10.1007/s10495-022-01806-0 36611106

[205] Rizzotto D Zierhut C Villunger A . Mitotic errors as triggers of cell death and inflammation. Nat Cell Biol. (2026) 28:21–34. doi: 10.1038/s41556-025-01785-9 41495201

[206] Li MY Song GH Ren MY . Research progress in molecular mechanism of programmed cell death in bronchial asthma and traditional Chinese medicine intervention. Zhongguo Zhong Yao Za Zhi. (2025) 50:6807–15. doi: 10.19540/j.cnki.cjcmm.20250725.707 41814689

[207] Bucarey JL Casas M Espinosa A . Mitochondrial iron handling and lipid peroxidation as drivers of ferroptosis. Int J Mol Sci. (2026) 27:2232. doi: 10.3390/ijms27052232 41828457 PMC12984849

[208] Zhu J Lv L Yan Y Wang S Lu X Ma X . Role of autophagy in tumorigenesis and drug resistance: molecular mechanisms and therapeutic targets. Mol BioMed. (2026) 7:28. doi: 10.1186/s43556-026-00430-7 41814065 PMC12979803

[209] Perera R Kim SL Kim JH Kim KC Lee DS . Isopimaric acid derived from Torreya nucifera blocks autophagy and mitophagy to sensitize colon cancer cells to nutrient starvation. Cell Stress Chaperones. (2026) 31:100151. doi: 10.1016/j.cstres.2026.100151 41679689 PMC13080634

[210] Chen W Gullett JM Tweedell RE Kanneganti TD . Innate immune inflammatory cell death: PANoptosis and PANoptosomes in host defense and disease. Eur J Immunol. (2023) 53:e2250235. doi: 10.1002/eji.202250235 36782083 PMC10423303

[211] Shi R Liang R Wang F Wang L Zidai W Zhang J . Identification and experimental validation of PYCARD as a crucial PANoptosis-related gene for immune response and inflammation in COPD. Apoptosis. (2024) 29:2091–107. doi: 10.1007/s10495-024-01961-6 38652339

[212] Nguyen TT Wei S Nguyen TH Jo Y Zhang Y Park W . Mitochondria-associated programmed cell death as a therapeutic target for age-related disease. Exp Mol Med. (2023) 55:1595–619. doi: 10.1038/s12276-023-01046-5 37612409 PMC10474116

[213] Somayajulu M Wright R Muhammed FS McClellan SA Ibrahim AS Hazlett LD . PM(10) disrupts mitochondrial homeostasis in corneal epithelial cells: Protective effects of SKQ1. Antioxidants (Basel). (2026) 15:284. doi: 10.3390/antiox15030284 41897431 PMC13023551

[214] Lin Z Zhou E Zhang D Ke J Wang Y Zou X . HA/CD44-SS31 mitochondrial targeting of manganese oxide nanozymes for ischemia-reperfusion-induced acute kidney injury therapy. ACS Nano. (2026) 20:4866–90. doi: 10.1021/acsnano.5c16933 41649374

[215] Li Y Li M Wang H Chen X Luan H Lv J . Mitoquinone mesylate enhances bovine oocyte *in vitro* maturation efficiency by modulating oxidative stress and enhancing mitochondrial function. Theriogenology. (2026) 253:117798. doi: 10.1016/j.theriogenology.2025.117798 41481972

[216] Cheng D Yang S Wang C Fan K Gao F Sun Q . Reactive oxygen species in fetal growth restriction mechanisms and therapeutic directions (review). Int J Mol Med. (2026) 57:121. doi: 10.3892/ijmm.2026.5792 41823521 PMC12987557

[217] Lores-Arnaiz S . New insights on mitochondria-targeted neurological drugs. Biol (Basel). (2026) 15:228. doi: 10.3390/biology15030228 41677699 PMC12896874

[218] Wu R Wu Y . SKQ1: a mitochondria-targeted antioxidant with therapeutic potential. J Drug Target. (2026) 14:1–14. doi: 10.1080/1061186x.2026.2613054 41493196

[219] Jiang Y Huang Z Zhou T Wu M Zhao J Xiong Z . Mitochondria-target ubiquinone attenuates bleomycin-induced pulmonary fibrosis. Front Pharmacol. (2025) 16:1661644. doi: 10.3389/fphar.2025.1661644 40978478 PMC12443766

[220] Ghanem R Youf R Haute T Buin X Riool M Pourchez J . The (re)emergence of aerosol delivery: Treatment of pulmonary diseases and its clinical challenges. J Control Release. (2025) 379:421–39. doi: 10.1016/j.jconrel.2025.01.017 39800241

[221] Liu H Huang S Chen S Liang S Huang M Li S . Pharmacological potential of Chinese botanical drugs in managing chronic kidney disease by targeting mitochondrial quality control. Front Pharmacol. (2025) 16:1725842. doi: 10.3389/fphar.2025.1725842 41799382 PMC12960602

[222] Gutiérrez S Ospina C Cáceres T Patiño LH Paniz-Mondolfi A Ramírez JD . Transcriptomic and ultrastructural responses to amiodarone-itraconazole in naturally benznidazole-resistant and -susceptible Trypanosoma cruzi strains. PloS NeglTrop Dis. (2026) 20:e0013916. doi: 10.1371/journal.pntd.0013916 41533740 PMC12863684

[223] Furukawa K Maruyama T Sakai Y Noda NN Kanki T . Mfi2: an outer mitochondrial membrane mitofissin required for mitophagy. Autophagy Rep. (2026) 5:2635914. doi: 10.1080/27694127.2026.2635914 41789122 PMC12959179

[224] Li Y Zhong W Li L Zhang F Duan X Si H . Mitochondrial quality control modulating chondrocyte behavior and fate in knee osteoarthritis: mechanistic insights and therapeutic prospects. Front Immunol. (2026) 17:1671502. doi: 10.3389/fimmu.2026.1671502 41822484 PMC12975607

[225] Hsu CN Tain YL . Developmental programming of kidney disease across the life course: A narrative review focused on inflammation. Int J Mol Sci. (2026) 27:2244. doi: 10.3390/ijms27052244 41828468 PMC12984725

[226] Alia S Pedriali G Compagnucci P Valeri Y Membrino V Di Crescenzo T . Mitochondria at the crossroads of cardiovascular disease: Mechanistic drivers and emerging therapeutic strategies. Cells. (2026) 15:372. doi: 10.3390/cells15040372 41744815 PMC12938911

[227] Xu Z Wang K Hu H Chen Y Qiu Y Cai J . Protective effects of Exocarpium Citri Grandis against sepsis-induced acute lung injury via PANoptosis inhibition. Front Nutr. (2025) 12:1661404. doi: 10.3389/fnut.2025.1661404 41496909 PMC12766971

[228] Cui Y Wang X Lu C Ran L Guo Z Yao J . Treating acute respiratory distress syndrome with a multifaceted nanomedicine: Inhibition of PANoptosis and enhancement of lung barrier integrity. Mater Today Bio. (2026) 36:102748. doi: 10.1016/j.mtbio.2025.102748 41560832 PMC12813356

[229] Shinkai R Mazaki Y Hachimori S Hashikura H Miwa S Horinouchi T . Cigarette smoke extract of heated tobacco product induces necroptosis via receptor-interacting protein 1-mediated phosphorylation of mixed-lineage kinase domain-like protein in vascular smooth muscle cells. Biol Pharm Bull. (2026) 49:520–4. doi: 10.1248/bpb.b25-00718 41850815

[230] Guo Y Wang Y Wang B Peng S Li N Zhang D . Ultrasound-responsive renal-targeted nanoparticles deliver TAK-242 to inhibit NF-κB/NLRP3 signaling and attenuate sepsis-associated acute kidney injury. Biomaterials. (2026) 329:123922. doi: 10.1016/j.biomaterials.2025.123922 41481962

[231] Liu X Fu J Yang R Zhou H Li P Chen J . A high-throughput reporter assay for screening potential gasdermin D inhibitors from natural products. Int J Biol Macromol. (2026) 339:150110. doi: 10.1016/j.ijbiomac.2026.150110 41500275

[232] Li D Tian Y Tian L Yu H Zhang L Wang S . Free fatty acids and endotoxins synergically induce pyroptosis in bovine hepatocytes. Metabolites. (2026) 16:53. doi: 10.3390/metabo16010053 41590661 PMC12844290

[233] Wang K Ji H Gao J Wang Z Gao Y Lian X . DDIT3 drives nucleus pulposus cell PANoptosis and intervertebral disc degeneration progression. Apoptosis. (2026) 31:25. doi: 10.1007/s10495-025-02231-9 41518406

[234] Solis FJ Gonzalez LM . A discrete nonlinear model for HPV immune suppression and evasion. Math Biosci. (2026) 393:109622. doi: 10.1016/j.mbs.2026.109622 41529740

[235] Ahmed OA Ghanem AS Móré M Nagy AC . Respiratory and related comorbidities' role in the risk of acute sinusitis: A 15-year longitudinal clinical study. J Clin Med. (2026) 15:660. doi: 10.3390/jcm15020660 41598600 PMC12841780

[236] Song C Liu Z Tang J Huang Y . Ferroptosis in cerebral ischemia/reperfusion injury: Mechanistic drivers and therapeutic frontiers. Neuropsychiatr Dis Treat. (2026) 22:580170. doi: 10.2147/ndt.S580170 41738060 PMC12927810

[237] Lin D Liu T Hang X Yu X Liu X Xu Y . Ferroptosis-centered mitochondria-ROS loop drives PFOS-induced renal dysfunction, from epidemiological evidence to mechanistic insights with mice model. J Agric Food Chem. (2026) 74:7110–24. doi: 10.1021/acs.jafc.5c14271 41716066 PMC12965318

[238] Timuroglu A Ozden ES Selcuk E Sarman E Oguzlar FC Kolay O . β-Hydroxy-β-methylbutyrate attenuates sepsis-associated lung injury by regulating NF-κB p65-mediated inflammation, ER stress and mitochondrial apoptosis in a rat model. Naunyn Schmiedebergs Arch Pharmacol. (2026) 399:11545–55. doi: 10.1007/s00210-026-05114-1 41711841 PMC13269544

[239] He Q Yang P Wang Y Xu W Feng Y Xie F . Effects of antioxidant nutrients on muscle mass, strength and function in COPD patients: A meta-analysis of randomized controlled trials. PloS One. (2025) 20:e0316842. doi: 10.1371/journal.pone.0316842 39823472 PMC11741611

[240] Mannino D D'Ariano M Bello I Paterniti I Casili G Panza E . Molecular insights into NLRP3 inflammasome and miRNA modulation in oral cancer. Front Pharmacol. (2025) 16:1713259. doi: 10.3389/fphar.2025.1713259 41560727 PMC12812990

[241] Gaire BP Koronyo Y Vit JP Hutton A Subedi L Fuchs DT . Identification of Chlamydia pneumoniae and NLRP3 inflammasome activation in Alzheimer's disease retina. Nat Commun. (2026) 17:771. doi: 10.1038/s41467-026-68580-4 41571675 PMC12827417

[242] Amado CA Martín-Audera P Agüero J Lavín BA Guerra AR Muñoz P . Associations between serum mitokine levels and outcomes in stable COPD: an observational prospective study. Sci Rep. (2022) 12:17315. doi: 10.1038/s41598-022-21757-5 36243733 PMC9569360

[243] Chang M Zhou Q Guan Y Zhao Y Guo S Qu Q . Activation of mitophagy by kinetin mitigates coal-silica mixed dust-induced pulmonary fibrosis via modulating macrophage mitochondrial function in mice. Antioxid Redox Signal. (2026) 44:528–49. doi: 10.1177/15230864251411565 41894156

[244] Liu D Li T Chu Q Zhu J Xia DD Li X . Oxygen physiology and mechanisms of oxygen toxicity: a narrative review. Med Gas Res. (2026). doi: 10.4103/mgr.MEDGASRES-D-25-00140 41575053 PMC13456449

[245] Feng Y Chen DZ Cai H Zhang QY . Dexmedetomidine attenuates hyperoxia-induced lung injury in BPD mice via regulation of the mitophagy-NLRP3-pyroptosis pathway. Arch Biochem Biophys. (2026) 778:110748. doi: 10.1016/j.abb.2026.110748 41581638

[246] Diomedi M Fattorini F Aliberti S Bianchessi LM Castellucci A Schmidt J . Idiopathic inflammatory myopathies: one year in review 2025. Clin Exp Rheumatol. (2026) 44:167–77. doi: 10.55563/clinexprheumatol/2z01e0 41738251

[247] Liu Y Hu Y Se R Yang Z Liu X Yu J . Nerandomilast attenuates idiopathic inflammatory myopathy-associated interstitial lung disease via inhibiting proliferation and differentiation of B cells. Front Immunol. (2026) 17:1771007. doi: 10.3389/fimmu.2026.1771007 41789097 PMC12956678

[248] Altan I Alasan F Dikis OS Turkis FC . Clinical, functional, and psychosocial profiles of chronic obstructive pulmonary disease (COPD) etiotypes: A taxonomy-based analysis. Med (Kaunas). (2026) 62:348. doi: 10.3390/medicina62020348 41752747 PMC12941940

[249] Liu H Chen AG . Integrating lung function parameters into the Framingham score for improved CVD risk prediction in COPD: A cross-sectional study based on NHANES 2007-2012. Copd. (2026) 23:2628586. doi: 10.1080/15412555.2026.2628586 41774056

[250] Han Z She Y Wu D Zhang N Liu Z Wang Z . Senescent hepatic stellate cells drive inflammation and disease progression in MASH (review). Exp Ther Med. (2026) 31:95. doi: 10.3892/etm.2026.13090 41694108 PMC12902812

[251] Haji G Wiegman CH Michaeloudes C Patel MS Curtis K Bhavsar P . Mitochondrial dysfunction in airways and quadriceps muscle of patients with chronic obstructive pulmonary disease. Respir Res. (2020) 21:262. doi: 10.1186/s12931-020-01527-5 33046036 PMC7552476

[252] Lagrange B Benmohamed F Eon-Bertho A Malleret L Trecourt A Glehen O . Loss of cPLA2α function attenuates inflammation and epithelial thickening in a mouse model of Haemophilus influenzae-mediated COPD exacerbation. Curr Res Microb Sci. (2026) 10:100556. doi: 10.1016/j.crmicr.2026.100556 41675617 PMC12887665

[253] Wu M Quan C Yao L Yang Y Liu J Zhu Q . The therapeutic effect and mechanism of vismodegib on COPD: Focusing on NETs and macrophage polarization. Chem Biol Drug Des. (2026) 107:e70258. doi: 10.1111/cbdd.70258 41637659

[254] Alsafadi HN Nybom A Wagner D Malmström A Lindstedt S Bjermer L . Change in the chondroitin/dermatan structure in distal lung tissue from COPD patients. Sci Rep. (2026) 16:9721. doi: 10.1038/s41598-026-44120-4 41872350 PMC13013573

[255] Lv D Yu Y . Copper-induced cell death in renal diseases: Molecular mechanisms and therapeutic implications. Drug Des Devel Ther. (2025) 19:11849–61. doi: 10.2147/dddt.S562664 41497347 PMC12766471

[256] Zhang Y Wei H Nouws J Jiang W Brewster RM Nguyen JP . Aberrant cellular communities underlying disease heterogeneity in chronic obstructive pulmonary disease. Nat Genet. (2026) 58:376–91. doi: 10.1038/s41588-025-02480-z 41578022 PMC12900648

[257] Oliva CR Flor S Griguer CE . Cytochrome c oxidase subunit COX4-1 reprograms erastin-induced cell death from ferroptosis to apoptosis: A transmitochondrial study. Antioxidants (Basel). (2025) 15:40. doi: 10.3390/antiox15010040 41596099 PMC12837571

[258] Pang E Zou Y Lu K Li J Chen X Zhu Y . Mitochondrial ROS dyshomeostasis: a key driver of accelerated supraspinatus atrophy after rotator cuff injury. Front Physiol. (2026) 17:1783596. doi: 10.3389/fphys.2026.1783596 41907529 PMC13017390

[259] Zhou MY Gao ZY Sun J Long WC Zhou J Fang WJ . Aquaporin-1 stabilizes β-catenin to promote NLRP3 inflammasome-mediated pyroptosis in rheumatoid arthritis. Apoptosis. (2026) 31:74. doi: 10.1007/s10495-026-02289-z 41718903

[260] Wang Z Chen G Liu Z . Integrated regulation of ferroptosis in prostate cancer covering mechanisms, resistance, and translational opportunities. J Mol Med (Berl). (2026) 104:40. doi: 10.1007/s00109-026-02641-5 41639281

[261] Jung YJ Choi H Lee SM Oh E . Montelukast modulates MPTP-induced ferroptosis and neuroinflammation linked to the GPX4/ACSL4/5-LOX pathway. Neuromolecular Med. (2026) 28:11. doi: 10.1007/s12017-025-08895-5 41712018

[262] Gorgori-Gonzalez A Soto-Rodriguez S Tamayo-Torres E Garcia-Dominguez E Sebastia V Gambini J . Leveraging mitochondrial stress to improve healthy aging. Sports Med Health Sci. (2026) 8:23–33. doi: 10.1016/j.smhs.2025.10.003 41646179 PMC12869046

[263] Chaudhari M Makhloufi J Doelling B Kataria R Bhatnagar A Kalra D . Structural and metabolic remodeling of skeletal muscle in heart failure with reduced ejection fraction: A review: Beyond the failing heart. Int J Mol Sci. (2026) 27:2886. doi: 10.3390/ijms27062886 41898746 PMC13026253

[264] Li X Ke G Hu Y Chen M . A tri-omics and machine learning framework identifies prognostic biomarkers and metabolic signatures in sepsis. Sci Rep. (2026) 16:6648. doi: 10.1038/s41598-026-37342-z 41611834 PMC12914058

[265] Luo L Wang T Peng T Gao C Shu Y Yang H . The pcMPO-AF rule for predicting postoperative atrial fibrillation after coronary artery bypass grafting. Sci Rep. (2026) 16:2862. doi: 10.1038/s41598-025-32318-x 41565722 PMC12828024

[266] Hu J Liu S Liu L Geng X Yang Q Chen Q . Neutrophil-specific transcriptomic profiling reveals a novel signature for active tuberculosis diagnosis. Microbiol Spectr. (2026) 14(4):e0191525. doi: 10.1128/spectrum.01915-25 41817214 PMC13055310

[267] Zha X Gong WW Li XY Xia Q Ruan JH Tan R . Integrative multi-omics analysis of Buti Huatan Tang in chronic obstructive pulmonary disease. J Vis Exp. (2026). doi: 10.3791/70383 41911070

[268] Yang K Wang J Ma Y Zhang H . Advances in traditional Chinese medicine for chronic obstructive pulmonary disease through multi-omics approaches. Front Cell Dev Biol. (2026) 14:1761374. doi: 10.3389/fcell.2026.1761374 41743790 PMC12929911

[269] Alahmari MA . Systems biology and multi-omics in asthma and COPD: A systematic review of computational approaches (2010-2024). J Asthma Allergy. (2026) 19:575312. doi: 10.2147/jaa.S575312 41878747 PMC13007689

[270] Sheridan ME Ahmed D Al Daraawi M Kovac N Cassol E . Mitochondrial electron transport chain modulation orchestrates divergent TLR3 and TLR7 responses. J Leukoc Biol. (2026) 118:qiag034. doi: 10.1093/jleuko/qiag034 41811758

[271] Bell AJ Ram S Labaki WW Murray S Kazerooni EA Galban S . Temporal exploration of chronic obstructive pulmonary disease phenotypes: Insights from the COPDGene and SPIROMICS cohorts. Am J Respir Crit Care Med. (2025) 211:569–76. doi: 10.1164/rccm.202401-0127OC 39269427 PMC12005034

[272] Deng X Wu J He M Mei L Ma L Lin Y . Unveiling cholesterol metabolism-related gene ACOX2: a multi-omics discovery of a novel biomarker in IgA nephropathy. Hereditas. (2026) 163:21. doi: 10.1186/s41065-026-00639-0 41540492 PMC12888180

[273] Ghoreishifar M Macleod IM Nguyen T Lopdell TJ Littlejohn MD Xiang R . Bridging GWAS to genes: an integrative multi-omics approach using cattle data. BMC Genomics. (2026) 27:171. doi: 10.1186/s12864-026-12525-0 41535759 PMC12888714

[274] Zhou Z Zhang Y Liu S Tang H Yang L Lu Y . Ferroptosis in Alzheimer's disease: molecular mechanisms and advances in therapeutic strategies. Front Neurosci. (2025) 19:1673315. doi: 10.3389/fnins.2025.1673315 41601548 PMC12832829

[275] van der Koog L Showell HRD Nugraha DF Lehmann M Conlon TM Yildirim A . Regenerative therapeutics for chronic obstructive pulmonary disease. Pharmacol Rev. (2026) 78:100124. doi: 10.1016/j.pharmr.2026.100124 41856010 PMC13197932

[276] Lin D Zhang L Huang C Shao W . Skeletal muscle metabolism in health and disease: Mechanisms, interventions, and clinical perspectives. iScience. (2026) 29:115024. doi: 10.1016/j.isci.2026.115024 41858896 PMC12996713

[277] Pei WB Lei ZY Xie FQ Chen P Su YC Qin YS . A novel photosensitizer berberine derivative B12 induces apoptosis and suppresses HIF-1α expression in colorectal cancer cells via mitochondria-to-nucleus sequential-targeting. Acta Pharmacol Sin. (2026) 47:1249–69. doi: 10.1038/s41401-025-01728-y 41559402 PMC13109362

[278] Zhou Y Chen C Wang Z Wu F Tian D Xiao Z . Efficacy and safety of ensifentrine in Chinese patients with chronic obstructive pulmonary disease: The ENHANCE-CHINA randomized clinical trial. Chest. (2026) S0012-3692(26)00414–9. doi: 10.1016/j.chest.2026.02.035 41895581

[279] Jiang X Xu B Xu R Wu N Tian S Peng H . A comprehensive analysis of dry eye disease clinical trials (2000-2024): research trends and gaps. Front Pharmacol. (2026) 17:1713433. doi: 10.3389/fphar.2026.1713433 41613775 PMC12847023

[280] Xu JJ Yan CZ Liu ZQ Sun HR Zhang MY Huang QP . Vitexin reduced the dihydrotestosterone (DHT)-induced fibrosis in KGN cells by regulating the NR4A1/NLRP3 pathway. J Steroid Biochem Mol Biol. (2026) 257:106927. doi: 10.1016/j.jsbmb.2025.106927 41482075

[281] Zhu Y Wu X Peng X He H Hu Z Xiao L . The role of ferroptosis in osteoporosis: from pathogenic mechanisms to natural product-driven therapeutic innovations. Front Med (Lausanne). (2025) 12:1713327. doi: 10.3389/fmed.2025.1713327 41551515 PMC12808412

[282] Brunello G Lin GH Kopp I Carrasco-Labra A Jung RE Wang HL . 1st global consensus for clinical guidelines: Identifying a core outcome set for implant dentistry in edentulous maxilla rehabilitation. Clin Oral Implants Res. (2026) 37 Suppl 30:S108–s120. doi: 10.1111/clr.70075 41732050 PMC12930137

[283] Naas S Fekete M Szendro G Komaromi T Rozgonyi Z Palmer E . Multimodal therapeutic strategies for the management of sarcopenia and frailty in chronic obstructive pulmonary disease: A narrative review. Nutrients. (2026) 18:543. doi: 10.3390/nu18030543 41683365 PMC12899587

[284] Sompa SI Upadhyay S Ganguly K Bergström A Ji J Palmberg L . Epithelial effects of exposure to e-cigarettes alone or in combination with cigarette smoke using multicellular lung mucosa models. Environ Toxicol Pharmacol. (2026) 124:104989. doi: 10.1016/j.etap.2026.104989 41812846

[285] Sighinolfi S Cassina L Lidonnici MR Beretta S Stefanoni D Storto M . Iron overload damages mitochondria and induces metabolic rewiring of hematopoietic stem cells towards glycolysis. Blood. (2026) 147:3217–30. doi: 10.1182/blood.2025031552 41878806 PMC13389870

[286] Lee JS . Evolving molecular subtypes of gastric cancer: From past classifications to present consensus and future directions for precision therapy. J Gastric Cancer. (2026) 26:16–30. doi: 10.5230/jgc.2026.26.e12 41517845 PMC12802024

[287] Shi FL Lv YF Luo SQ Sun YX Wen XQ Ren YX . Luteolin mitigates inflammatory organ injury by targeting XIAP to block PANoptosis and mitochondrial dysfunction. Int Immunopharmacol. (2026) 173:116271. doi: 10.1016/j.intimp.2026.116271 41637826

